# Lipidomics profiling of goose granulosa cell model of stearoyl-CoA desaturase function identifies a pattern of lipid droplets associated with follicle development

**DOI:** 10.1186/s13578-021-00604-6

**Published:** 2021-05-22

**Authors:** Xin Yuan, Shenqiang Hu, Liang Li, Chunchun Han, Hehe Liu, Hua He, Lu Xia, Jiwei Hu, Bo Hu, Mingxia Ran, Yali Liu, Jiwen Wang

**Affiliations:** Country Farm Animal Genetic Resources Exploration and Innovation Key Laboratory of Sichuan Province, College of Animal Science and Technology, Sichuan Agricultural University, Chengdu, 611130 People’s Republic of China

**Keywords:** SCD, Gene expression, Goose granulosa cells, Ovarian follicles, Lipid droplets

## Abstract

**Background:**

Despite their important functions and nearly ubiquitous presence in cells, an understanding of the biology of intracellular lipid droplets (LDs) in goose follicle development remains limited. An integrated study of lipidomic and transcriptomic analyses was performed in a cellular model of stearoyl-CoA desaturase (SCD) function, to determine the effects of intracellular LDs on follicle development in geese.

**Results:**

Numerous internalized LDs, which were generally spherical in shape, were dispersed throughout the cytoplasm of granulosa cells (GCs), as determined using confocal microscopy analysis, with altered SCD expression affecting LD content. GC lipidomic profiling showed that the majority of the differentially abundant lipid classes were glycerophospholipids, including PA, PC, PE, PG, PI, and PS, and glycerolipids, including DG and TG, which enriched glycerophospholipid, sphingolipid, and glycerolipid metabolisms. Furthermore, transcriptomics identified differentially expressed genes (DEGs), some of which were assigned to lipid-related Gene Ontology slim terms. More DEGs were assigned in the SCD-knockdown group than in the SCD-overexpression group. Integration of the significant differentially expressed genes and lipids based on pathway enrichment analysis identified potentially targetable pathways related to glycerolipid/glycerophospholipid metabolism.

**Conclusions:**

This study demonstrated the importance of lipids in understanding follicle development, thus providing a potential foundation to decipher the underlying mechanisms of lipid-mediated follicle development.

**Supplementary Information:**

The online version contains supplementary material available at 10.1186/s13578-021-00604-6.

## Background

Lipids are a highly diverse class of molecules key to many cellular processes, including energy storage, structural moieties, signaling molecules, and protein modification [1, 2]. Lipids have been loosely defined as biological substances that are generally hydrophobic in nature and, in many cases, soluble in organic solvents. These chemical properties include a broad range of molecules, including fatty acids, phospholipids, sterols, sphingolipids, terpenes, with each having a role in living systems [3]. Lipid metabolism involves numerous interwoven pathways; these networks are essential for regulating cell function, which is influenced by many external factors. Imbalanced changes in lipid composition can even affect various physiological functions of the cell [4]. Lipid overload results in lipotoxicity, which initially manifests as organelle dysfunction, ultimately leading to cell death and tissue damage [5, 6]. Evidently, deregulation of this network contributes to the onset of pathology as lipids are tightly regulated, both spatially and temporally, in various parts of the organism [7, 8]. Many lipid molecular species have been identified as potential biomarkers involved in several diseases, including, epidemic diseases, cancers, inflammation, and genetic diseases [9].

Intracellular lipid droplets (LDs) occur through the deposition of lipid complex molecular pathways. They are found in a wide variety of cell types, and are viewed as highly dynamic organelles with unique spherical structures, of which primary neutral lipids are mainly sterol esters and triacylglycerols (TGs) [10, 11]. LDs affect cell and organ health and contribute to the dynamics of biological membranes, energy storage and expenditure, and conveying messages at all cellular levels, including in the nucleus [12, 13]. Despite their important functions and nearly ubiquitous presence in cells, many aspects of LD biology are unknown. We have previously confirmed that de novo lipogenesis occurs in goose GCs [14] and that LD accumulation capacity depends on the stage of granulosa cells (GCs) during goose follicle development [15]. It has been reported that lipid metabolism in bovine, sheep, and human GCs is pivotal for oocyte maturation, fertilization, and early embryonic development [16–19]. Lipid content in follicles differs among species, as reported in studies performed in porcine [20], bovine [21], and canine oocytes [22], wherein LD number, size, and distribution were analyzed. However, an understanding of the biology of LDs in GCs remains limited. Recently, the role of stearoyl-CoA desaturase (SCD) as a regulator of fatty acid desaturation in goose GCs was identified, whereby metabolite changes in lipid-related metabolism were found to be closely related to follicular development [23]. Although this observation indicated that such links might exist, it is unclear whether LDs play a role in goose follicle development and which lipid species or classes might be affected. It is therefore hypothesized that lipidomic analysis of the goose GCs at the level of individual lipid molecular species might help to elucidate such a mechanism.

Lipidomics comprises the characterization of an organism’s differences in individual lipid molecular species and their biological roles with respect to the expression of proteins involved in lipid metabolism and function [24, 25]. Thus, to obtain a detailed view of lipid metabolism, it is crucial to analyze the full lipid profiles of the individual lipids using a lipidomics approach. This is the first time that a combination of lipidomics and transcriptomics analysis has been applied to the GC model of SCD function to identify alterations in the regulation of specific gene, lipid classes, and lipid-mediated signaling processes that are involved in LDs associated with follicle development in geese. The detailed characterization of lipid classes and species provided in this study might be useful to understand the mechanism of intramyocellular LDs, and an analysis based on integrating two-omics datasets will broaden our understanding of lipid alterations with respect to lipid-gene networks in GCs associated with follicle development.

## Results

### LD distribution in goose GCs

**Fig. 1:**
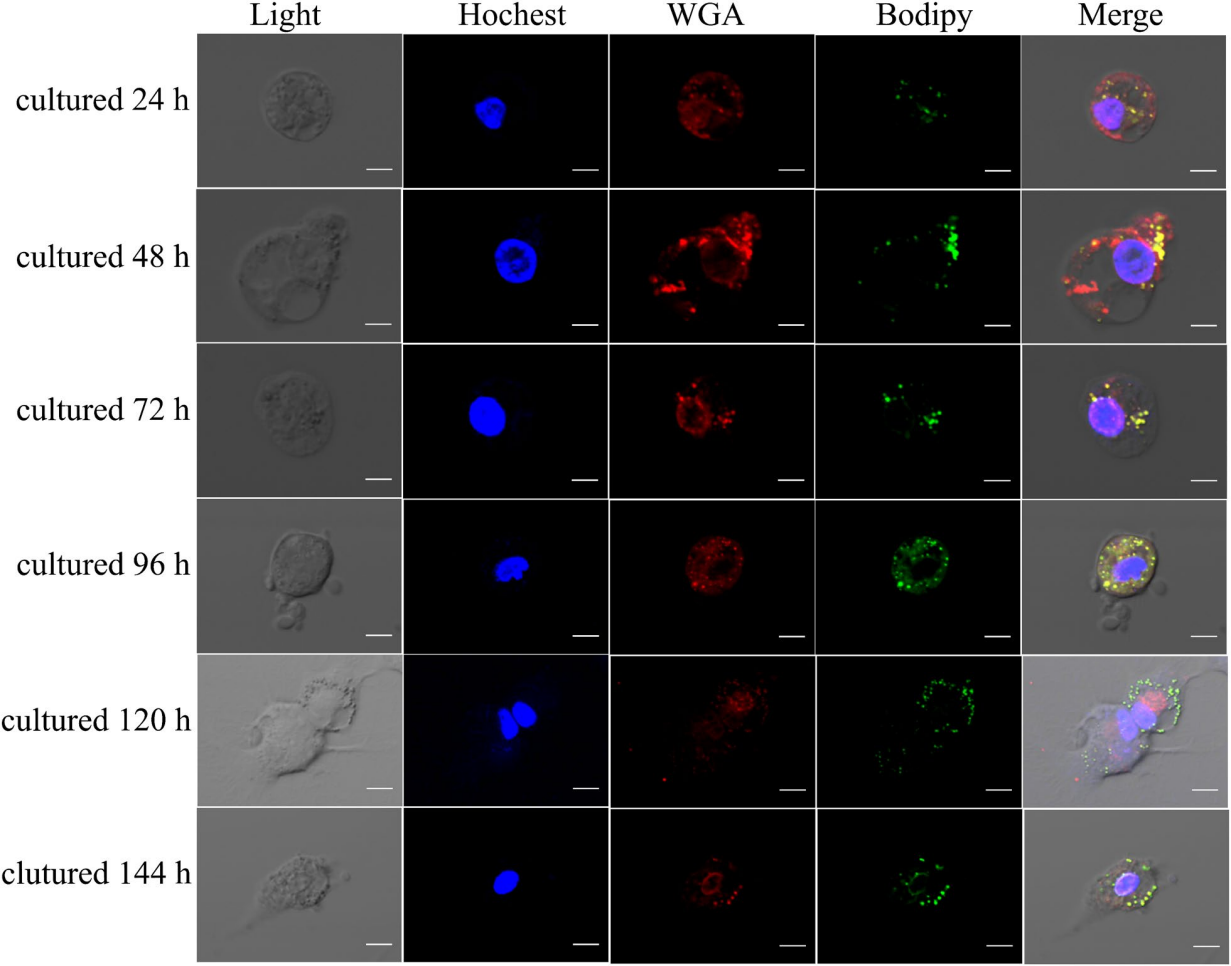
Distribution of lipid droplets (LDs) and their colocalization in live goose granulosa cells. LDs were stained with the neutral lipid dye Bodipy 500/510 (green), cell membrane were stained with WGA (red), the nuclei were stained with Hoechst 33258 (blue). All images are equal magnification, scale bar indicates 40 μm

Previous studies have demonstrated that de novo lipogenesis occurs in goose ovarian GCs, which have lipid accumulation capacity [15]. To investigate the localization of LDs in goose GCs, confocal microscopy was used to analyze LD location in goose GCs stained with BODIPY (500/510, green). GCs contained numerous internalized LDs that were generally spherical in shape and dispersed throughout the cytoplasm (Fig. 1). The dispersed LDs clumped to form clusters. The mobility of the dispersed LDs was from the perinuclear regions to the extracellular domain.

### Altered SCD expression affects LD content

**Fig. 2:**
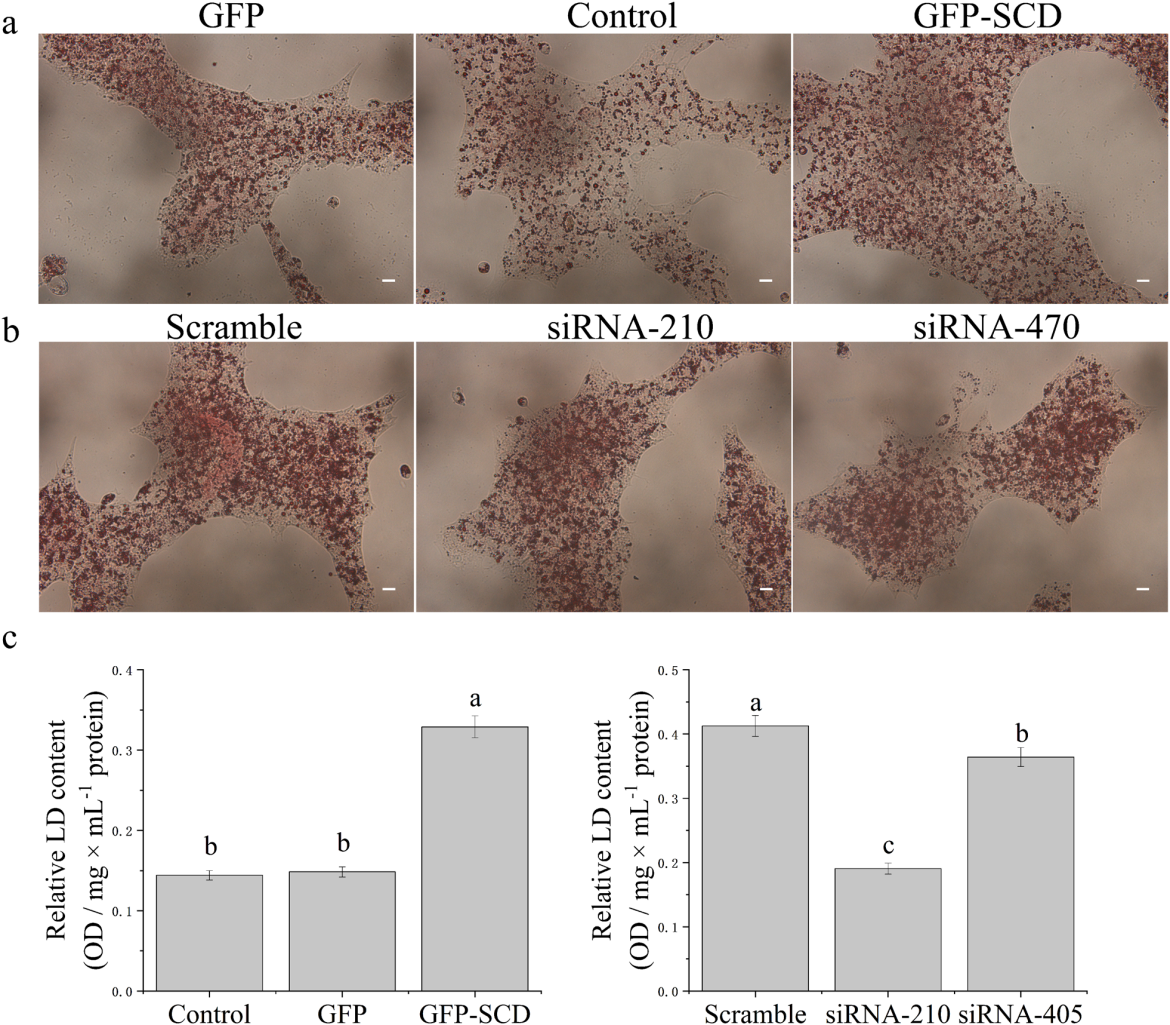
After culturing, cells were fixed with formaldehyde and stained with Oil red O, scale bar indicates 200 μm. **a** The cells were after SCD expression. **b** The cells were after SCD knockdown. **c** Relative LD content. The results are shown as mean ± SD from six experiments, the data were analyzed by ANOVA and Tukey’s test. The lowercase letter indicates significant differences between each group (*P* < 0.05)

To further investigate the effect of SCD expression on LDs in GCs, the LDs were stained with Oil Red O after SCD-overexpression and knockdown (Fig. 2a, b). The accumulation of LD content was quantified using appropriate equations (see “Materials and methods” section for details). Compared with that in the control group, the overexpression of SCD significantly stimulated the accumulation of LD content. However, SCD knockdown significantly inhibited the accumulation of LDs (Fig. 2c).

### Analysis of lipid classes

**Fig. 3:**
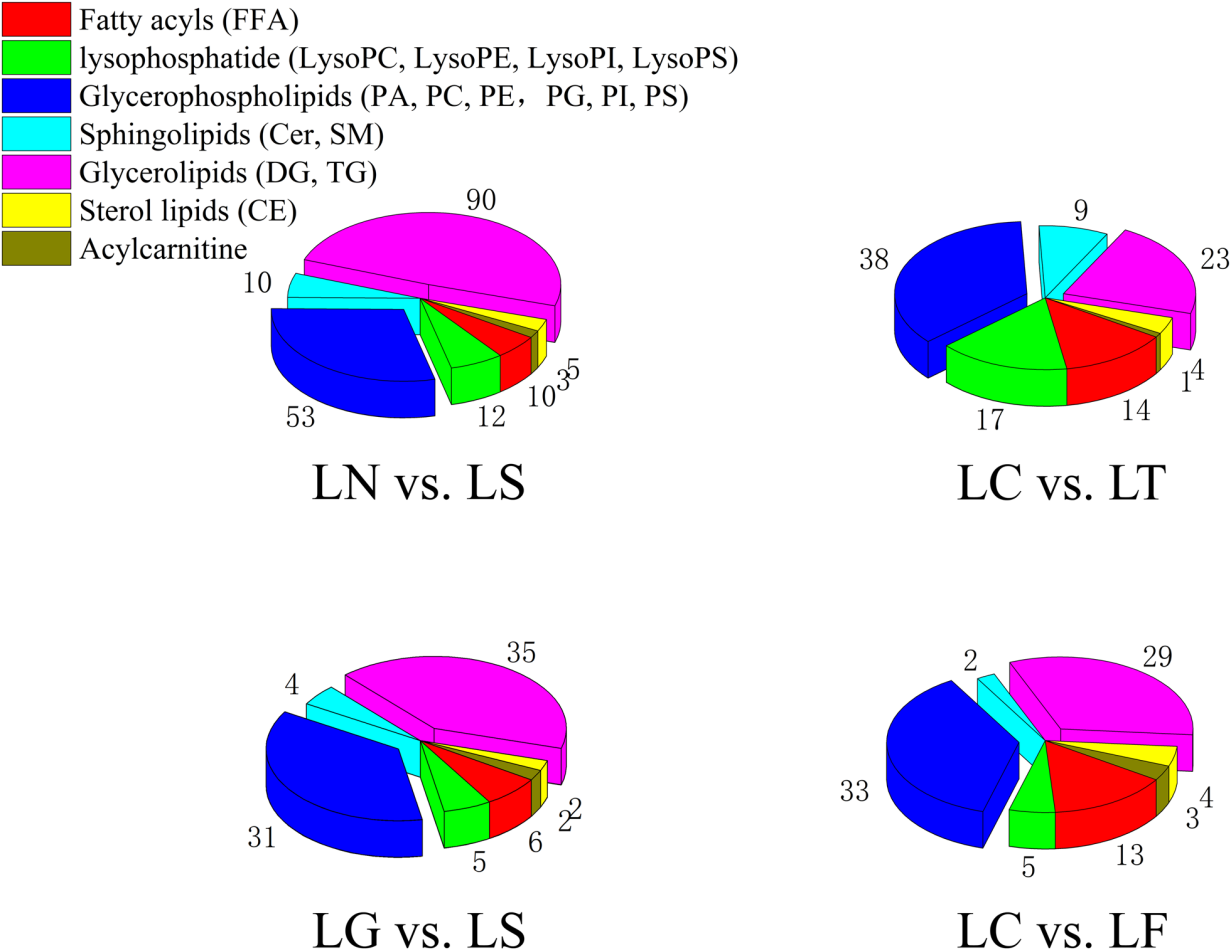
Lipidomics studies was performed in a cellular model of SCD function of goose GCs, pie chart shows number of significantly disturbed lipids of each group

**Fig. 4:**
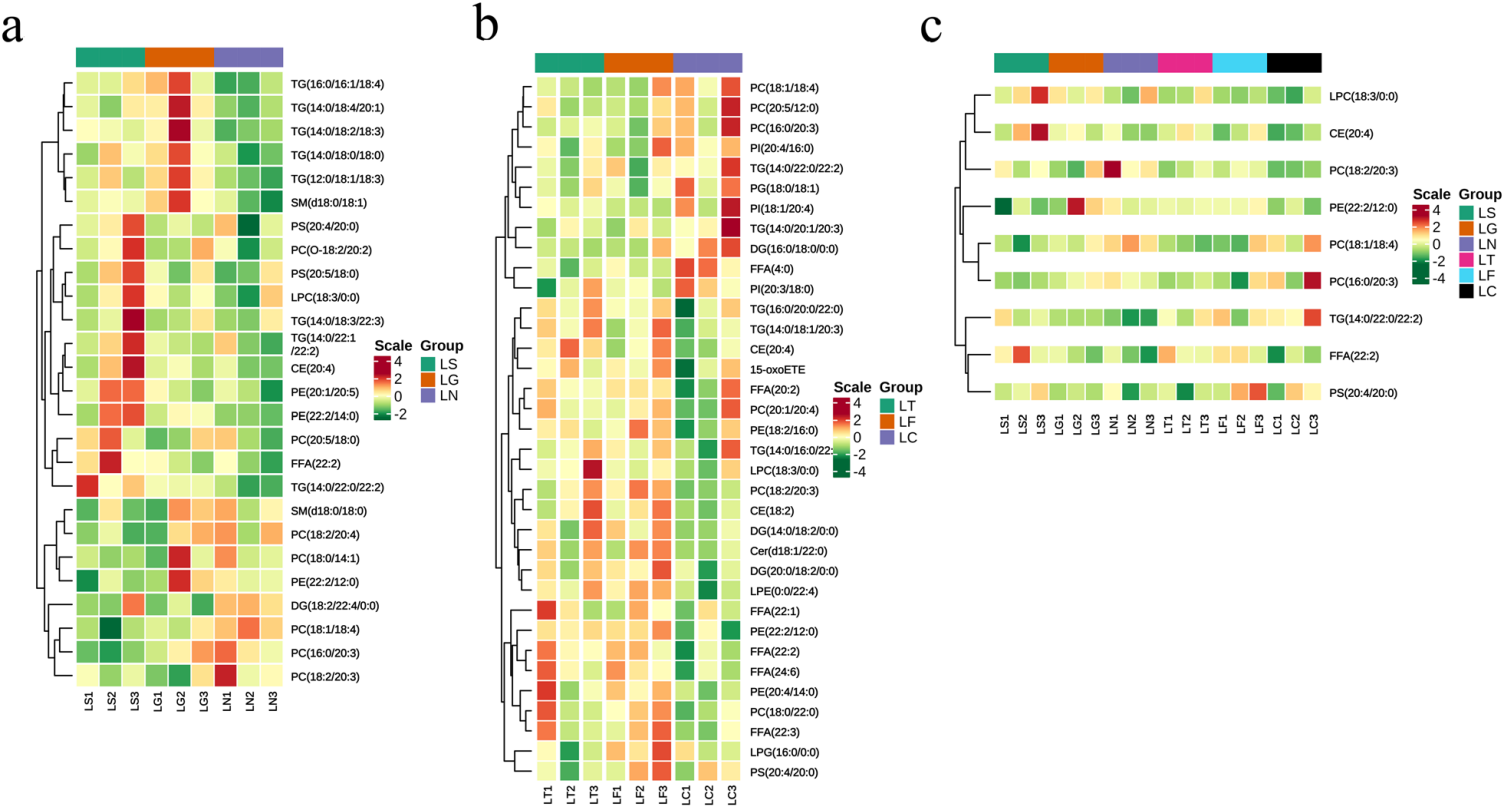
Heat map from the hierarchical clustering of differential overlapping lipid in the (**a**) SCD-overexpression group. **b** SCD-knockdown group. **c** SCD-overexpression and SCD-knockdown groups. The scaled expression by row (lipids) is shown as a heat map and is reordered by a hierarchical clustering analysis (Pearson’s distance and Ward’s method) on both rows and columns. The color scale indicates the relative amounts of metabolites: red, higher levels; green, lower levels

In total, 662 species of lipids were identified in the GC samples using LC–MS/MS analysis (Additional file 1: Fig. S1). There were 183 different lipids identified in the control group (referred to as LN) vs. SCD-overexpression group (referred to as LS) comparison (165 upregulated and 18 downregulated) based on fold-change analysis (variable importance in projection [VIP] > 1, fold-change [FC] > 1.2), 85 lipids in the GFP vector group (referred to as LG) vs. LS comparison (36 upregulated and 49 downregulated), 90 lipids in the scrambled siRNA group (referred to as LC) vs. siRNA-210 (referred to as LT) comparison (45 upregulated and 45 downregulated), and 106 lipids in the LC vs. siRNA-470 (referred to as LF) comparison (86 upregulated and 20 downregulated). Glycerophospholipids and glycerolipids were the predominant lipid species (Fig. 3; Additional file 6: Table S1). Venn diagram analysis revealed 26 overlapping lipids in the SCD-overexpression group, 35 overlapping lipids in the SCD-knockdown group, and nine overlapping lipids in the SCD-overexpression and SCD-knockdown groups (Additional file 2: Fig. S2). As shown in Table 1 and Fig. 4, 19 lipids followed the same trend, whereas seven lipids showed the opposite pattern, as compared to that of the two overexpressing groups (LN vs. LS comparison and LG vs. LS comparison). Thirty-three lipids followed the same trend, whereas two lipids showed the opposite pattern, as compared to that of the two knockdown groups (LC vs. LT comparison and LC vs. LF comparison). Seven lipids followed the same trend, whereas two lipids showed the opposite pattern between the overexpressed and knockdown groups.

**Table 1:** Statistical analysis of differential lipids for four cross-comparisons

Molecular species	Vip	Fold change	*p*-vaule	Vip	Fold change	*p*-vaule
	Comparison I: LN vs. LS comparison			Comparison II: LG vs. LS comparison		
FFA(22:2)	1.64	1.81	0.094	2.38	1.54	0.141
PC(18:0/14:1)	1.56	0.83	0.219	1.21	0.83	0.467
PC(16:0/20:3)	2.10	0.68	0.038	2.83	0.71	0.054
PC(18:1/18:4)	1.97	0.61	0.032	2.07	0.77	0.168
PC(20:5/18:0)	1.25	1.28	0.251	1.81	1.29	0.224
PC(18:2/20:3)	1.46	0.64	0.259	1.12	1.26	0.626
CE(20:4)	1.42	1.52	0.198	1.71	1.32	0.321
DG(18:2/22:4/0:0)	1.38	0.79	0.341	1.37	1.27	0.440
LPC(18:3/0:0)	1.21	1.52	0.334	1.04	1.23	0.503
PC(18:2/20:4)	1.38	0.67	0.163	1.46	0.75	0.354
PC(O-18:2/20:2)	1.28	1.53	0.228	1.16	1.24	0.480
PE(22:2/12:0)	1.37	0.55	0.158	2.01	0.43	0.171
PE(22:2/14:0)	1.70	1.55	0.145	1.36	1.21	0.386
PE(20:1/20:5)	1.52	1.87	0.081	2.33	1.55	0.131
PS(20:4/20:0)	1.04	1.23	0.359	2.23	1.22	0.199
PS(20:5/18:0)	1.36	1.40	0.239	1.33	1.23	0.389
SM(d18:0/18:0)	1.13	0.71	0.306	1.14	0.71	0.444
SM(d18:0/18:1)	1.52	1.42	0.156	2.20	0.78	0.195
TG(14:0/18:0/18:0)	1.53	1.41	0.154	1.52	0.83	0.315
TG(14:0/22:0/22:2)	1.94	2.12	0.035	2.13	1.48	0.195
TG(14:0/22:1/22:2)	1.20	1.57	0.251	2.13	1.48	0.233
TG(12:0/18:1/18:3)	1.73	1.42	0.107	1.95	0.79	0.196
TG(14:0/18:2/18:3)	2.09	1.60	0.012	1.58	0.76	0.369
TG(16:0/16:1/18:4)	1.87	1.51	0.048	1.61	0.82	0.288
TG(14:0/18:4/20:1)	1.38	1.51	0.195	2.09	0.64	0.179
TG(14:0/18:3/22:3)	1.06	1.80	0.404	1.08	1.56	0.488

	Comparison III: LC vs. LT comparison			Comparison IV: LC vs. LF comparison		
15-oxoETE	1.49	1.41	0.465	1.32	1.52	0.376
FFA(4:0)	2.58	0.63	0.048	2.20	0.71	0.707
FFA(22:1)	1.61	1.38	0.389	1.05	1.22	0.510
FFA(20:2)	1.62	1.50	0.560	1.38	1.66	0.458
FFA(22:2)	2.44	1.65	0.089	2.01	1.66	0.077
FFA(22:3)	1.81	1.26	0.331	1.97	1.44	0.105
FFA(24:6)	2.06	2.41	0.154	1.75	2.51	0.074
LPG(16:0/0:0)	1.37	0.66	0.459	1.91	1.73	0.091
PC(16:0/20:3)	1.78	0.63	0.300	1.34	0.63	0.331
PC(18:1/18:4)	2.59	0.66	0.092	1.48	0.76	0.304
PC(18:2/20:3)	2.22	1.63	0.155	2.48	1.97	0.016
PC(20:1/20:4)	1.37	1.22	0.646	1.19	1.27	0.583
PE(18:2/16:0)	1.71	1.20	0.375	1.79	1.35	0.183
PE(20:4/14:0)	1.73	1.49	0.341	2.21	1.65	0.019
PG(18:0/18:1)	1.90	0.72	0.230	1.80	0.63	0.141
PI(20:3/18:0)	1.84	0.64	0.336	1.96	0.65	0.133
PI(20:4/16:0)	1.77	0.67	0.212	1.36	0.74	0.482
PI(18:1/20:4)	1.56	0.77	0.336	1.83	0.65	0.205
CE(18:2)	1.91	1.36	0.214	2.11	1.40	0.079
CE(20:4)	2.68	1.48	0.037	1.40	1.27	0.310
Cer(d18:1/22:0)	1.87	1.25	0.251	1.81	1.33	0.184
DG(16:0/18:0/0:0)	2.70	0.75	0.076	1.66	0.82	0.181
DG(14:0/18:2/0:0)	1.42	1.37	0.356	2.08	1.51	0.068
DG(20:0/18:2/0:0)	1.47	1.32	0.319	1.77	1.55	0.102
LPC(18:3/0:0)	1.75	1.62	0.276	1.05	1.20	0.578
LPE(0:0/22:4)	2.32	1.20	0.081	2.18	1.25	0.045
PC(18:0/22:0)	1.82	1.21	0.338	2.12	1.31	0.063
PC(20:5/12:0)	1.82	0.80	0.236	1.48	0.82	0.279
PE(22:2/12:0)	2.47	1.37	0.127	2.12	1.45	0.079
PS(20:4/20:0)	1.12	0.79	0.417	1.19	1.24	0.391
TG(14:0/16:0/22:0)	1.33	1.31	0.701	1.14	1.37	0.643
TG(16:0/20:0/22:0)	1.57	1.67	0.332	1.26	1.48	0.465
TG(14:0/22:0/22:2)	1.78	0.77	0.273	1.16	0.80	0.429
TG(14:0/18:1/20:3)	2.33	1.65	0.082	1.26	1.42	0.364

*p*-vaule from Student’s t test

*VIP* variable importance in the projection, *FFA* free fat acid, *CE *cholesteryl ester, *DG* diacylglycerol, *TG* triglyceride, *PC* phosphatidylcholine, *PE* glycerophosphoethanolamine, *PS* glycerophosphoserine, *PG* glycerophosphoglycerol, *PI* glycerophosphoinositol, *LPC* lysophosphatidylcholine, *LPG* lysophosphatidylglycerol, *Cer* ceramide, *SM* sphingomyelin

### Cross comparisons between and within each group

**Fig. 5:**
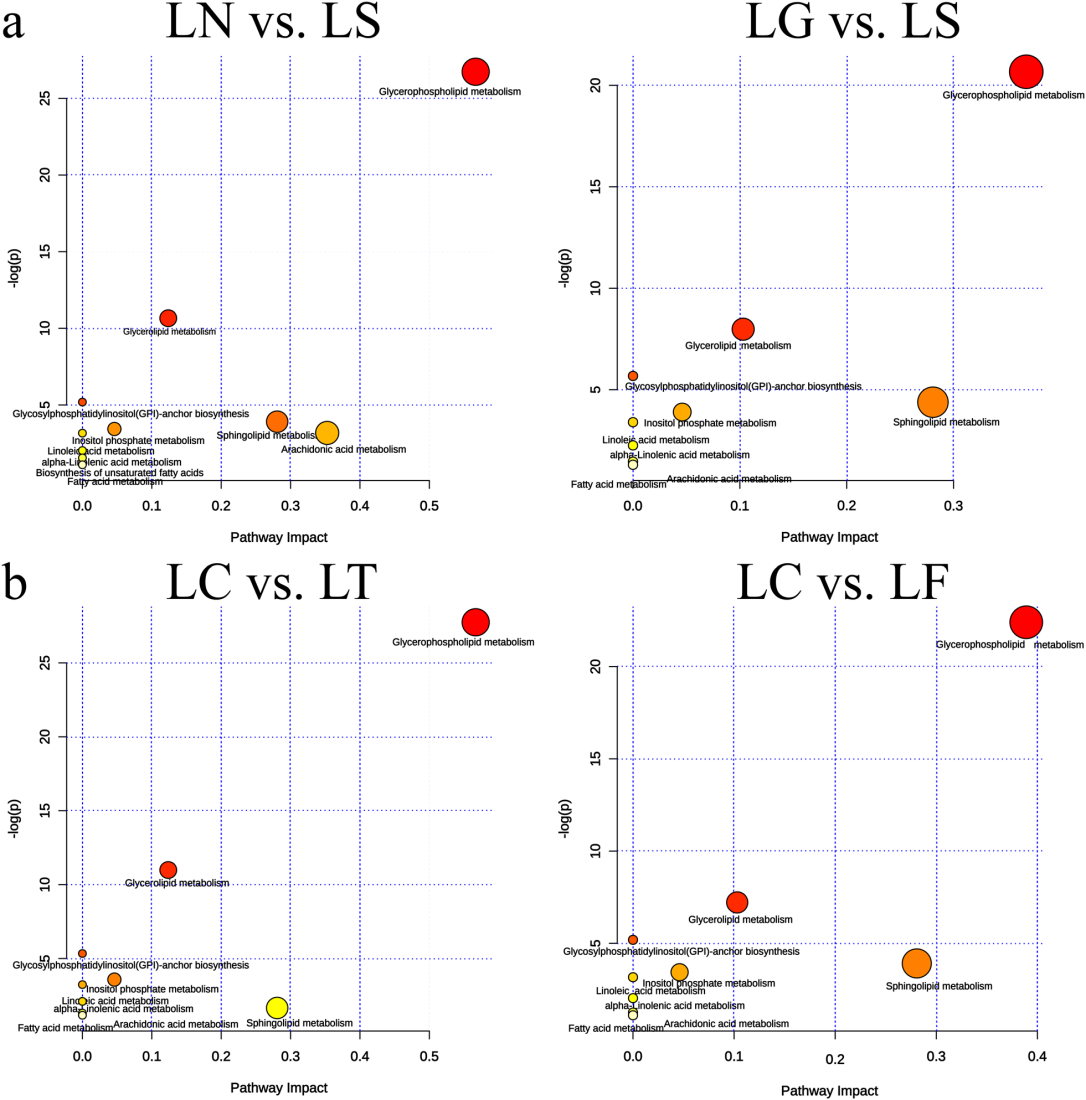
Disturbed metabolic pathways. Metaboanalyst (http://www.metaboanalyst.ca) generated topology map described the impact of baseline metabolites identified with high VIP values (VIP > 1) on metabolic pathways between (**a**) Overexpressed group, LN vs. LS comparison and LG vs. LS comparison. **b** Knockdown group, LC vs. LT comparison and LC vs. LF comparison

With the aid of the KEGG database, metabolic pathways are summarized in Additional file 7: Table S2 based on the identified lipids. In total, 183 lipids were associated with 57 metabolic pathways in the LN vs. LS comparison, 85 lipids with 40 metabolic pathways in the LG vs. LS comparison, 90 lipids with 46 metabolic pathways in the LC vs. LT comparison, and 106 lipids with 42 metabolic pathways in the LC vs. LF comparison. Figures 5a, b represent the perturbed SCD-associated pathways, the results of which are as follows: for LN vs. LS, glycerophospholipid, arachidonic, sphingolipid, and glycerolipid metabolisms altered significantly; for LG vs. LS, glycerophospholipid, sphingolipid, glycerolipid, and inositol phosphate metabolisms altered significantly; for LC vs. LT, glycerophospholipid, sphingolipid, glycerolipid, and inositol phosphate metabolisms altered significantly; and for LC vs. LF, glycerophospholipid, sphingolipid, glycerolipid, and inositol phosphate metabolisms altered significantly.

### RNA-seq investigation of overexpression and knockdown of SCD expression in GCs

To obtain a comprehensive view of the role of the SCD gene in GCs, the six groups of cells (SCD-overexpression group [referred to as OS], GFP vector group [referred to as OG], control group [referred to as LN], siRNA-210 group [referred to as ST], siRNA-470 group [referred to as SF], scrambled siRNA group [referred to as LC]) were subjected to RNA-seq analysis. According to the RNA-seq data, 51.56, 53.78, 55.70, 57.70, 51.65, and 56.19 million clean reads were obtained from the NC, OS, OG, SC, ST, and SF samples, respectively. For each sample, 37.69, 38.67, 40.04, 42.60, 37.88, and 41.30 million unique reads could be mapped to the current version of the goose genome (*Anser cygnoides*) (Table 2). The basic characteristics of the tags in each sample library are shown in Additional file 8: Table S3.

**Table 2:** Statistic of the mapping of sequence to the reference genome goose

Terms	NC	OG	OS	SC	ST	SF
Clean reads (percentage^a^)	51,566,021 (96.10%)	53,774,948 (96.57%)	55,702,230 (96.37%)	57,694,766 (96.62%)	51,644,681 (96.54%)	56,187,441 (96.41%)
Reads mapped (percentage^b^)	38,589,491 (74.91%)	39,550,763 (73.60%)	40,979,329 (73.37)	43,575,018 (75.62%)	38,710,384 (75.00%)	42,250,540 (75.19%)
Uniquely mapped	37,687,479 (73.16%)	38,664,803 (71.96%)	40,043,890 (71.69)	42,597,240 (73.92%)	37,884,321 (73.40%)	41,295,405 (73.49%)

^a^The number of clean reading frames of total raw reading frames

^b^The number of all mapped reads out of total clean reads

### Identification and functional enrichment of DEGs

**Fig. 6:**
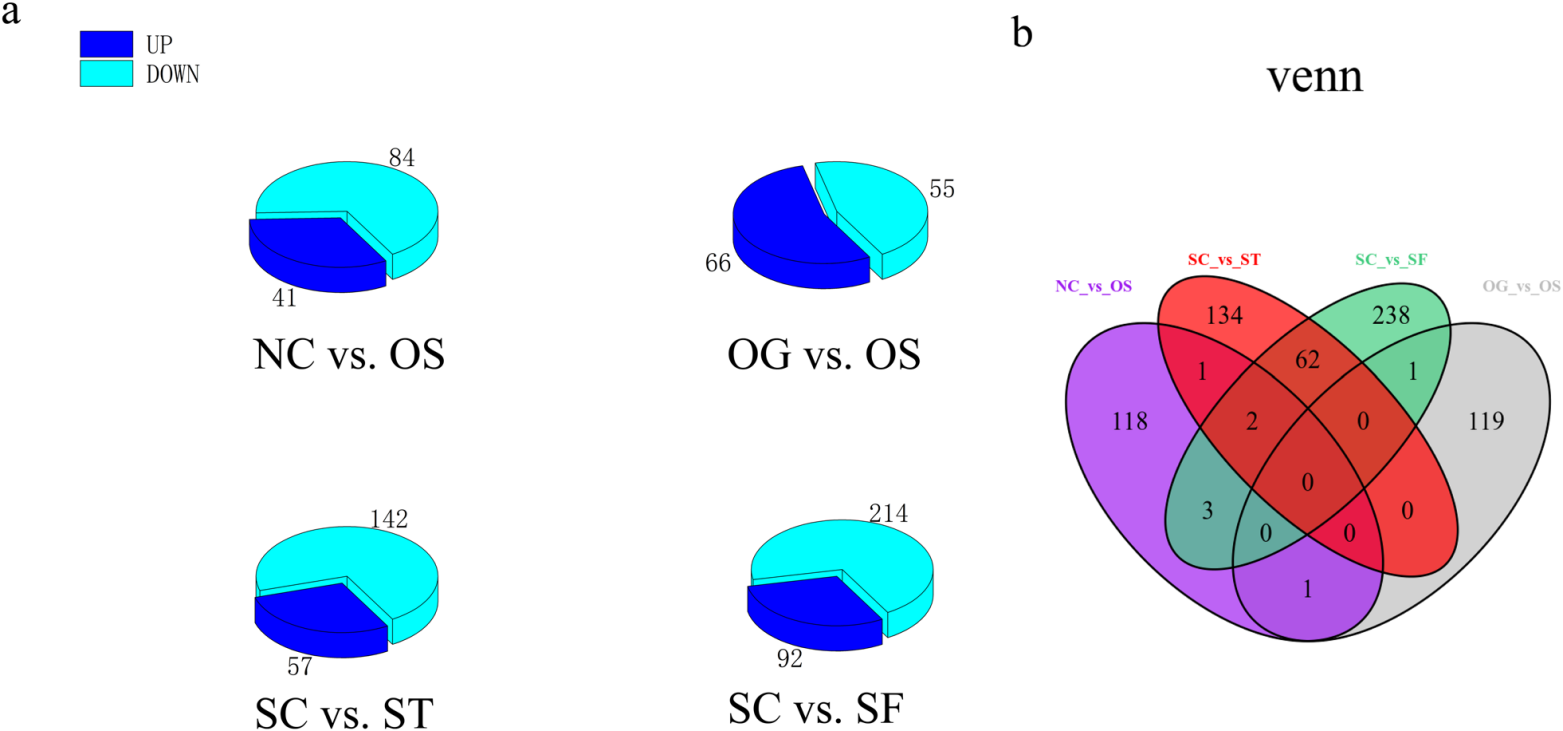
Transcriptomics studies was performed in a cellular model of SCD function of goose GCs. **a** Pie chart shows number of significantly disturbed DEGs of each group. **b** Venn diagram of overlapping and unique of DEGs altered in each group

In total, 679 DEGs were identified in the GCs after SCD overexpression and knockdown (Additional file 3: Fig. S3), including 125 DEGs (41 upregulated and 84 downregulated) in the NC vs. OS comparison, 121 DEGs (66 upregulated and 55 downregulated) in the OG vs. OS comparison, 199 DEGs (57 upregulated and 142 downregulated) in the SC vs. ST comparison, and 306 DEGs (92 upregulated and 214 downregulated) in the SC vs. SF comparison (Fig. 6a, b).

**Fig. 7:**
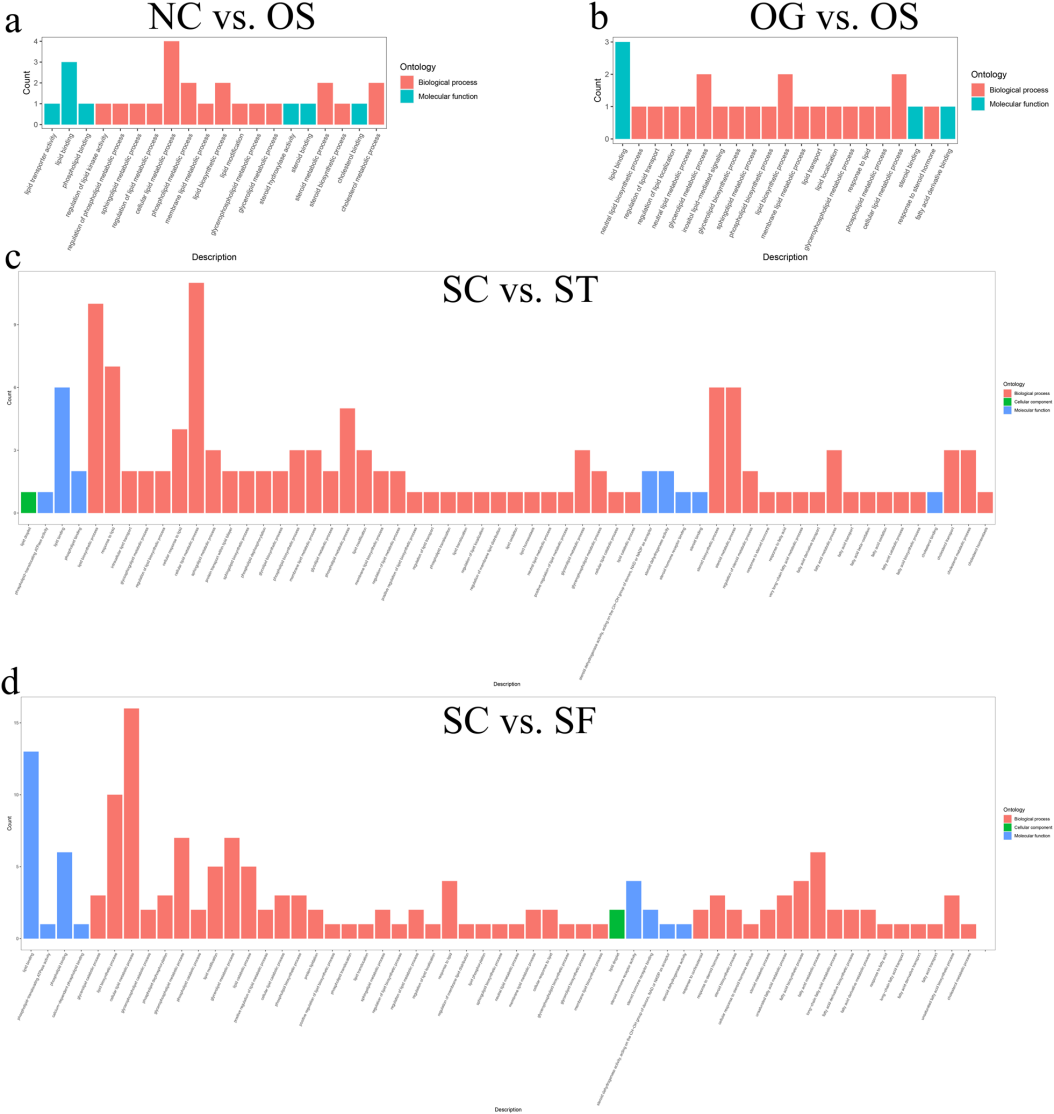
Histogram presentation of the lipid-related gene ontology classification of the DEGs. **a** NC vs. OS comparison. **b** OG vs. OS comparison. **c** SC vs. ST comparison. **d** SC vs. SF comparison. The results of GO classifications are provided in Additional file 9: Table S4

To characterize differences in lipid-related genes between the transcriptomes of the SCD-overexpression and SCD-knockdown groups, the GO slim terms of the complex Gene Ontology annotations were used. The results showed that DEGs were assigned to 20 lipid-related GO terms in the NC vs. OS comparison, 21 lipid-related GO terms in the OG vs. OS comparison, 63 lipid-related GO terms in the SC vs. ST comparison, and 63 lipid-related GO terms in the SC vs. SF comparison (Fig. 7; Additional file 9: Table S4). The top 20 pathways of KEGG functional analysis showed that SCD-overexpression had a significant effect on one carbon pool by folate, glycosphingolipid biosynthesis, ferroptosis, and influenza A. Knockdown of SCD had a significant effect on sphingolipid metabolism, proteasome, mTOR signaling pathway, hedgehog signaling pathway, glycosphingolipid biosynthesis (metabolism), ether lipid metabolism, amino sugar and nucleotide sugar metabolism, and protein processing in the endoplasmic reticulum (Additional file 4: Fig. S4a, b).

### Level of coordination between transcript and lipidomic data

**Fig. 8:**
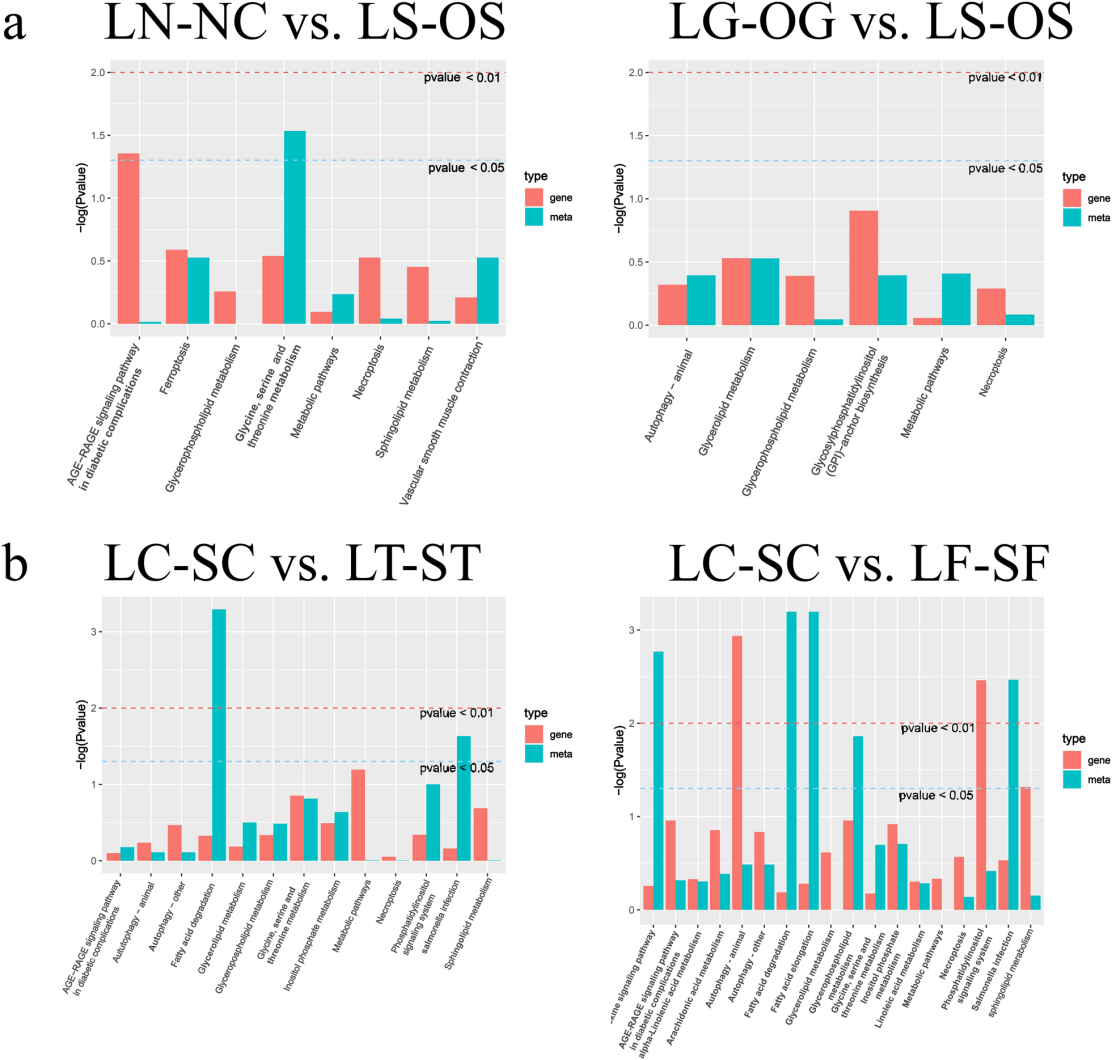
Two-omics datasets into pathway enrichment analysis. **a** LN-NC vs. LS-OS comparison and LG-OG vs. LS-OS comparison. **b** LC-SC vs. LT-ST comparison and LC-SC vs. LF-SF comparison

**Fig. 9:**
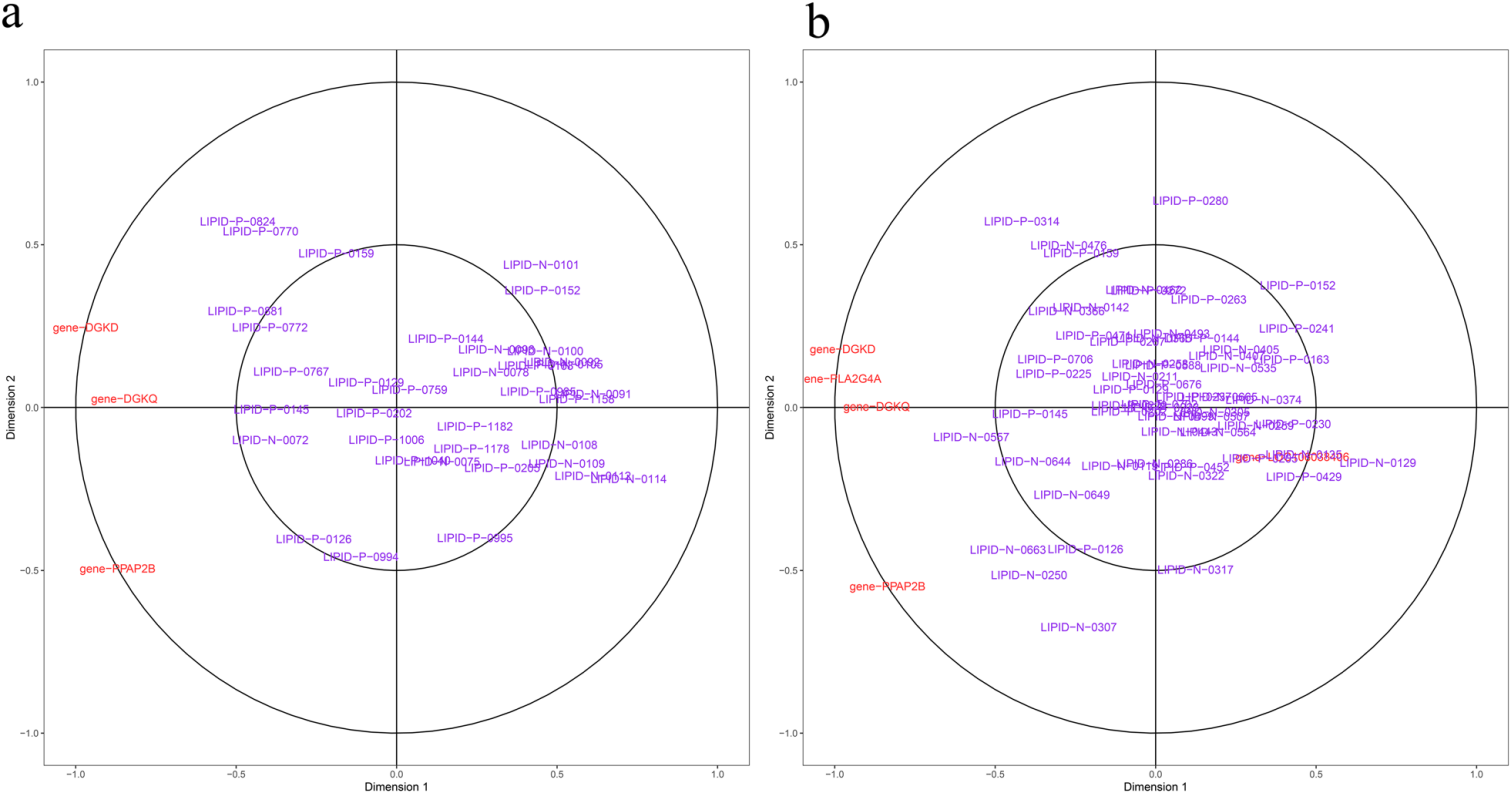
Visualization of the CCA results of lipids and DEGs involved in (**a**) glycerolipid metabolism and (**b**) glycerophospholipid metabolism under SCD knockdown. The results of lipids are provided in Additional file 10: Table S5

Monitored responses in parallel on the transcriptome and the lipid level were carried out, thus allowing one to compare the level of coordination between both molecular readouts. To perform this analysis, a holistic co-clustering approach was performed, as shown in Fig. 8. Perturbed SCD in GCs influenced mRNAs and lipids in glycerolipid/glycerophospholipid metabolism, glycine, serine, and threonine metabolism, and sphingolipid metabolism, which are associated with lipid-related metabolism pathways. However, SCD knockdown in GCs influenced more complicated lipid-related metabolism pathways, including fatty acid degradation/elongation, inositol phosphate metabolism, phosphatidylinositol signaling system, alpha-linolenic acid metabolism, arachidonic acid metabolism, and linoleic acid metabolism. Additionally, a targeted approach using prior biological knowledge in conjunction with canonical correlation analysis (CCA) revealed that lipids from glycerolipid/glycerophospholipid metabolism were subjected to a CCA together with transcript data of all those pathways as derived from SCD knockdown in GCs (Fig. 9, Additional file 10: Table S5). We were also interested in other lipid-related pathways subjected to a CCA together with transcript data (Additional file 4: Fig. S4). However, no similar CCA results were observed with SCD overexpression in GCs.

## Discussion

In the present study, we used a GC model of SCD function and performed a detailed study on cellular lipid metabolism using lipidomic profiling to evaluate the lipid molecular changes in a detailed manner. The method is based on ultra-high performance liquid chromatography combined with quadrupole-time-of-flight mass spectrometry and gas chromatography mass spectrometry, which allow for the analysis of hundreds of molecular lipids from a single sample run [26]. More than 660 lipids were identified, including phospholipids, ceramides, diacylglycerol and triacylglycerols, cholesterol esters, and fatty acid compositions (Fig. 3). Among these lipids, glycerophospholipids and glycerolipids were the predominant species. Additionally, altered SCD expression had the greatest impact on glycerophospholipid metabolism, this lipid-related pathway contains the most key lipids, with more than six representative metabolites as follows: PC (16:0/20:3); PC (18:1/18:4); PC (18:2/20:3); LPC (18:3/0:0); PE (22:2/12:0); and PS (20:4/20:0). FFA (22:2) and TG (14:0/22:0/22:2) were representative metabolites of glycerolipid metabolism, and sphingolipid metabolism (Figs. 4, 5).

There is great diversity in the structures and functions of the vast array of lipid species, with intracellular levels of lipids tightly regulated by a network of metabolic pathways to sustain normal cellular functions [27]. Glycerophospholipids make up phospholipids, which play both structural and metabolic roles in cells. Therefore, the level and species of phospholipids might reflect the biochemical and physiological conditions of cells [28]. Phospholipids are important for determining LD formation and are attributed to the formation of large LD fusions [29]. Glycerolipid metabolism is altered by fatty acids and, induces LD formation [30]. Blocking PC synthesis causes elevated SREBP-1-dependent gene transcription and LD accumulation in mouse liver and human cells, and the ratio of PE to PC in the LD surface tends to affect the LD number and size [31, 32]. The present study provided the lipid species composition and lipid metabolism of the GC model of SCD function. Given the important biological activities of many lipids, such information could provide potential insights into the mechanisms of internalized lipid-mediated follicle development.

We further investigated whether altered SCD expression of GCs affects gene expression involved in lipid metabolism. We observed that altered SCD expression resulted in many DEGs assigned to lipid-related GO terms, including lipid binding, lipid transport, lipid localization, and LDs. Compared with those in the SCD-overexpression group, more DEGs were identified and assigned to lipid-related GO terms in the SCD-knockdown group (Fig. 7; Additional file 9: Table S4). These results indicated that SCD regulates the lipid metabolic processes during goose follicle development. Particularly, SCD knockdown might mobilize more genes associated with lipid metabolism. Combined analysis of lipid and gene expression profiles showed that more complicated lipid-related metabolism pathways were significantly clustered, especially in the SCD-knockdown group.

Numerous internalized LDs were dispersed throughout the cytoplasm of GCs (Figs. 1, 2). However, in steroidogenic tissues such as the ovary, the ovarian follicles of almost all mammals contain LDs, which are prominently and generally non-pathogenic, except in cases of genetic disorders of steroidogenesis [33]. We speculate that there is a relationship between LDs and energy supply or the storage of cholesteryl esters during goose follicle development. Cholesterol esters, particularly CE (20:4), were significantly upregulated in SCD-overexpression and knockdown groups (Fig. 4c). Previous results demonstrated intracellular metabolite changes in which cholesterol was mostly upregulated in the SCD-overexpression group, whereas androsterone was upregulated in the SCD-knockdown group [23].

LDs make dynamic contact with nearly every other organelle. Some contact activity might exist among the ER, endosomes, mitochondria, and peroxisomes in the cell. This contact is believed to mediate the transfer of lipids (including phospholipids, sterols, and fatty acids) between the different cellular compartments [34, 35]. Pharmacological inhibition of the enzymatic activity of fatty acid synthase significantly reduces the production of progesterone in bovine GCs, suggesting that ovarian steroidogenesis relies on lipid metabolism in GCs [16, 36]. Additionally, LD accumulation capacity of goose GCs depends on the different stages of follicle development, with the stage of the highest accumulation capacity being F1 GCs, followed by F4–F2 GCs and pre-hierarchical GCs [15]. As ovarian follicles grow with changes in both the size and number of LDs in bovines [37], the total intracellular LD content increases gradually with follicular growth. This exponential increase was demonstrated in large antral follicles in rats and goats [38, 39], with an increase in LD numbers and size in GCs within one day after ovulation in rabbits [40]. These results confirm that LDs are considered a source of energy for oocyte maturation [41, 42].

SCD catalyzes the rate-limiting step in the production of monounsaturated fatty acids, which are major substrates for the synthesis of triglycerides, wax esters, cholesterol esters, and phospholipids [56]. Recent research has revealed that alterations in SCD expression change the fatty acid profile of these lipids and have a significant role in lipid metabolism [43]. The integrated optical density of the LDs is related to the amount of triglycerides in the LDs. Our results showed that altered SCD expression affected LDs in GCs. The overexpression of SCD stimulated the accumulation of LD content, whereas SCD knockdown inhibited the accumulation of LD content (Fig. 2c). Previous research has demonstrated that SCD is an important control point in lipid homeostasis in goose GCs [23]. Similar results suggest that increased hepatic SCD activity accelerates the last stage of TG synthesis and stimulates lipid accumulation [44]. These results provide direct evidence that SCD expression plays a major role in the triacylglycerol and phospholipid secretion processes. Much evidence indicates that the direct anti-steatotic effect of SCD deficiency stems from increased fatty acid oxidation and reduced lipid synthesis [45] and that the inhibition of SCD decreases the proliferation and apoptosis of cells [46]. Additionally, a link between SCD activity and LD size was previously observed in the nematode *Caenorhabditis elegans* [47]; and experiments on cell lines cultured from patients with Berardinelli-Seip congenital lipodystrophy [48] demonstrated that SCD activity is necessary for large-sized LDs. Recent studies have indicated that the size of LDs might influence the balance of lipogenesis and lipolysis in adipocytes and hepatic cells [49], with decreased SCD expression resulting in reduced accumulation of oleic and palmitoleic acids and consequently fat cells with small LDs [50]. However, the functional implications of the diverse sizes of LDs in goose GCs are poorly understood.

In summary, these results provide new evidence that altered SCD expression changes GC lipid profiles, genes, and regulated relevant metabolic pathways, which might implicate SCD as one of the major genes in the regulation of lipid secretion by goose GCs.

## Methods

### Animals and granulosa layer isolation

Geese (from a maternal line of Tianfu goose) were raised under natural temperature and light conditions at the experimental station of waterfowl breeding at Sichuan Agricultural University. *Six* geese with regular laying sequences were randomly selected as experimental samples and sacrificed by cervical bleeding under anesthesia, 2 h after oviposition. A pool of ovarian follicles was immediately collected from the goose abdominal cavities and divided into prehierarchal follicles and hierarchal preovulatory follicles according to previously reported nomenclature [51]. For granulosa layer collection, the outer connective tissue was removed from the preovulatory follicles, and the preovulatory follicles were dissected to allow the yolk and adhered granulosa layer to flow out [52].

### Cell culture and transfection

The granulosa layer was dispersed by incubation in 0.1% type II collagenase (Sigma, USA) in Dulbecco’s modified Eagle’s medium (DMEM, HyClone, USA) for 10 min in a 37 °C water bath. After incubation, cells were dispersed with a pipette and pelleted by centrifugation at 1000×*g* for 10 min (20 °C). The supernatant was discarded and the cells were resuspended in 3 mL of fresh basic medium without collagenase and centrifuged. The washing procedure was repeated twice. The GCs were dispersed in DMEM supplemented with 1% antibiotic/antimycotic solution (Solarbio, China) and 3% fetal bovine serum (Gibco, USA). Transient transfections based on the GC cellular model of *SCD* function (*SCD*-specific overexpression and knockdown) were performed using Lipofectamine^®^ 3000 and Lipofectamine™ RNAiMAX Transfection Reagent (Invitrogen), according to manufacturer’s protocol. SCD siRNA-210 and siRNA-405 were used to achieve SCD mRNA knockdown, with scrambled siRNA as a negative control. The extent of SCD mRNA knockdown was measured as a percentage compared to that the scrambled siRNA. SCD-specific overexpression was used to achieve SCD mRNA overexpression, termed pEGFP-N1/SCD, and the pEGFP-N1/empty vector (GFP vector) served as the negative control, and another control with no transfection components was also included. The extent of SCD mRNA overexpression was measured as a percentage compared to that with the GFP vector and no transfection components conditions. Transfection efficiency was determined as previously described [23].

### Oil red O staining

To determine LD accumulation in GCs after transfection, the cells were stained with Oil Red O solution (Sigma Chemical Co., St. Louis, MO, USA). After 48 h of transfection, the cultured GCs were washed with phosphate-buffered saline (PBS) and fixed with 10% formaldehyde at room temperature. Then, the cells were washed twice with PBS, and were subsequently stained with 0.5 μg/mL Oil Red O solution in the dark for 1 h at room temperature. Then, cells were washed with PBS and photographed using an optical microscope system (Nikon ECLIPSE 90i) at 200 × magnification. Finally, the LDs were dissolved in isopropanol and the optical density (OD) value was determined at a wavelength of 540 nm using a microplate reader (Thermo Varioskan, USA). The relative lipid content was calculated using the following equation: relative LD content (%) = sample OD/mg × mL^−1^ protein.

### Immunofluorescence staining and confocal microscopy

For the photographic documentation of LD localization in goose GCs, live cells were stained with 4,4-difluoro-3a,4a-diaza-s-indacene (Bodipy 500/510, Thermo Fisher), wheat germ agglutinin (WGA, Alexa Fluor 594 conjugate, Invitrogen), and Hoechst dyes (33258, Invitrogen) for labeling the LDs, membrane, and nucleus, respectively. Briefly, cells on culture plates were rinsed with PBS and stained for 5 min with 2 μg/mL Bodipy 500/510. After staining, cells were washed three times with PBS for 5 min. The cells were then stained for 5 min with 5 μg/mL WGA and 5 μg/mL Hoechst 33,342. After staining and incubation, the cells were washed thrice with PBS for 5 min each. Finally, cells were fixed in formaldehyde for 10 min. The fixed cells were imaged using a confocal laser scanning microscope (FV1200, Olympus) mounted on an IX83 inverted microscope (Olympus). For treble-color images, a 550-nm laser line was used to image cells stained with Bodipy 500/510, a 635-nm laser line was used to image cells stained with WGA, and a 543-nm laser line was used to image cells stained with Hoechst 33258.

### Sample preparation and extraction

For lipidomic analysis, each group of cells (LS, LG, LN, and two independent siRNA groups [siRNA-210 and siRNA-470, referred to as LT and LF, respectively], as well as the LC) were placed in liquid nitrogen for 2 min, then thawed on ice for 5 min and vortexed. The cells were centrifuged at 12,000 rpm at 4 °C for 10 min. The supernatants (300 µL) were homogenized with a 1 mL mixture containing methanol, MTBE, and an internal standard. This mixture was stirred for 2 min, followed by the addition of 500 µL water, stirring for 1 min, and centrifugation at 12,000 rpm at 4 °C for 10 min. A total of 500 µL of the supernatant obtained was extracted and concentrated. The powder was dissolved with 100 µL mobile phase B (0.1% acetic acid), and the supernatant (200 μL) was transferred to an LC–MS sampling vial with an inner liner, for LC–MS analysis. Quality control (QC) samples were produced by pooling equal aliquots taken from each individual sample in the analytical run together. These QC samples were used to monitor the stability and reproducibility of the analytes in the samples during the analysis.

For RNA-sequencing analysis, each group of cells was extracted from three independent biological replicates using TRIzol reagent (Invitrogen, USA) according to the manufacturer’s instructions. RNA quality was confirmed on 1% agarose gels. RNA purity and concentration were determined using a NanoPhotometer^®^ spectrophotometer (IMPLEN, CA, USA) and Qubit^®^ RNA Assay Kit with a Qubit^®^ 2.0 Fluorometer (Life Technologies, CA, USA) according to the manufacturer’s instructions. RNA integrity was assessed using an RNA 6000 Nano Assay Kit (Bioanalyzer 2100 system; Agilent Technologies, CA, USA).

### Lipidomics profiling

Lipidomics profiling was performed by MetWare (Wuhan, China) using an LC–ESI–MS/MS system (UPLC, Shim-pack UFLC SHIMADZU CBM A system, https://www.shimadzu.com/; MS, QTRAP^®^ System, https://sciex.com/). LIT and triple quadrupole (QQQ) scans were acquired on a triple quadrupole-linear ion trap mass spectrometer (QTRAP^®^) LC–MS/MS System, equipped with an ESI Turbo Ion-Spray interface, operating in positive and negative ion mode, and controlled by Analyst 1.6.3 software (Sciex). Qualitative analysis of primary and secondary MS data was carried out by a comparison of the accurate precursor ions (Q1), product ions (Q3), retention time (RT), and fragmentation patterns with those obtained by injecting standards at the same conditions if the standards were available (Sigma-Aldrich, USA), or was conducted using the self-compiled database MWDB (MetWare, China). The quantitative analysis of metabolites was based on the MRM mode. The characteristic ions of each metabolite were screened using a QQQ mass spectrometer to obtain the signal strengths. Integration and correction of chromatographic peaks was performed using Progenesis QI software (Waters Co., Milford, MA, USA). The corresponding relative metabolite contents were represented as chromatographic peak area integrals. In addition, accurate masses of features representing significant differences were searched against the METLIN, Kyoto Encyclopedia of Genes and Genomes (KEGG), HMDB, and LIPIDMAPS databases.

### RNA-sequencing

Sequencing libraries were generated by Novogene (Beijing, China) using a NEBNext^®^ Ultra™ RNA Library Prep Kit (Illumina^®^, NEB, USA) following the manufacturer’s instructions. Clustering of the index-coded samples was performed on a cBot Cluster Generation System by using a TruSeq PE Cluster Kit v3-cBot-HS (Illumina) according to the manufacturer’s instructions. After cluster generation, the library preparations were sequenced on an Illumina HiSeq (Illumina, USA) platform and 125 bp/150 bp paired-end reads were generated.

### Bioinformatic and statistical analyses of data

Raw LC–MS data were filtered, identified, integrated, corrected, aligned, and normalized using Progenesis QI software (Waters Co., Milford, MA, USA). A data matrix of RT, mass-to-charge ratio, and peak intensity was obtained. The processed data were analyzed using principal component analysis and orthogonal correction partial least squares discriminant analysis (PC) using SIMCA-P14.0 software (Umetrics, Umeå, Sweden). Differentially abundant metabolites between dietary treatments were identified from variable importance in projection (VIP) from OPLS-DA and Student’s t tests (VIP > 1 and *P* < 0.05). Metabolites were identified from public databases, including MassBank (http://www.massbank.jp/), KNApSAcK (http://kanaya.naist.jp/KNApSAcK/), Human Metabolome Database (http://www.hmdb.ca/), and METLIN (https://metlin.scripps.edu/). The KEGG database (http://www.genome.jp/kegg/) was used to view the enriched pathways of the different metabolites. Hierarchical clustering analysis and heat map analysis were conducted using the R package, version 3.3.1. The raw read counts were normalized considering both the different depths of sequencing among the samples and the gene GC content. Normalization was performed using the EDASeq package. We considered all DEGs with a *P* value < 0.05 after false discovery rate correction. The putative functions of DEGs were investigated by Gene Ontology (GO) enrichment analysis using the R package GOseq [53], in which gene length bias was corrected. GO terms with a corrected *P* value < 0.05 were considered significantly enriched. KOBAS software was used to test the statistical enrichment of DEGs in KEGG pathways [54]. Statistical plots were prepared using Origin version 6.1.

## Supplementary Information

**Additional file 1**: **Figure S1**. Unsupervised hierarchical clustering of all lipids in each group; each column denotes one group. Increased lipids concentrations are shown in red, whereas decreased lipids concentrations are shown in blue.

**Additional file 2**: **Figure S2**. Venn diagram of overlapping and unique of lipids altered in each group.

**Additional file 3**: **Figure S3**. Unsupervised hierarchical clustering of all DEGs in each group; each column denotes one group. Increased DEGs concentrations are shown in red, whereas decreased DEGs concentrations are shown in blue.

**Additional file 4**: **Figure S4**. Topology analysis of top 20 pathways identified of transcriptomics. (a) Overexpressed group, NC vs. OS comparison and OG vs. OS comparison; (b) Knockdown group, SC vs. ST comparison and SC vs. SF comparison. Advanced bubble chart shows the enrichment of differentially abundant DEGs in pathways. The x-axis represents the rich factor (rich factor = number of different DEGs enriched in the pathway/number of all DEGs in the background DEGs set). The y-axis represents represent the enriched pathways. Size of the bubble represent the number of different abundant DEGs enriched in the pathway, and the color represents enrichment significance.

**Additional file 5**: **Figure S5**. Visualization of the CCA results of lipids and DEGs involved in (a) inositol phosphate metabolism, (b) arachidonic acid metabolism, (c) sphingolipid metabolism, (d) phosphatidylinositol signaling system and (e) glycine, serine and threonine metabolism under SCD knockdown.

**Additional file 6: Table S1**. Significant differential lipids involved in LN vs. LS comparison, LG vs. LS comparison, LC vs. LT comparison, and LC vs. LF comparison.

**Additional file 7: Table S2**. A functional analysis of pathways related to the differentially abundant lipids.

**Additional file 8: Table S3**. Basic characteristics of tags in each sample libraries and data of sequencing reads mapping to the reference genome.

**Additional file 9: Table S4**. The detail of DEGs were assigned with lipid-related GO slim terms.

**Additional file 10: Table S5**. The detail of lipids from glycerolipid/glycerophospholipid metabolism were subjected to a CCA together with transcript data of all those pathways as derived from SCD knockdown in GCs.

## Data Availability

RNA-seq data used in the current study are available at NCBI Bioproject database under the accession number PRJNA720652.

## Abbreviations

GCs: Granulosa cells
LDs: Intracellular lipid droplets
SCD: Stearoyl-CoA desaturase
CCA: Canonical correlation analysis
DEGs: Differentially expressed genes
TGs: Triacylglycerols
DMEM: Dulbecco’s modified Eagle’s medium
OD: Optical density
Bodipy: 4,4-Difluoro-3a,4a-diaza-*s*-indacene
WGA: Wheat germ agglutinin
QC: Quality control
KEGG: Kyoto Encyclopedia of Genes and Genomes
VIP: Importance in projection
GO: Gene ontology
