# Supplementary figures and images for: Lipidomics profiling of goose granulosa cell model of stearoyl-CoA desaturase function identifies a pattern of lipid droplets associated with follicle development
Source: Cell Biosci. 2021 May 22;11:95. doi: 10.1186/s13578-021-00604-6 (PMC8141238; doi:10.1186/s13578-021-00604-6)

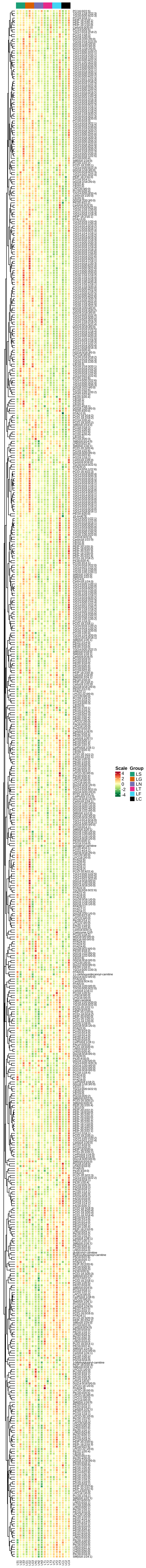

Supplement: Supplementary file 1 — Additional file 1: Figure S1. Unsupervised hierarchical clustering of all lipids in each group; each column denotes one group. Increased lipids concentrations are shown in red, whereas decreased lipids concentrations are shown in blue. [file 13578_2021_604_MOESM1_ESM.pdf]

## LC vs. LT

## LC vs. LF

## LN vs. LS

## LG vs. LS

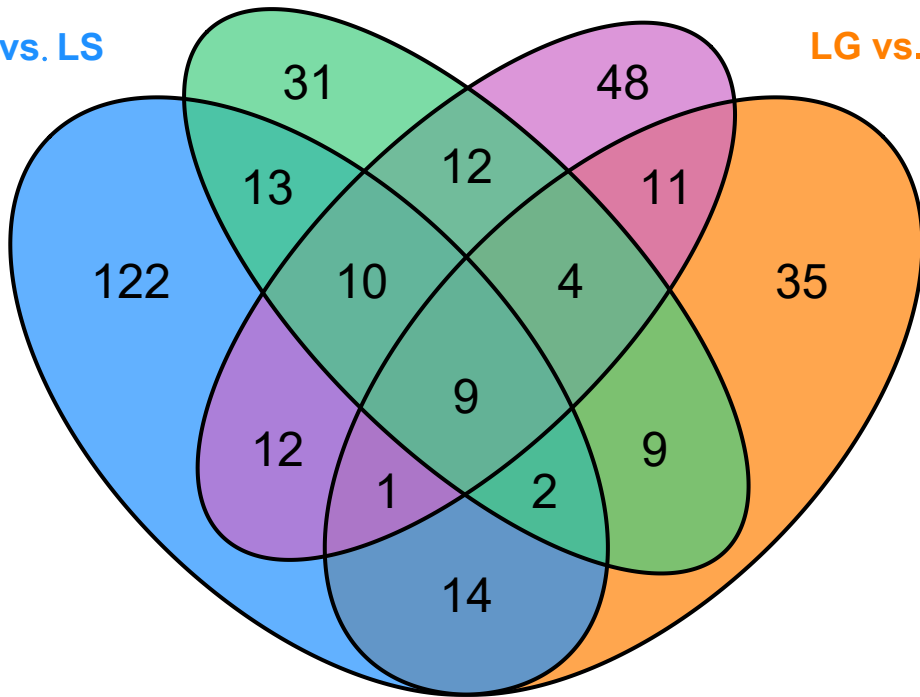

Supplement: Supplementary file 2 — Additional file 2: Figure S2. Venn diagram of overlapping and unique of lipids altered in each group. [file 13578_2021_604_MOESM2_ESM.pdf]

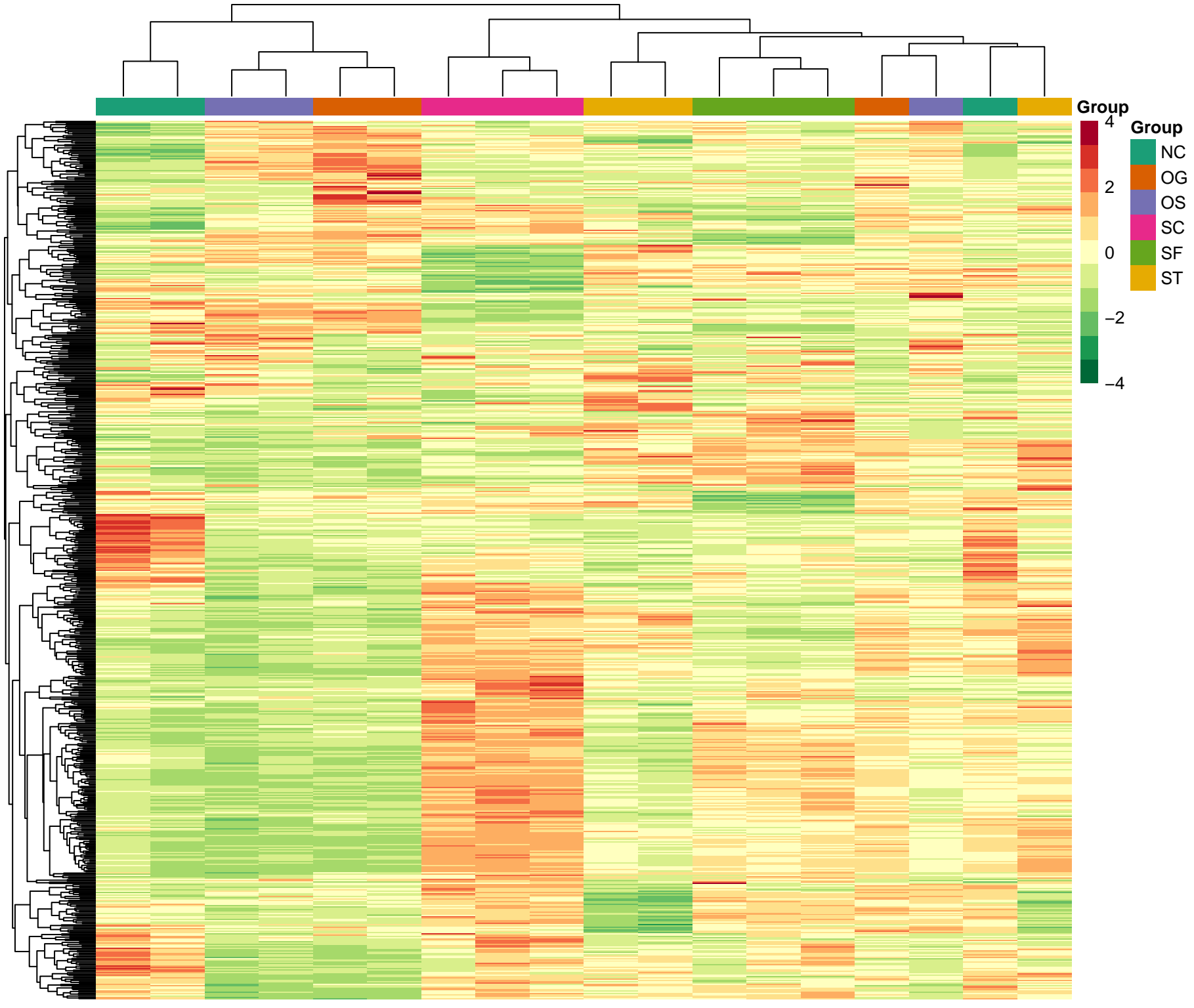

Supplement: Supplementary file 3 — Additional file 3: Figure S3. Unsupervised hierarchical clustering of all DEGs in each group; each column denotes one group. Increased DEGs concentrations are shown in red, whereas decreased DEGs concentrations are shown in blue. [file 13578_2021_604_MOESM3_ESM.pdf]

a

NC vs. OS

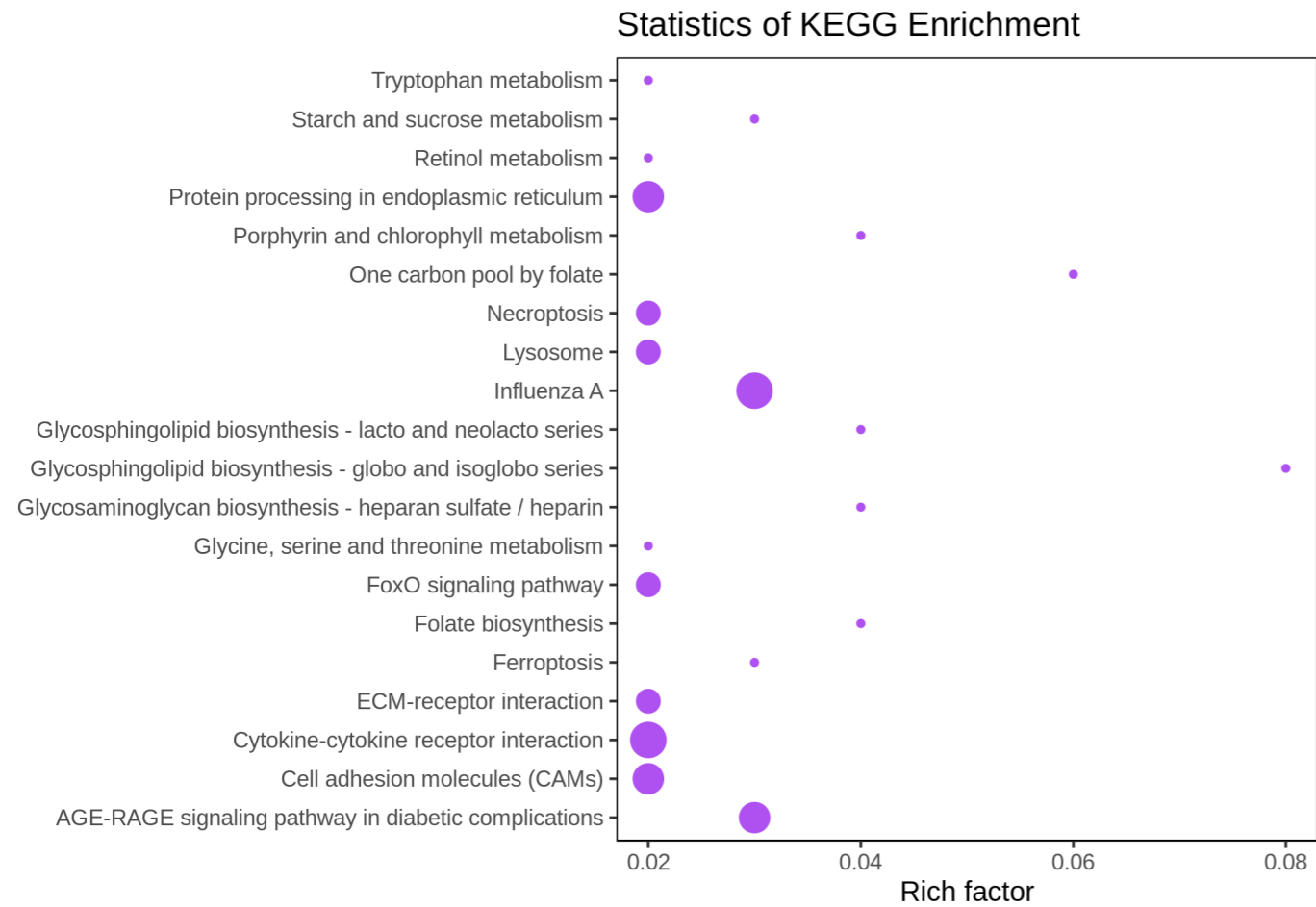

OG vs. OS

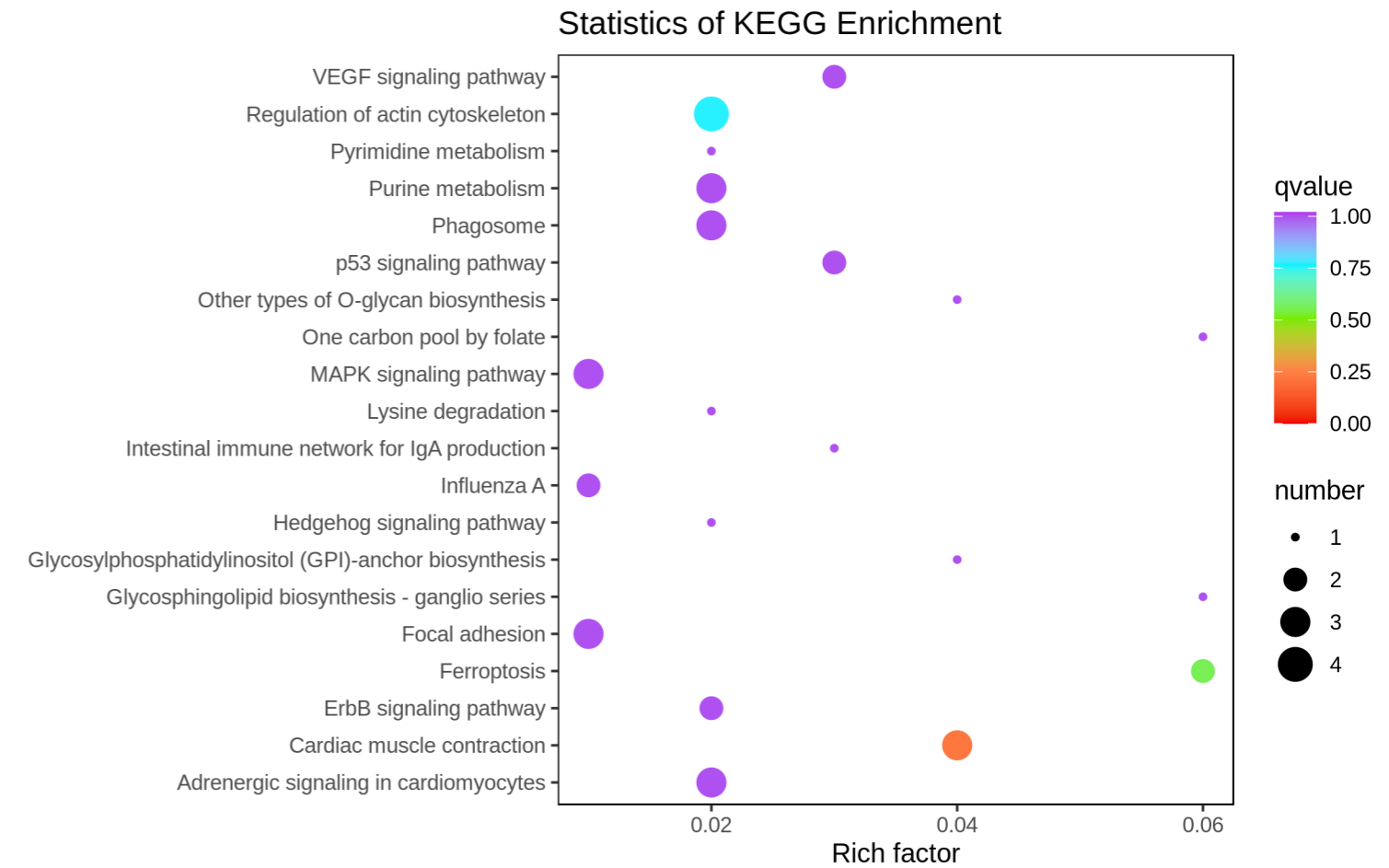

b

SC vs. ST

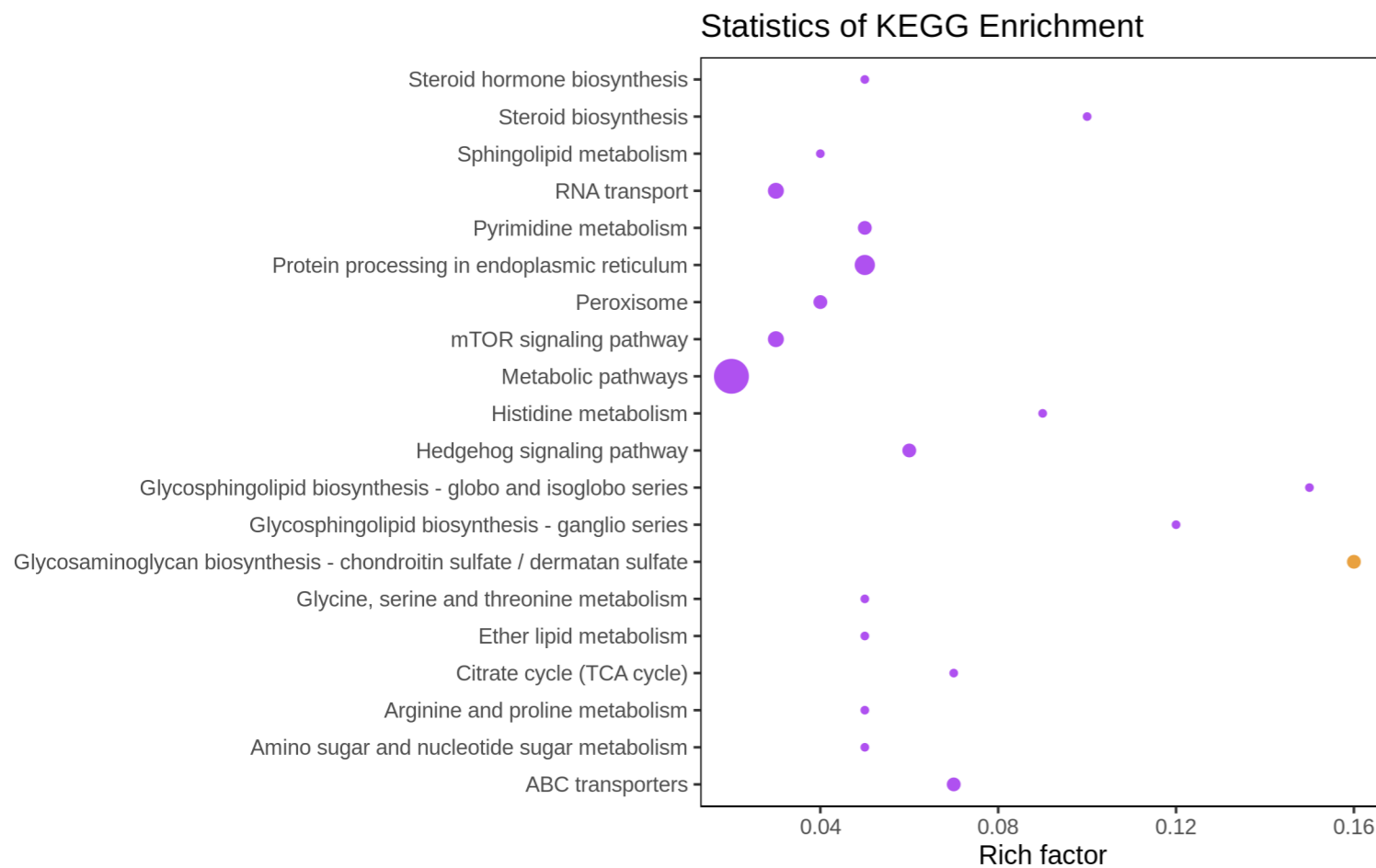

SC vs. SF

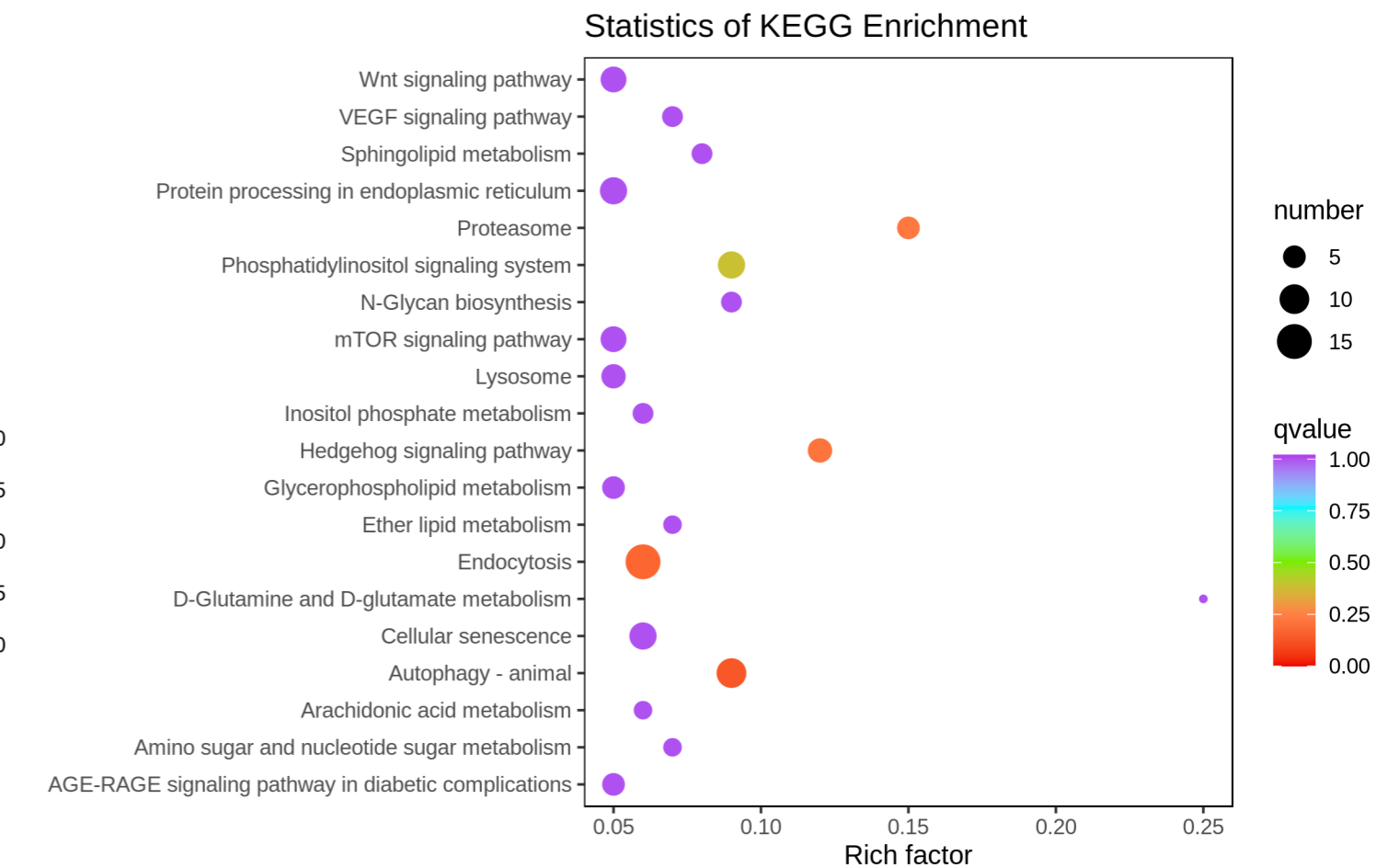

Supplement: Supplementary file 4 — Additional file 4: Figure S4. Topology analysis of top 20 pathways identified of transcriptomics. (a) Overexpressed group, NC vs. OS comparison and OG vs. OS comparison; (b) Knockdown group, SC vs. ST comparison and SC vs. SF comparison. Advanced bubble chart shows the enrichment of differentially abundant DEGs in pathways. The x-axis represents the rich factor (rich factor = number of different DEGs enriched in the pathway/number of all DEGs in the background DEGs set). The y-axis represents represent the enriched pathways. Size of the bubble represent the number of different abundant DEGs enriched in the pathway, and the color represents enrichment significance. [file 13578_2021_604_MOESM4_ESM.pdf]

a

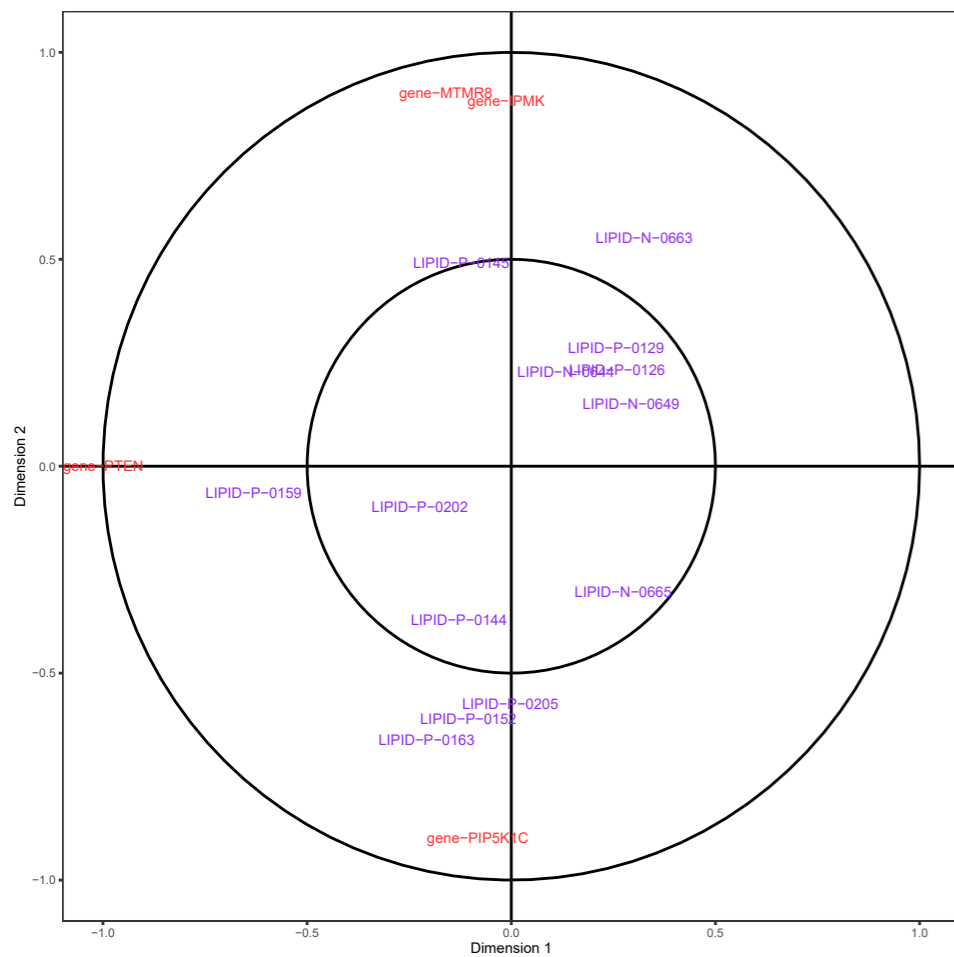

b

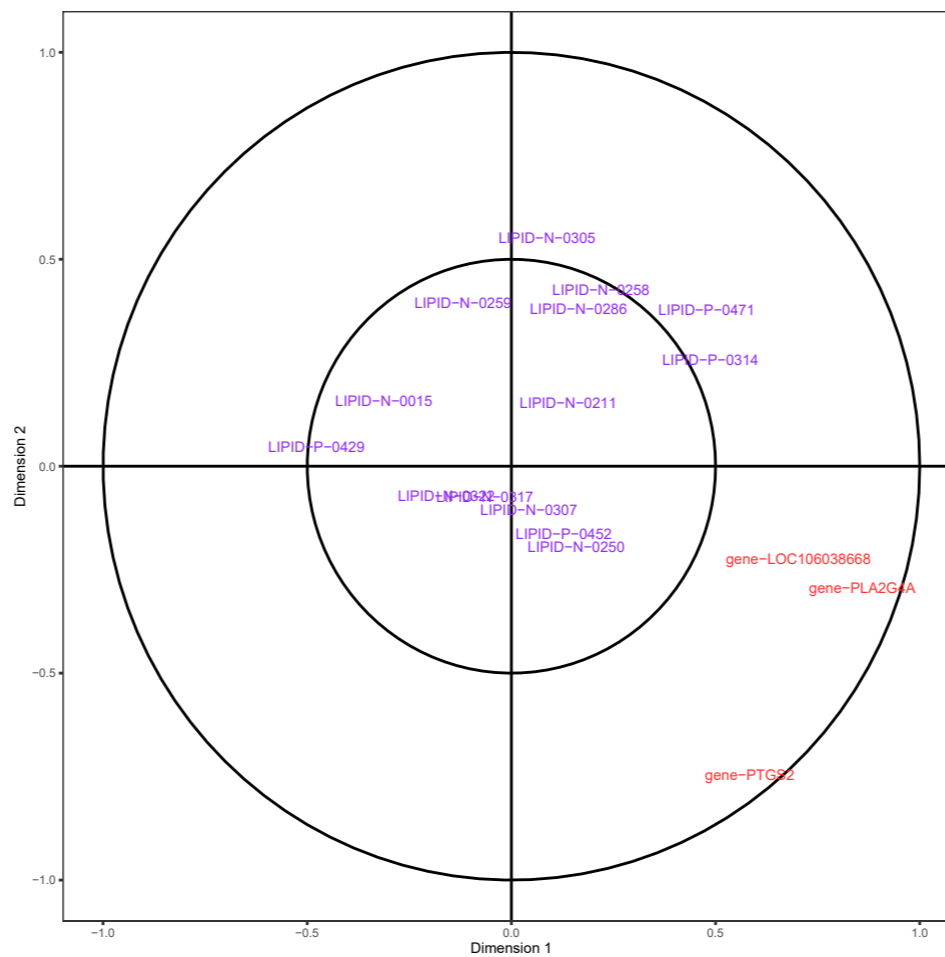

c

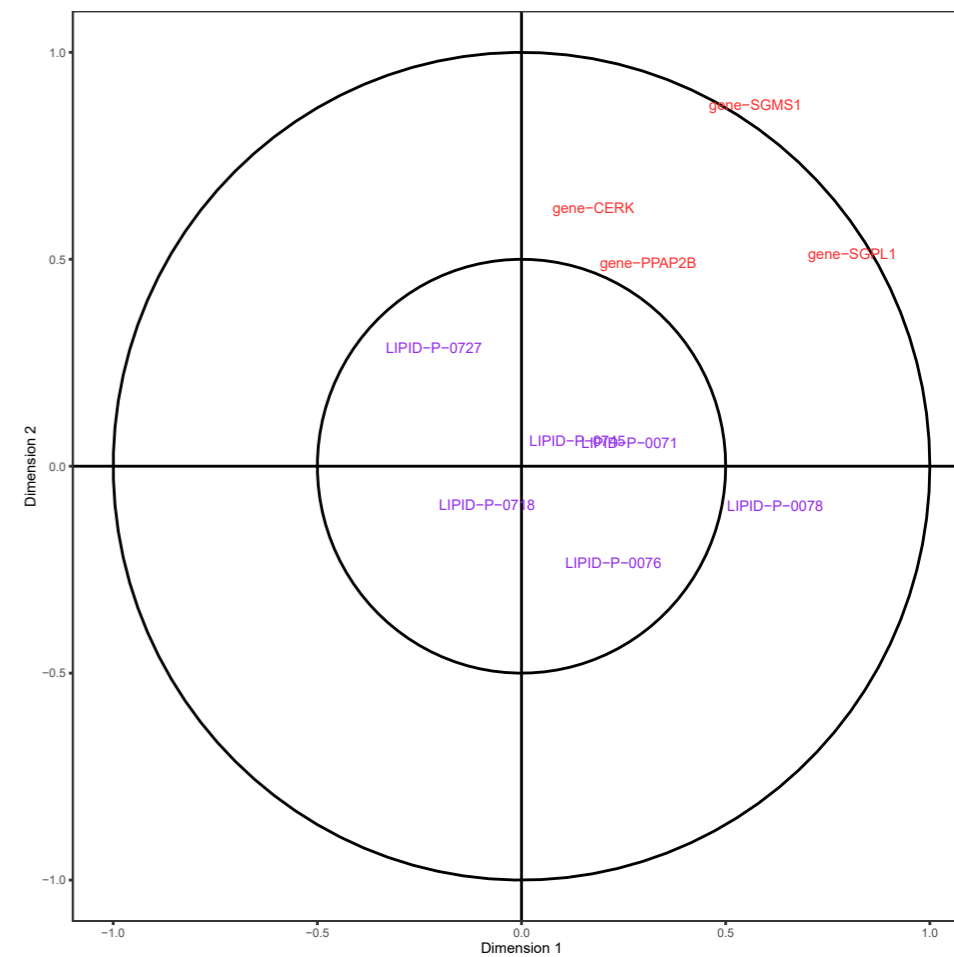

d

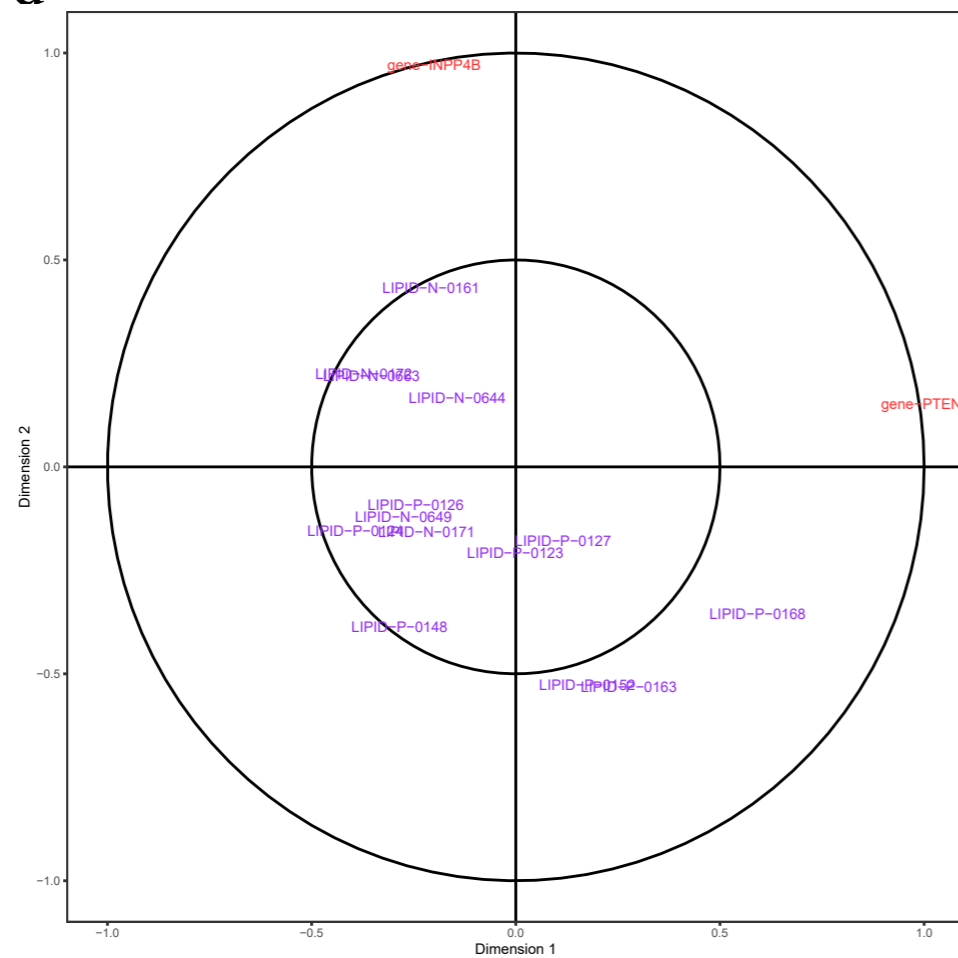

e

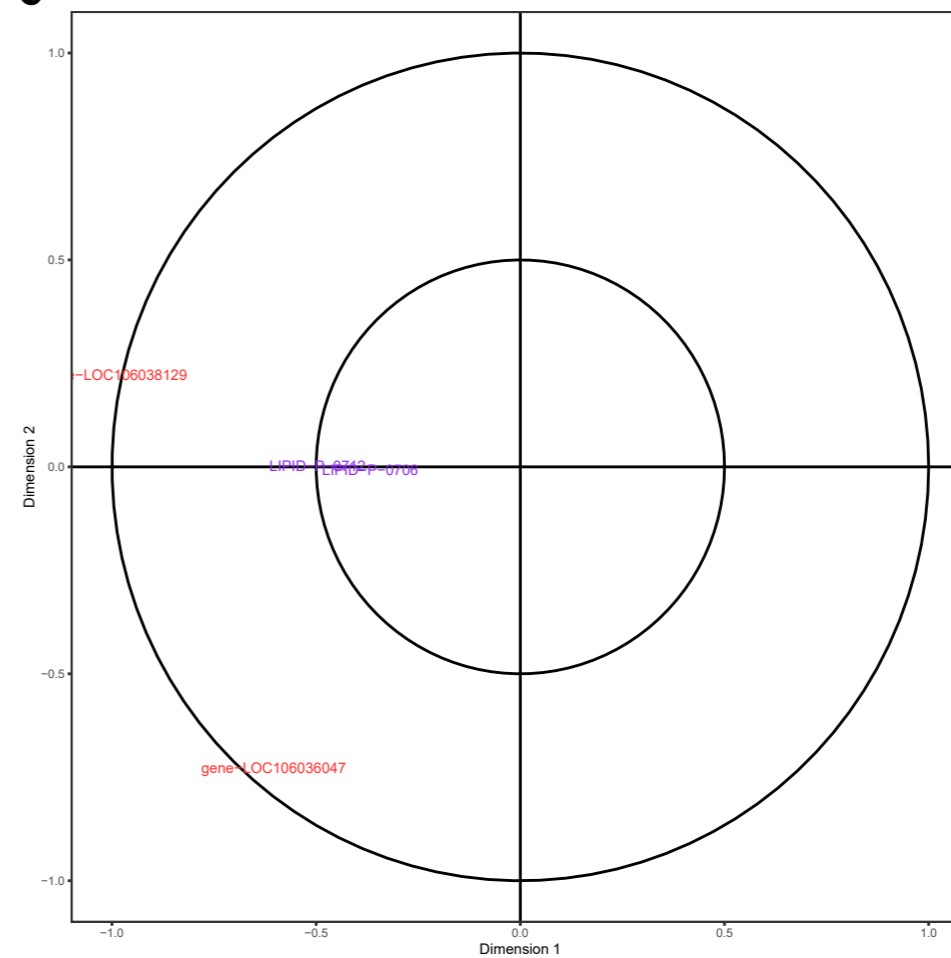

Supplement: Supplementary file 5 — Additional file 5: Figure S5. Visualization of the CCA results of lipids and DEGs involved in (a) inositol phosphate metabolism, (b) arachidonic acid metabolism, (c) sphingolipid metabolism, (d) phosphatidylinositol signaling system and (e) glycine, serine and threonine metabolism under SCD knockdown. [file 13578_2021_604_MOESM5_ESM.pdf]
