# Supplementary material for: Lipidomics profiling of goose granulosa cell model of stearoyl-CoA desaturase function identifies a pattern of lipid droplets associated with follicle development
Source: Cell Biosci. 2021 May 22;11:95. doi: 10.1186/s13578-021-00604-6 (PMC8141238; doi:10.1186/s13578-021-00604-6)
Supplement: Supplementary file 6 — Additional file 6: Table S1. Significant differential lipids involved in LN vs. LS comparison, LG vs. LS comparison, LC vs. LT comparison, and LC vs. LF comparison. [file 13578_2021_604_MOESM6_ESM.docx]

| **Supplementary Table 1. Significant differential lipids involved in LN vs. LS comparison, LG vs. LS comparison, LC vs. LT comparison, and LC vs. LF comparison.** | | | | |
| --- | --- | --- | --- | --- |
| experimental vs. control | Compound | Class | VIP | Fold Change |
| LN vs. LS comparison | FFA(20:4) | Eicosanoid | 1.605552 | 1.274449 |
|  | FFA(13:0) | FFA | 1.49072 | 1.248829 |
|  | FFA(22:0) | FFA | 1.644076 | 1.205054 |
|  | FFA(16:1) | FFA | 1.125014 | 1.202994 |
|  | FFA(17:1) | FFA | 1.200155 | 1.29348 |
|  | FFA(24:1) | FFA | 2.015226 | 1.260601 |
|  | FFA(18:2) | FFA | 1.726547 | 1.332436 |
|  | FFA(20:2) | FFA | 1.623761 | 1.330075 |
|  | FFA(22:2) | FFA | 1.641993 | 1.80958 |
|  | FFA(24:6) | FFA | 1.165461 | 0.706728 |
|  | LPC(18:2/0:0) | LPC | 1.340874 | 1.251455 |
|  | LPC(20:3/0:0) | LPC | 1.181447 | 1.220826 |
|  | LPE(0:0/18:1) | LPE | 1.072993 | 1.281058 |
|  | PA(18:0/18:1) | PA | 1.485607 | 1.245814 |
|  | PA(20:1/18:0) | PA | 1.335383 | 1.223544 |
|  | PC(18:0/14:1) | PC | 1.562161 | 0.826417 |
|  | PC(14:0/18:2) | PC | 1.046012 | 1.298319 |
|  | PC(14:1/18:2) | PC | 1.958731 | 1.235795 |
|  | PC(16:0/18:3) | PC | 1.066501 | 1.286848 |
|  | PC(16:0/20:3) | PC | 2.102996 | 0.678469 |
|  | PC(16:1/18:3) | PC | 1.02509 | 1.441595 |
|  | PC(18:1/18:4) | PC | 1.964749 | 0.612302 |
|  | PC(20:5/18:0) | PC | 1.253116 | 1.283412 |
|  | PC(18:2/20:3) | PC | 1.463847 | 0.637609 |
|  | PE(22:0/18:1) | PE | 1.748041 | 1.289421 |
|  | PE(18:1/18:1) | PE | 1.911409 | 1.883456 |
|  | PE(20:1/16:1) | PE | 1.773524 | 1.934146 |
|  | PE(22:6/18:0) | PE | 1.261061 | 1.226149 |
|  | PG(18:2/18:1) | PG | 1.637026 | 1.459218 |
|  | PG(18:1/20:4) | PG | 1.945721 | 1.739537 |
|  | PI(18:1/20:4) | PI | 1.778024 | 0.809124 |
|  | 3-Methylglutaryl-carnitine | CAR | 1.681594 | 1.307307 |
|  | 11-carboxyundecanoyl-carnitine | CAR | 1.050817 | 0.805095 |
|  | Tiglyl-carnitine | CAR | 1.529871 | 1.979833 |
|  | CE(14:0) | CE | 1.123964 | 1.341713 |
|  | CE(18:2) | CE | 1.182374 | 0.823643 |
|  | CE(20:4) | CE | 1.423266 | 1.522884 |
|  | CE(22:5) | CE | 1.193469 | 1.205264 |
|  | CE(22:6) | CE | 2.088389 | 0.548116 |
|  | Cer(d18:0/20:0) | Cer | 1.423925 | 1.308636 |
|  | Cer(d18:0/22:0) | Cer | 2.080635 | 1.393019 |
|  | Cer(m18:1/24:0) | Cerm | 1.651679 | 1.379281 |
|  | Cer(t18:0/20:2) | Cert | 1.801298 | 1.310626 |
|  | DG(16:0/20:0/0:0) | DG | 1.427562 | 1.238181 |
|  | DG(14:0/18:1/0:0) | DG | 1.35156 | 1.201373 |
|  | DG(16:1/18:0/0:0) | DG | 1.051669 | 1.218171 |
|  | DG(16:1/16:1/0:0) | DG | 1.575978 | 1.387942 |
|  | DG(14:0/18:2/0:0) | DG | 1.736324 | 1.377707 |
|  | DG(18:2/22:4/0:0) | DG | 1.380292 | 0.789322 |
|  | DG(18:1/22:5/0:0) | DG | 1.785407 | 0.697061 |
|  | LPC(16:1/0:0) | LPC | 1.000135 | 1.21758 |
|  | LPC(18:3/0:0) | LPC | 1.209652 | 1.520688 |
|  | LPC(22:4/0:0) | LPC | 2.083644 | 1.587864 |
|  | LPC(22:6/0:0) | LPC | 1.304506 | 1.201752 |
|  | LPE(0:0/16:0) | LPE | 1.060752 | 1.221311 |
|  | LPE(0:0/18:0) | LPE | 1.379501 | 1.248668 |
|  | LPE(0:0/20:3) | LPE | 1.233912 | 1.415679 |
|  | LPE(0:0/22:4) | LPE | 1.992003 | 1.366562 |
|  | LPE(0:0/22:6) | LPE | 1.327054 | 1.245636 |
|  | PC(18:0/22:0) | PC | 1.653021 | 1.26251 |
|  | PC(14:0/18:1) | PC | 1.046228 | 1.22668 |
|  | PC(18:0/18:2) | PC | 1.108698 | 1.210791 |
|  | PC(18:3/14:1) | PC | 1.614438 | 1.273879 |
|  | PC(20:5/12:0) | PC | 1.623401 | 1.389217 |
|  | PC(18:1/20:4) | PC | 1.896546 | 1.359455 |
|  | PC(18:2/20:4) | PC | 1.379299 | 0.672469 |
|  | PC(18:0/22:6) | PC | 1.064271 | 1.241873 |
|  | PC(20:4/20:4) | PC | 1.164553 | 0.777793 |
|  | PC(22:2/22:6) | PC | 1.635959 | 0.819306 |
|  | PC(O-18:0/18:2) | PC-O | 1.256819 | 1.219 |
|  | PC(O-16:2/18:1) | PC-O | 1.527547 | 1.252555 |
|  | PC(O-18:2/20:2) | PC-O | 1.28031 | 1.52915 |
|  | PC(O-18:3/20:4) | PC-O | 1.081475 | 0.812647 |
|  | PC(O-20:4/22:3) | PC-O | 1.528688 | 1.281299 |
|  | PE(22:0/18:0) | PE | 2.205891 | 1.434874 |
|  | PE(24:0/18:1) | PE | 1.921582 | 1.642776 |
|  | PE(18:2/14:0) | PE | 1.285418 | 1.298038 |
|  | PE(22:2/12:0) | PE | 1.373985 | 0.545413 |
|  | PE(22:2/14:0) | PE | 1.704046 | 1.552254 |
|  | PE(18:0/18:2) | PE | 1.588533 | 1.332525 |
|  | PE(22:1/20:1) | PE | 1.17528 | 1.212562 |
|  | PE(18:2/14:1) | PE | 1.514844 | 1.430428 |
|  | PE(18:4/20:0) | PE | 1.464949 | 1.311186 |
|  | PE(20:1/20:5) | PE | 1.522658 | 1.86784 |
|  | PE(P-18:0/20:0) | PE-P | 1.084809 | 1.230285 |
|  | PE(P-20:0/20:0) | PE-P | 1.526215 | 1.315311 |
|  | PE(P-18:2/18:0) | PE-P | 1.542961 | 1.374742 |
|  | PE(P-18:2/18:3) | PE-P | 1.112418 | 1.206316 |
|  | PE(P-18:2/20:4) | PE-P | 1.689665 | 1.239474 |
|  | PG(18:0/16:0) | PG | 1.043299 | 1.277111 |
|  | PS(18:0/16:1) | PS | 1.553204 | 1.362179 |
|  | PS(20:4/20:0) | PS | 1.039502 | 1.231442 |
|  | PS(20:5/18:0) | PS | 1.358471 | 1.39868 |
|  | PS(18:0/22:6) | PS | 1.454752 | 1.243842 |
|  | SM(d18:0/14:0) | SM | 1.425145 | 1.309997 |
|  | SM(d18:0/18:0) | SM | 1.12926 | 0.704539 |
|  | SM(d18:0/22:0) | SM | 1.098984 | 1.315133 |
|  | SM(d18:0/24:0) | SM | 1.541361 | 1.247332 |
|  | SM(d18:0/18:1) | SM | 1.51955 | 1.422236 |
|  | SM(d18:1/20:1) | SM | 1.761955 | 1.45755 |
|  | TG(14:0/16:0/16:0) | TG | 1.836591 | 1.413477 |
|  | TG(14:0/16:0/18:0) | TG | 1.572126 | 1.26334 |
|  | TG(16:0/16:0/18:0) | TG | 1.717051 | 1.457292 |
|  | TG(14:0/18:0/18:0) | TG | 1.528164 | 1.409267 |
|  | TG(14:0/16:0/22:0) | TG | 1.05093 | 1.496721 |
|  | TG(14:0/18:0/20:0) | TG | 1.174099 | 1.202264 |
|  | TG(12:0/16:0/16:1) | TG | 2.147511 | 1.512187 |
|  | TG(12:0/14:0/18:1) | TG | 1.747668 | 1.671414 |
|  | TG(14:0/14:0/18:1) | TG | 1.756513 | 1.250415 |
|  | TG(14:0/16:0/16:1) | TG | 1.697583 | 1.207353 |
|  | TG(14:0/14:0/20:1) | TG | 1.796854 | 1.304574 |
|  | TG(14:0/16:0/18:1) | TG | 1.890986 | 1.370466 |
|  | TG(16:0/16:0/16:1) | TG | 1.757187 | 1.264583 |
|  | TG(14:0/18:0/18:1) | TG | 1.208374 | 1.237805 |
|  | TG(14:0/18:1/20:0) | TG | 1.139293 | 1.251049 |
|  | TG(14:0/18:0/20:1) | TG | 1.266984 | 1.270745 |
|  | TG(14:0/20:0/20:1) | TG | 1.083969 | 1.25306 |
|  | TG(14:1/16:0/16:1) | TG | 1.770796 | 1.26395 |
|  | TG(14:0/14:1/18:1) | TG | 1.773001 | 1.416694 |
|  | TG(14:0/14:0/18:2) | TG | 1.958798 | 1.495419 |
|  | TG(16:0/16:1/16:1) | TG | 1.948961 | 1.285872 |
|  | TG(14:0/16:1/18:1) | TG | 1.623403 | 1.235297 |
|  | TG(14:0/16:0/18:2) | TG | 1.180893 | 1.250299 |
|  | TG(14:0/18:0/18:2) | TG | 1.727592 | 1.383052 |
|  | TG(14:0/20:1/20:1) | TG | 1.529761 | 1.464706 |
|  | TG(16:1/18:1/20:0) | TG | 1.053732 | 1.264452 |
|  | TG(14:0/18:2/22:0) | TG | 1.153864 | 1.445139 |
|  | TG(14:0/20:0/20:2) | TG | 1.143312 | 1.394955 |
|  | TG(14:0/22:0/22:2) | TG | 1.936204 | 2.120402 |
|  | TG(14:1/14:1/18:1) | TG | 1.907214 | 1.495316 |
|  | TG(14:0/14:1/18:2) | TG | 1.934504 | 1.656025 |
|  | TG(12:0/16:0/18:3) | TG | 1.667365 | 1.515177 |
|  | TG(12:0/18:0/18:3) | TG | 1.49145 | 1.248696 |
|  | TG(14:1/16:1/18:1) | TG | 1.452214 | 1.525629 |
|  | TG(14:0/18:1/18:2) | TG | 1.688644 | 1.370435 |
|  | TG(14:0/18:0/18:3) | TG | 1.730496 | 1.373141 |
|  | TG(14:0/18:1/20:2) | TG | 1.271499 | 1.27626 |
|  | TG(16:0/16:1/20:2) | TG | 1.356878 | 1.290855 |
|  | TG(14:0/18:0/20:3) | TG | 2.096994 | 1.618301 |
|  | TG(16:0/16:0/22:3) | TG | 1.129868 | 1.367998 |
|  | TG(18:0/18:1/18:2) | TG | 1.019366 | 1.317959 |
|  | TG(14:0/22:1/22:2) | TG | 1.199126 | 1.569325 |
|  | TG(12:0/18:1/18:3) | TG | 1.729963 | 1.422422 |
|  | TG(14:0/16:1/18:3) | TG | 1.313317 | 1.218549 |
|  | TG(14:1/14:1/22:2) | TG | 1.247647 | 1.470773 |
|  | TG(16:0/16:1/18:3) | TG | 1.633403 | 1.253668 |
|  | TG(14:0/18:1/18:3) | TG | 1.381766 | 1.582644 |
|  | TG(14:0/18:1/20:3) | TG | 1.487953 | 1.241248 |
|  | TG(14:0/18:2/20:2) | TG | 1.064798 | 1.369805 |
|  | TG(14:0/16:0/22:4) | TG | 1.786838 | 1.650735 |
|  | TG(14:0/18:0/20:4) | TG | 1.594203 | 1.458799 |
|  | TG(14:0/20:2/20:2) | TG | 1.429715 | 1.330194 |
|  | TG(16:0/16:1/22:3) | TG | 1.442609 | 1.274255 |
|  | TG(14:0/18:3/22:1) | TG | 1.504311 | 1.637811 |
|  | TG(14:0/18:0/22:4) | TG | 1.59872 | 1.404419 |
|  | TG(18:1/18:3/20:0) | TG | 1.491053 | 1.574771 |
|  | TG(12:0/22:1/22:3) | TG | 1.203228 | 1.514826 |
|  | TG(18:0/18:3/20:1) | TG | 1.565994 | 1.561523 |
|  | TG(14:0/20:3/22:1) | TG | 1.180118 | 1.417833 |
|  | TG(14:0/18:2/18:3) | TG | 2.089456 | 1.60356 |
|  | TG(14:0/18:1/18:4) | TG | 1.556329 | 1.630557 |
|  | TG(14:0/16:1/20:4) | TG | 1.149843 | 1.349184 |
|  | TG(16:0/16:1/18:4) | TG | 1.869768 | 1.505473 |
|  | TG(14:0/16:0/22:5) | TG | 2.018646 | 1.65451 |
|  | TG(14:0/18:4/20:1) | TG | 1.375669 | 1.513673 |
|  | TG(14:0/18:1/20:4) | TG | 1.654885 | 1.298567 |
|  | TG(18:0/18:2/18:3) | TG | 1.206586 | 1.247432 |
|  | TG(16:0/16:1/22:4) | TG | 1.145716 | 1.269834 |
|  | TG(14:0/20:1/20:4) | TG | 1.709213 | 1.371784 |
|  | TG(16:0/20:4/22:1) | TG | 1.103004 | 1.512867 |
|  | TG(14:0/18:3/22:3) | TG | 1.061391 | 1.79566 |
|  | TG(14:0/20:4/22:2) | TG | 1.203067 | 1.234115 |
|  | TG(14:0/18:3/20:4) | TG | 1.139616 | 1.380324 |
|  | TG(14:0/18:2/20:5) | TG | 1.050842 | 1.587478 |
|  | TG(14:0/20:3/20:4) | TG | 1.236672 | 1.504443 |
|  | TG(14:0/20:2/20:5) | TG | 1.35049 | 1.377288 |
|  | TG(16:0/16:1/22:6) | TG | 1.454267 | 1.285574 |
|  | TG(16:0/20:4/22:3) | TG | 1.308442 | 1.300798 |
|  | TG(14:0/20:4/20:4) | TG | 1.72168 | 1.437858 |
|  | TG(14:0/18:2/22:6) | TG | 1.711486 | 1.218924 |
|  | TG(16:1/20:4/20:4) | TG | 1.582417 | 1.566603 |
|  | TG(14:0/20:3/22:6) | TG | 1.638706 | 1.491558 |
|  | TG(14:0/22:3/22:6) | TG | 1.008294 | 0.82462 |
| LG vs. LS comparison | FFA(4:0) | FFA | 1.499189 | 0.824869 |
|  | FFA(6:0) | FFA | 2.072488 | 1.589536 |
|  | FFA(17:0) | FFA | 1.842253 | 1.31021 |
|  | FFA(18:0) | FFA | 1.373435 | 1.212499 |
|  | FFA(20:0) | FFA | 2.887065 | 1.233259 |
|  | FFA(22:2) | FFA | 2.380738 | 1.538628 |
|  | LPI(14:1/0:0) | LPI | 1.286456 | 1.397076 |
|  | PC(18:0/12:0) | PC | 1.397362 | 1.226541 |
|  | PC(18:0/14:0) | PC | 1.75498 | 1.371999 |
|  | PC(18:0/14:1) | PC | 1.214207 | 0.830418 |
|  | PC(16:0/20:3) | PC | 2.833873 | 0.711853 |
|  | PC(18:1/18:4) | PC | 2.066214 | 0.771515 |
|  | PC(20:5/18:0) | PC | 1.804772 | 1.290282 |
|  | PC(18:2/20:3) | PC | 1.117955 | 1.260047 |
|  | PE(16:1/22:0) | PE | 1.489594 | 1.324029 |
|  | PE(18:3/18:1) | PE | 1.341101 | 1.259221 |
|  | PE(18:1/20:3) | PE | 1.76647 | 1.432723 |
|  | PE(16:0/20:5) | PE | 2.233295 | 1.767411 |
|  | PE(20:3/18:2) | PE | 1.084289 | 0.784247 |
|  | PE(20:3/20:4) | PE | 1.811979 | 1.225411 |
|  | PI(20:4/16:0) | PI | 2.206156 | 1.327053 |
|  | PI(18:0/20:5) | PI | 1.156371 | 1.231877 |
|  | Isovaleryl-carnitine | CAR | 1.185739 | 0.816957 |
|  | Hydroxyhexanoyl-carnitine | CAR | 2.525585 | 1.267561 |
|  | CE(20:2) | CE | 1.1311 | 1.339889 |
|  | CE(20:4) | CE | 1.706569 | 1.320492 |
|  | Cer(d18:0/16:0) | Cer | 1.463611 | 0.826462 |
|  | DG(16:0/20:1/0:0) | DG | 1.227858 | 0.786728 |
|  | DG(18:1/22:0/0:0) | DG | 1.774391 | 0.729664 |
|  | DG(16:0/18:3/0:0) | DG | 1.645653 | 0.800281 |
|  | DG(18:4/18:1/0:0) | DG | 2.372986 | 1.396917 |
|  | DG(18:2/22:4/0:0) | DG | 1.367566 | 1.271318 |
|  | LPC(18:3/0:0) | LPC | 1.036543 | 1.226578 |
|  | LPC(O-22:1/0:0) | LPC-O | 1.883882 | 0.808949 |
|  | LPE(0:0/22:5) | LPE | 1.633607 | 0.774603 |
|  | LPS(18:0/0:0) | LPS | 1.189665 | 1.528634 |
|  | PC(20:1/22:1) | PC | 2.595417 | 0.670925 |
|  | PC(22:4/18:1) | PC | 1.074237 | 1.219534 |
|  | PC(18:2/20:4) | PC | 1.455827 | 0.747236 |
|  | PC(20:0/22:6) | PC | 1.503036 | 0.741965 |
|  | PC(O-18:0/20:0) | PC-O | 1.455107 | 1.25405 |
|  | PC(O-18:2/20:2) | PC-O | 1.158737 | 1.238791 |
|  | PC(O-20:3/22:3) | PC-O | 1.337197 | 0.763964 |
|  | PC(O-18:3/18:4) | PC-O | 1.906021 | 0.812041 |
|  | PE(16:0/14:0) | PE | 1.353954 | 0.810522 |
|  | PE(20:0/18:0) | PE | 2.484455 | 0.800498 |
|  | PE(22:2/12:0) | PE | 2.006976 | 0.428087 |
|  | PE(22:2/14:0) | PE | 1.362373 | 1.204536 |
|  | PE(20:1/20:5) | PE | 2.324769 | 1.551889 |
|  | PE(P-20:1/20:0) | PE-P | 2.408538 | 0.814755 |
|  | PS(20:4/20:0) | PS | 2.23157 | 1.220062 |
|  | PS(20:5/18:0) | PS | 1.328643 | 1.231776 |
|  | SM(d18:0/16:0) | SM | 2.996431 | 0.641709 |
|  | SM(d18:0/18:0) | SM | 1.138818 | 0.713912 |
|  | SM(d18:0/18:1) | SM | 2.200606 | 0.778999 |
|  | TG(14:0/18:0/18:0) | TG | 1.5184 | 0.824962 |
|  | TG(16:0/18:0/22:0) | TG | 1.480183 | 0.636592 |
|  | TG(16:0/20:0/22:0) | TG | 1.232749 | 0.283741 |
|  | TG(16:0/16:0/18:1) | TG | 1.352912 | 0.832772 |
|  | TG(14:0/22:0/22:2) | TG | 2.260243 | 1.332373 |
|  | TG(14:0/20:0/22:3) | TG | 1.43806 | 0.797988 |
|  | TG(14:0/22:1/22:2) | TG | 2.131952 | 1.481943 |
|  | TG(16:1/20:2/22:0) | TG | 1.481837 | 0.788997 |
|  | TG(12:0/18:1/18:3) | TG | 1.949568 | 0.789446 |
|  | TG(14:0/20:1/20:3) | TG | 2.295526 | 0.780881 |
|  | TG(14:0/20:4/22:0) | TG | 1.292606 | 0.831626 |
|  | TG(18:1/20:1/20:2) | TG | 1.757668 | 1.441695 |
|  | TG(14:0/18:2/18:3) | TG | 1.576277 | 0.757267 |
|  | TG(16:0/16:1/18:4) | TG | 1.60825 | 0.814648 |
|  | TG(16:0/16:1/20:4) | TG | 2.719685 | 0.73372 |
|  | TG(14:0/18:4/20:1) | TG | 2.092533 | 0.642413 |
|  | TG(16:0/16:1/20:5) | TG | 2.372024 | 0.809627 |
|  | TG(14:0/18:3/22:3) | TG | 1.078895 | 1.554629 |
|  | TG(16:0/16:1/22:5) | TG | 1.391794 | 0.815108 |
|  | TG(14:0/20:1/22:5) | TG | 1.220716 | 0.832261 |
|  | TG(18:0/18:3/20:3) | TG | 1.704424 | 0.791509 |
|  | TG(16:0/20:4/22:2) | TG | 1.275681 | 0.818955 |
|  | TG(16:1/16:1/20:5) | TG | 2.388481 | 0.704875 |
|  | TG(14:0/16:1/22:6) | TG | 1.610433 | 0.773311 |
|  | TG(18:2/18:3/20:2) | TG | 1.166814 | 1.229887 |
|  | TG(14:0/20:4/22:4) | TG | 1.217614 | 0.780469 |
|  | TG(18:2/18:3/20:3) | TG | 1.332712 | 0.739551 |
|  | TG(16:0/18:3/22:5) | TG | 1.375682 | 0.813766 |
|  | TG(14:0/22:4/22:4) | TG | 1.307537 | 0.71196 |
|  | TG(14:0/20:5/22:4) | TG | 1.930544 | 0.645807 |
| LC vs. LT comparison | 15-oxoETE | Eicosanoid | 1.490462 | 1.411355 |
|  | FFA(4:0) | FFA | 2.578001 | 0.629934 |
|  | FFA(6:0) | FFA | 1.726747 | 0.604889 |
|  | FFA(13:0) | FFA | 1.077402 | 1.503868 |
|  | FFA(14:0) | FFA | 1.918916 | 0.777696 |
|  | FFA(17:0) | FFA | 2.431763 | 0.775284 |
|  | FFA(18:0) | FFA | 2.260869 | 0.806486 |
|  | FFA(24:0) | FFA | 1.207838 | 1.409717 |
|  | FFA(22:1) | FFA | 1.609979 | 1.378167 |
|  | FFA(20:2) | FFA | 1.617088 | 1.503864 |
|  | FFA(22:2) | FFA | 2.434502 | 1.645353 |
|  | FFA(22:3) | FFA | 1.815013 | 1.260069 |
|  | FFA(24:6) | FFA | 2.054748 | 2.408304 |
|  | LPG(16:0/0:0) | LPG | 1.365937 | 0.658414 |
|  | LPI(14:1/0:0) | LPI | 1.359185 | 0.675382 |
|  | PA(16:0/18:0) | PA | 1.509223 | 0.831656 |
|  | PA(18:0/18:1) | PA | 1.996833 | 0.729854 |
|  | PA(16:0/20:1) | PA | 2.015403 | 0.796116 |
|  | PC(20:1/14:1) | PC | 1.259711 | 0.793116 |
|  | PC(16:0/20:3) | PC | 1.779246 | 0.631009 |
|  | PC(16:1/18:3) | PC | 1.543482 | 0.714234 |
|  | PC(18:2/18:2) | PC | 1.958079 | 1.284746 |
|  | PC(18:1/18:4) | PC | 2.585049 | 0.663502 |
|  | PC(18:2/20:3) | PC | 2.221244 | 1.625814 |
|  | PC(20:1/20:4) | PC | 1.371865 | 1.223128 |
|  | PC(18:1/22:6) | PC | 1.87292 | 1.216181 |
|  | PE(18:2/16:0) | PE | 1.712873 | 1.203551 |
|  | PE(20:2/16:0) | PE | 1.540616 | 0.790154 |
|  | PE(20:4/14:0) | PE | 1.732853 | 1.492326 |
|  | PE(20:5/18:1) | PE | 1.544584 | 0.687229 |
|  | PG(18:0/18:1) | PG | 1.897213 | 0.716591 |
|  | PG(18:1/18:1) | PG | 1.544701 | 0.779175 |
|  | PG(18:1/20:4) | PG | 1.239423 | 0.81013 |
|  | PI(20:3/18:0) | PI | 1.835669 | 0.634617 |
|  | PI(20:4/16:0) | PI | 1.771296 | 0.668144 |
|  | PI(18:1/20:4) | PI | 1.563095 | 0.765723 |
|  | Isovaleryl-carnitine | CAR | 1.344399 | 0.678787 |
|  | Hydroxyhexanoyl-carnitine | CAR | 2.318801 | 0.667748 |
|  | glutaconyl-carnitine | CAR | 2.681097 | 0.794997 |
|  | CE(14:0) | CE | 1.724178 | 0.745316 |
|  | CE(18:2) | CE | 1.90955 | 1.360868 |
|  | CE(20:4) | CE | 2.676318 | 1.475109 |
|  | CE(22:6) | CE | 2.031691 | 1.430911 |
|  | Cer(d18:1/22:0) | Cer | 1.86788 | 1.253678 |
|  | Cer(d18:1/24:0) | Cer | 2.347628 | 1.272671 |
|  | DG(12:0/22:0/0:0) | DG | 1.180686 | 0.790123 |
|  | DG(14:0/20:0/0:0) | DG | 1.897227 | 0.798005 |
|  | DG(16:0/18:0/0:0) | DG | 2.692423 | 0.747389 |
|  | DG(16:0/20:0/0:0) | DG | 2.44662 | 1.22931 |
|  | DG(16:1/22:0/0:0) | DG | 1.678203 | 1.272412 |
|  | DG(14:0/18:2/0:0) | DG | 1.415303 | 1.371116 |
|  | DG(20:0/18:2/0:0) | DG | 1.465931 | 1.321556 |
|  | DG(16:0/18:3/0:0) | DG | 1.382684 | 1.310291 |
|  | LPC(18:3/0:0) | LPC | 1.74507 | 1.614845 |
|  | LPE(0:0/18:0) | LPE | 2.021499 | 1.211575 |
|  | LPE(0:0/22:4) | LPE | 2.324881 | 1.202337 |
|  | PC(18:0/22:0) | PC | 1.820654 | 1.207826 |
|  | PC(22:2/18:1) | PC | 1.352036 | 0.823295 |
|  | PC(20:5/12:0) | PC | 1.822572 | 0.799346 |
|  | PC(18:2/20:4) | PC | 1.427528 | 1.327133 |
|  | PC(20:4/20:4) | PC | 1.366759 | 1.280828 |
|  | PC(O-20:4/22:3) | PC-O | 1.596917 | 0.731568 |
|  | PE(16:0/14:0) | PE | 2.354836 | 0.807712 |
|  | PE(24:0/18:1) | PE | 1.116217 | 1.21074 |
|  | PE(22:2/12:0) | PE | 2.46609 | 1.372431 |
|  | PE(22:1/20:3) | PE | 1.944252 | 0.763179 |
|  | PE(P-20:2/22:6) | PE-P | 2.666887 | 0.773014 |
|  | PS(20:4/20:0) | PS | 1.11512 | 0.792541 |
|  | PS(18:0/22:6) | PS | 2.089236 | 0.790408 |
|  | TG(14:0/16:0/22:0) | TG | 1.331224 | 1.308172 |
|  | TG(16:0/20:0/22:0) | TG | 1.574378 | 1.673906 |
|  | TG(14:0/14:1/18:1) | TG | 1.415755 | 1.227685 |
|  | TG(16:0/16:1/22:1) | TG | 1.403123 | 0.747448 |
|  | TG(14:0/22:0/22:2) | TG | 1.776684 | 0.768348 |
|  | TG(18:1/18:2/20:0) | TG | 1.92823 | 0.65188 |
|  | TG(14:0/20:1/22:2) | TG | 2.139248 | 0.620908 |
|  | TG(14:0/18:1/20:3) | TG | 2.332659 | 1.6468 |
|  | TG(14:0/20:1/20:3) | TG | 2.088351 | 0.665165 |
|  | TG(18:2/18:2/22:0) | TG | 1.068118 | 0.82886 |
|  | TG(18:2/18:3/20:0) | TG | 1.071448 | 1.243381 |
|  | TG(18:1/18:3/20:1) | TG | 1.361549 | 1.260972 |
|  | TG(16:1/18:1/18:4) | TG | 1.148655 | 1.206751 |
|  | TG(18:1/18:2/18:3) | TG | 1.877626 | 1.32929 |
|  | TG(14:0/18:3/22:3) | TG | 1.023438 | 1.412711 |
|  | TG(18:2/18:3/20:1) | TG | 1.474275 | 1.361063 |
|  | TG(14:0/20:1/22:5) | TG | 2.312415 | 1.250484 |
|  | TG(16:1/16:1/22:5) | TG | 1.234776 | 1.226757 |
|  | TG(14:0/20:3/22:4) | TG | 1.178093 | 0.65578 |
|  | TG(14:0/20:4/22:3) | TG | 1.127695 | 1.33175 |
|  | TG(16:0/20:4/22:6) | TG | 1.548917 | 0.692581 |
| LC vs. LF comparison | 15-oxoETE | Eicosanoid | 1.314636 | 1.515133 |
|  | FFA(4:0) | FFA | 2.198102 | 0.706752 |
|  | FFA(8:0) | FFA | 1.558192 | 0.6251 |
|  | FFA(12:0) | FFA | 1.250061 | 1.389417 |
|  | FFA(16:1) | FFA | 2.428752 | 1.263223 |
|  | FFA(17:1) | FFA | 1.94101 | 1.237563 |
|  | FFA(22:1) | FFA | 1.052215 | 1.220299 |
|  | FFA(20:2) | FFA | 1.383386 | 1.65623 |
|  | FFA(22:2) | FFA | 2.006439 | 1.65524 |
|  | FFA(22:3) | FFA | 1.972544 | 1.438157 |
|  | FFA(22:4) | FFA | 1.185815 | 1.202153 |
|  | FFA(24:4) | FFA | 1.773167 | 1.234527 |
|  | FFA(24:5) | FFA | 2.111013 | 1.221759 |
|  | FFA(24:6) | FFA | 1.749145 | 2.517775 |
|  | LPC(16:0/0:0) | LPC | 1.677456 | 1.260138 |
|  | LPC(18:2/0:0) | LPC | 2.186195 | 1.286081 |
|  | LPC(20:4/0:0) | LPC | 1.663023 | 1.354173 |
|  | LPE(0:0/20:0) | LPE | 2.187467 | 1.20682 |
|  | LPG(16:0/0:0) | LPG | 1.90831 | 1.72489 |
|  | PC(14:0/18:2) | PC | 1.050829 | 1.294787 |
|  | PC(16:0/20:3) | PC | 1.336145 | 0.630191 |
|  | PC(18:0/20:3) | PC | 1.748306 | 1.304307 |
|  | PC(20:1/18:2) | PC | 1.531638 | 1.261668 |
|  | PC(20:3/18:1) | PC | 1.713311 | 1.20393 |
|  | PC(16:0/20:5) | PC | 2.36374 | 1.309782 |
|  | PC(18:1/18:4) | PC | 1.482421 | 0.760726 |
|  | PC(18:2/20:3) | PC | 2.479472 | 1.9677 |
|  | PC(20:1/20:4) | PC | 1.190612 | 1.267595 |
|  | PE(18:0/14:0) | PE | 1.678528 | 1.408349 |
|  | PE(16:0/18:0) | PE | 1.68986 | 1.202902 |
|  | PE(16:1/16:0) | PE | 1.454208 | 1.202067 |
|  | PE(18:1/16:1) | PE | 1.596735 | 1.229799 |
|  | PE(18:2/16:0) | PE | 1.789894 | 1.347726 |
|  | PE(16:0/20:3) | PE | 1.977214 | 1.350207 |
|  | PE(20:4/14:0) | PE | 2.213672 | 1.65014 |
|  | PE(16:0/22:4) | PE | 1.476688 | 1.224618 |
|  | PE(16:0/20:5) | PE | 2.256447 | 1.236554 |
|  | PE(18:0/20:5) | PE | 1.92253 | 1.239603 |
|  | PE(20:1/20:4) | PE | 2.314052 | 1.270247 |
|  | PE(20:4/22:2) | PE | 1.145105 | 1.274316 |
|  | PG(18:0/18:1) | PG | 1.79834 | 0.62741 |
|  | PG(18:1/16:1) | PG | 1.279565 | 0.771445 |
|  | PI(20:3/18:0) | PI | 1.961933 | 0.648464 |
|  | PI(20:4/16:0) | PI | 1.357667 | 0.741589 |
|  | PI(18:1/20:4) | PI | 1.834026 | 0.654062 |
|  | PI(18:0/20:5) | PI | 1.25413 | 2.00017 |
|  | PS(18:0/20:4) | PS | 1.515379 | 1.289057 |
|  | Tiglyl-carnitine | CAR | 2.173652 | 1.96415 |
|  | CE(18:1) | CE | 1.548867 | 1.20717 |
|  | CE(18:2) | CE | 2.110327 | 1.402767 |
|  | CE(20:2) | CE | 1.107932 | 1.33165 |
|  | CE(20:4) | CE | 1.400074 | 1.269275 |
|  | Cer(d18:1/22:0) | Cer | 1.812207 | 1.333802 |
|  | Cer(d18:1/22:1) | Cer | 1.725784 | 1.210964 |
|  | Cer(d18:1/26:1) | Cer | 1.243155 | 1.269385 |
|  | Cer(m18:1/26:0) | Cerm | 1.739636 | 0.793027 |
|  | CerP(d18:1/16:1) | CerP | 1.763985 | 1.230127 |
|  | Cer(t18:0/24:0) | Cert | 1.448486 | 1.210839 |
|  | DG(16:0/18:0/0:0) | DG | 1.663227 | 0.81518 |
|  | DG(18:0/18:0/0:0) | DG | 1.822652 | 0.806928 |
|  | DG(16:0/20:1/0:0) | DG | 1.764986 | 1.280395 |
|  | DG(16:1/20:0/0:0) | DG | 1.235098 | 0.8235 |
|  | DG(14:0/18:2/0:0) | DG | 2.084405 | 1.506028 |
|  | DG(18:0/18:2/0:0) | DG | 1.771333 | 1.2166 |
|  | DG(20:0/18:2/0:0) | DG | 1.768626 | 1.549995 |
|  | DG(18:4/18:1/0:0) | DG | 1.050386 | 1.333311 |
|  | DG(18:2/20:3/0:0) | DG | 1.17503 | 1.40036 |
|  | LPC(12:0/0:0) | LPC | 1.567432 | 1.287993 |
|  | LPC(14:0/0:0) | LPC | 1.54243 | 1.242924 |
|  | LPC(18:0/0:0) | LPC | 1.460061 | 1.200328 |
|  | LPC(16:1/0:0) | LPC | 1.572859 | 1.240455 |
|  | LPC(18:3/0:0) | LPC | 1.049002 | 1.200526 |
|  | LPC(22:4/0:0) | LPC | 1.588673 | 1.272327 |
|  | LPC(O-16:0/0:0) | LPC-O | 1.969902 | 1.39311 |
|  | LPE(0:0/16:0) | LPE | 1.993051 | 1.222904 |
|  | LPE(0:0/24:0) | LPE | 1.181466 | 1.340425 |
|  | LPE(0:0/22:1) | LPE | 2.374275 | 1.299334 |
|  | LPE(0:0/22:4) | LPE | 2.180584 | 1.248394 |
|  | LPS(18:0/0:0) | LPS | 2.006247 | 2.284028 |
|  | PC(18:0/22:0) | PC | 2.115962 | 1.313628 |
|  | PC(20:5/12:0) | PC | 1.481927 | 0.8205 |
|  | PC(22:4/18:1) | PC | 1.608635 | 1.662478 |
|  | PC(18:1/20:5) | PC | 1.675822 | 1.354012 |
|  | PC(O-20:2/22:1) | PC-O | 1.730751 | 1.49774 |
|  | PE(22:2/12:0) | PE | 2.124806 | 1.452688 |
|  | PE(P-20:0/20:0) | PE-P | 1.10907 | 1.262595 |
|  | PE(P-20:1/20:0) | PE-P | 1.632291 | 1.220144 |
|  | PG(18:0/16:0) | PG | 2.001901 | 0.673167 |
|  | PS(20:4/20:0) | PS | 1.194147 | 1.241195 |
|  | SM(d18:0/16:0) | SM | 1.713318 | 1.331255 |
|  | SM(d18:0/18:1) | SM | 1.700715 | 0.740876 |
|  | SM(d18:2/24:1) | SM | 2.260924 | 1.271664 |
|  | TG(14:0/16:0/22:0) | TG | 1.137775 | 1.367232 |
|  | TG(14:0/20:0/22:0) | TG | 1.214616 | 1.323498 |
|  | TG(16:0/18:0/22:0) | TG | 1.896857 | 1.423549 |
|  | TG(16:0/20:0/22:0) | TG | 1.261701 | 1.476471 |
|  | TG(18:1/20:0/20:0) | TG | 2.137073 | 1.496741 |
|  | TG(14:0/22:0/22:2) | TG | 1.159369 | 0.802363 |
|  | TG(14:0/18:1/20:3) | TG | 1.25883 | 1.417793 |
|  | TG(18:1/18:1/18:2) | TG | 1.07833 | 0.738456 |
|  | TG(14:0/20:1/20:3) | TG | 1.561653 | 0.657376 |
|  | TG(18:2/18:2/20:0) | TG | 1.047389 | 1.266522 |
|  | TG(16:1/16:1/20:3) | TG | 1.098 | 1.34066 |
|  | TG(14:0/18:2/22:6) | TG | 1.402302 | 1.29592 |
|  | TG(14:0/20:5/22:4) | TG | 1.112487 | 0.71373 |
|  | TG(14:0/20:3/22:6) | TG | 1.083919 | 0.75791 |

Defined as having a VIP ＞ 1 and fold-change ＞ 1.2
