# Supplementary material for: Lipidomics profiling of goose granulosa cell model of stearoyl-CoA desaturase function identifies a pattern of lipid droplets associated with follicle development
Source: Cell Biosci. 2021 May 22;11:95. doi: 10.1186/s13578-021-00604-6 (PMC8141238; doi:10.1186/s13578-021-00604-6)
Supplement: Supplementary file 7 — Additional file 7: Table S2. A functional analysis of pathways related to the differentially abundant lipids. [file 13578_2021_604_MOESM7_ESM.docx]

| **Supplementary Table 2. A functional analysis of pathways related to the differentially abundant lipids** | | |
| --- | --- | --- |
| **pathway** | **P-value** | **compound** |
| **Comparison I: LN vs. LS comparison** | | |
| Regulation of lipolysis in adipocytes | 4.89E-07 | TG(18:0/18:2/18:3);TG(12:0/18:0/18:3);TG(14:0/18:0/18:2);TG(14:0/18:4/20:1);TG(14:0/14:0/20:1);TG(14:0/16:0/16:1);  TG(14:0/18:1/20:4);TG(14:1/16:1/18:1);TG(14:0/18:0/20:1);TG(14:0/18:2/22:0);TG(14:0/16:0/18:0);TG(14:0/18:0/18:3);  TG(14:0/14:1/18:1);TG(16:0/20:4/22:1);TG(16:0/20:4/22:3);TG(14:0/20:0/20:2);TG(14:0/22:1/22:2);TG(14:0/18:3/22:3);  TG(14:0/18:0/22:4);TG(14:0/16:0/22:4);TG(14:0/20:0/20:1);TG(12:0/18:1/18:3);FFA(17:1);TG(14:0/20:2/20:2);FFA(13:0);  TG(14:0/18:2/20:2);TG(16:0/16:1/22:4);TG(12:0/16:0/18:3);TG(14:0/16:0/22:5);TG(16:0/16:0/16:1);TG(14:0/16:1/18:3);  TG(14:0/16:0/18:1);TG(14:0/18:1/20:0);TG(14:0/20:4/22:2);TG(14:0/20:1/20:4);TG(14:1/14:1/18:1);TG(14:0/14:0/18:2);  TG(16:0/16:1/16:1);TG(18:0/18:1/18:2);TG(14:0/20:3/20:4);TG(14:0/20:3/22:1);FFA(16:1);TG(14:0/18:1/18:3);  TG(14:0/18:3/22:1);TG(16:0/16:1/22:3);TG(16:0/16:1/18:3);TG(14:0/18:0/20:4);FFA(24:1);TG(14:0/18:1/18:2);  TG(18:0/18:3/20:1);TG(14:1/16:0/16:1);TG(14:0/18:2/22:6);TG(14:0/20:2/20:5);TG(18:1/18:3/20:0);FFA(22:2);  TG(16:0/16:0/22:3);TG(14:0/16:1/18:1);TG(14:0/16:0/18:2);TG(14:0/16:0/16:0);TG(14:0/18:0/18:1);  TG(14:0/18:3/20:4);FFA(20:4);TG(12:0/14:0/18:1);TG(14:0/18:1/18:4);TG(14:0/16:1/20:4);TG(14:0/22:3/22:6);TG(14:0/22:0/22:2);TG(14:0/14:1/18:2);TG(14:0/20:3/22:6);FFA(20:2);TG(14:1/14:1/22:2);TG(16:1/20:4/20:4);FFA(22:0);FFA(18:2);TG(16:0/16:1/22:6);FFA(24:6);TG(12:0/22:1/22:3);TG(14:0/20:4/20:4);TG(16:0/16:0/18:0);TG(14:0/18:2/20:5);TG(14:0/20:1/20:1);TG(14:0/16:0/22:0);TG(12:0/16:0/16:1);TG(14:0/18:0/18:0);TG(16:1/18:1/20:0);TG(16:0/16:1/18:4);TG(14:0/18:0/20:3);TG(14:0/18:1/20:2);TG(14:0/14:0/18:1);TG(14:0/18:2/18:3);TG(16:0/16:1/20:2);TG(14:0/18:0/20:0);TG(14:0/18:1/20:3) |
| Cholesterol metabolism | 5.23E-07 | TG(16:0/16:0/22:3);TG(14:0/16:1/18:1);TG(14:0/16:0/18:2);TG(14:0/16:0/16:0);TG(14:0/18:0/18:1);TG(14:0/18:3/20:4);TG(12:0/14:0/18:1);TG(14:0/18:1/18:4);TG(14:0/16:1/20:4);TG(14:0/22:0/22:2);TG(14:0/22:3/22:6);TG(14:0/18:0/20:4);FFA(24:1);TG(14:0/18:1/18:2);TG(18:0/18:3/20:1);TG(14:0/18:2/22:6);TG(14:1/16:0/16:1);TG(14:0/20:2/20:5);TG(18:1/18:3/20:0);FFA(22:2);TG(16:0/16:0/18:0);TG(14:0/18:2/20:5);TG(14:0/20:1/20:1);TG(14:0/16:0/22:0);TG(12:0/16:0/16:1);TG(14:0/18:0/18:0);TG(16:1/18:1/20:0);TG(16:0/16:1/18:4);TG(14:0/18:0/20:3);TG(14:0/18:1/20:2);TG(14:0/14:0/18:1);TG(14:0/18:2/18:3);TG(16:0/16:1/20:2);TG(14:0/18:0/20:0);TG(14:0/18:1/20:3);TG(14:0/14:1/18:2);TG(14:0/20:3/22:6);FFA(20:2);TG(14:1/14:1/22:2);TG(16:1/20:4/20:4);FFA(22:0);FFA(18:2);FFA(24:6);TG(16:0/16:1/22:6);TG(12:0/22:1/22:3);TG(14:0/20:4/20:4);TG(14:0/22:1/22:2);TG(14:0/18:3/22:3);TG(14:0/18:0/22:4);TG(14:0/16:0/22:4);TG(14:0/20:0/20:1);TG(12:0/18:1/18:3);FFA(17:1);TG(14:0/20:2/20:2);FFA(13:0);TG(14:0/18:2/20:2);TG(12:0/18:0/18:3);TG(18:0/18:2/18:3);TG(14:0/18:0/18:2);TG(14:0/18:4/20:1);TG(14:0/14:0/20:1);TG(14:0/18:1/20:4);TG(14:0/16:0/16:1);TG(14:1/16:1/18:1);TG(14:0/18:0/20:1);TG(14:0/18:2/22:0);TG(14:0/16:0/18:0);TG(14:0/18:0/18:3);TG(14:0/14:1/18:1);TG(16:0/20:4/22:1);TG(16:0/20:4/22:3);TG(14:0/20:0/20:2);TG(14:0/20:3/22:1);TG(14:0/18:1/18:3);FFA(16:1);TG(14:0/18:3/22:1);TG(16:0/16:1/22:3);TG(16:0/16:1/18:3);TG(12:0/16:0/18:3);TG(16:0/16:1/22:4);TG(14:0/16:0/22:5);TG(16:0/16:0/16:1);TG(14:0/16:1/18:3);TG(14:0/16:0/18:1);TG(14:0/18:1/20:0);TG(14:0/20:4/22:2);TG(14:1/14:1/18:1);TG(14:0/20:1/20:4);TG(14:0/14:0/18:2);TG(18:0/18:1/18:2);TG(16:0/16:1/16:1);TG(14:0/20:3/20:4) |
| Thermogenesis | 9.43E-07 | TG(18:1/18:3/20:0);TG(14:0/20:2/20:5);TG(14:0/18:2/22:6);TG(14:1/16:0/16:1);TG(18:0/18:3/20:1);FFA(22:2);TG(14:0/18:0/20:4);TG(14:0/18:1/18:2);FFA(24:1);TG(14:0/18:1/18:4);TG(12:0/14:0/18:1);TG(14:0/18:0/18:1);TG(14:0/18:3/20:4);TG(14:0/22:3/22:6);TG(14:0/22:0/22:2);TG(14:0/16:1/20:4);TG(14:0/16:1/18:1);TG(16:0/16:0/22:3);TG(14:0/16:0/16:0);TG(14:0/16:0/18:2);TG(14:0/20:4/20:4);TG(12:0/22:1/22:3);FFA(24:6);TG(16:0/16:1/22:6);TG(14:1/14:1/22:2);FFA(20:2);TG(14:0/20:3/22:6);TG(14:0/14:1/18:2);FFA(18:2);FFA(22:0);TG(16:1/20:4/20:4);TG(14:0/18:1/20:2);TG(14:0/14:0/18:1);TG(14:0/18:0/20:3);TG(16:0/16:1/18:4);TG(14:0/18:1/20:3);TG(14:0/18:0/20:0);TG(16:0/16:1/20:2);TG(14:0/18:2/18:3);TG(14:0/20:1/20:1);TG(16:0/16:0/18:0);TG(14:0/18:2/20:5);TG(16:1/18:1/20:0);TG(14:0/18:0/18:0);TG(14:0/16:0/22:0);TG(12:0/16:0/16:1);TG(14:0/14:1/18:1);TG(14:0/18:0/18:3);TG(14:0/18:2/22:0);TG(14:0/16:0/18:0);TG(14:0/20:0/20:2);TG(16:0/20:4/22:1);TG(16:0/20:4/22:3);TG(14:0/18:4/20:1);TG(14:0/18:0/18:2);TG(18:0/18:2/18:3);TG(12:0/18:0/18:3);TG(14:0/18:0/20:1);TG(14:1/16:1/18:1);TG(14:0/18:1/20:4);TG(14:0/16:0/16:1);TG(14:0/14:0/20:1);FFA(17:1);TG(12:0/18:1/18:3);TG(14:0/20:0/20:1);TG(14:0/16:0/22:4);TG(14:0/18:2/20:2);FFA(13:0);TG(14:0/20:2/20:2);TG(14:0/18:3/22:3);TG(14:0/22:1/22:2);TG(14:0/18:0/22:4);TG(14:0/14:0/18:2);TG(14:0/20:3/20:4);TG(18:0/18:1/18:2);TG(16:0/16:1/16:1);TG(16:0/16:0/16:1);TG(14:0/16:0/22:5);TG(16:0/16:1/22:4);TG(12:0/16:0/18:3);TG(14:0/20:4/22:2);TG(14:0/20:1/20:4);TG(14:1/14:1/18:1);TG(14:0/18:1/20:0);TG(14:0/16:0/18:1);TG(14:0/16:1/18:3);TG(14:0/18:3/22:1);TG(14:0/18:1/18:3);FFA(16:1);TG(16:0/16:1/18:3);TG(16:0/16:1/22:3);TG(14:0/20:3/22:1) |
| Vitamin digestion and absorption | 1.26E-06 | TG(14:0/20:3/22:1);FFA(16:1);TG(14:0/18:1/18:3);TG(14:0/18:3/22:1);TG(16:0/16:1/22:3);TG(16:0/16:1/18:3);TG(16:0/16:1/22:4);TG(12:0/16:0/18:3);TG(14:0/16:0/22:5);TG(16:0/16:0/16:1);TG(14:0/16:1/18:3);TG(14:0/16:0/18:1);TG(14:0/18:1/20:0);TG(14:0/20:1/20:4);TG(14:1/14:1/18:1);TG(14:0/20:4/22:2);TG(14:0/14:0/18:2);TG(16:0/16:1/16:1);TG(18:0/18:1/18:2);TG(14:0/20:3/20:4);TG(14:0/22:1/22:2);TG(14:0/18:3/22:3);TG(14:0/18:0/22:4);TG(14:0/16:0/22:4);TG(14:0/20:0/20:1);TG(12:0/18:1/18:3);FFA(17:1);TG(14:0/20:2/20:2);FFA(13:0);TG(14:0/18:2/20:2);TG(18:0/18:2/18:3);TG(12:0/18:0/18:3);TG(14:0/18:0/18:2);TG(14:0/18:4/20:1);TG(14:0/14:0/20:1);TG(14:0/18:1/20:4);TG(14:0/16:0/16:1);TG(14:1/16:1/18:1);TG(14:0/18:0/20:1);TG(14:0/16:0/18:0);TG(14:0/18:2/22:0);TG(14:0/18:0/18:3);TG(14:0/14:1/18:1);TG(16:0/20:4/22:3);TG(16:0/20:4/22:1);TG(14:0/20:0/20:2);TG(14:0/18:2/20:5);TG(16:0/16:0/18:0);TG(14:0/20:1/20:1);TG(12:0/16:0/16:1);TG(14:0/16:0/22:0);TG(14:0/18:0/18:0);TG(16:1/18:1/20:0);TG(16:0/16:1/18:4);TG(14:0/18:0/20:3);TG(14:0/14:0/18:1);TG(14:0/18:1/20:2);TG(14:0/18:2/18:3);TG(16:0/16:1/20:2);TG(14:0/18:0/20:0);TG(14:0/18:1/20:3);TG(14:0/14:1/18:2);TG(14:0/20:3/22:6);FFA(20:2);TG(14:1/14:1/22:2);TG(16:1/20:4/20:4);FFA(22:0);FFA(18:2);FFA(24:6);TG(16:0/16:1/22:6);TG(12:0/22:1/22:3);TG(14:0/20:4/20:4);TG(16:0/16:0/22:3);TG(14:0/16:1/18:1);TG(14:0/16:0/18:2);TG(14:0/16:0/16:0);TG(14:0/18:3/20:4);TG(14:0/18:0/18:1);TG(12:0/14:0/18:1);TG(14:0/18:1/18:4);TG(14:0/16:1/20:4);TG(14:0/22:3/22:6);TG(14:0/22:0/22:2);TG(14:0/18:0/20:4);FFA(24:1);TG(14:0/18:1/18:2);TG(18:0/18:3/20:1);TG(14:0/18:2/22:6);TG(14:0/20:2/20:5);TG(14:1/16:0/16:1);TG(18:1/18:3/20:0);FFA(22:2) |
| Fat digestion and absorption | 2.55E-06 | TG(16:0/16:1/22:4);TG(12:0/16:0/18:3);TG(16:0/16:0/16:1);TG(14:0/16:0/22:5);TG(14:0/16:0/18:1);TG(14:0/16:1/18:3);TG(14:0/20:1/20:4);TG(14:1/14:1/18:1);TG(14:0/20:4/22:2);TG(14:0/18:1/20:0);TG(14:0/14:0/18:2);TG(18:0/18:1/18:2);TG(16:0/16:1/16:1);PA(18:0/18:1);TG(14:0/20:3/20:4);TG(14:0/20:3/22:1);TG(14:0/18:1/18:3);FFA(16:1);TG(14:0/18:3/22:1);TG(16:0/16:1/22:3);TG(16:0/16:1/18:3);TG(14:0/18:0/18:2);TG(12:0/18:0/18:3);TG(18:0/18:2/18:3);TG(14:0/18:4/20:1);TG(14:0/16:0/16:1);TG(14:0/18:1/20:4);TG(14:0/14:0/20:1);TG(14:0/18:0/20:1);TG(14:1/16:1/18:1);TG(14:0/16:0/18:0);TG(14:0/18:2/22:0);TG(14:0/14:1/18:1);TG(14:0/18:0/18:3);TG(14:0/20:0/20:2);TG(16:0/20:4/22:3);TG(16:0/20:4/22:1);TG(14:0/18:3/22:3);TG(14:0/22:1/22:2);TG(14:0/18:0/22:4);TG(14:0/16:0/22:4);TG(12:0/18:1/18:3);FFA(17:1);TG(14:0/20:0/20:1);TG(14:0/20:2/20:2);TG(14:0/18:2/20:2);FFA(13:0);TG(14:0/20:3/22:6);TG(14:0/14:1/18:2);TG(14:1/14:1/22:2);FFA(20:2);TG(16:1/20:4/20:4);FFA(18:2);FFA(22:0);FFA(24:6);TG(16:0/16:1/22:6);TG(14:0/20:4/20:4);TG(12:0/22:1/22:3);TG(14:0/18:2/20:5);TG(16:0/16:0/18:0);PA(20:1/18:0)  ;TG(14:0/20:1/20:1);TG(12:0/16:0/16:1);TG(14:0/16:0/22:0);TG(16:1/18:1/20:0);TG(14:0/18:0/18:0);TG(14:0/18:0/20:3);TG(16:0/16:1/18:4);TG(14:0/14:0/18:1);TG(14:0/18:1/20:2);TG(16:0/16:1/20:2);TG(14:0/18:2/18:3);TG(14:0/18:1/20:3);TG(14:0/18:0/20:0);TG(14:0/18:0/20:4);FFA(24:1);TG(14:0/18:1/18:2);TG(18:0/18:3/20:1);TG(18:1/18:3/20:0);TG(14:0/20:2/20:5);TG(14:0/18:2/22:6);TG(14:1/16:0/16:1);FFA(22:2);TG(16:0/16:0/22:3);TG(14:0/16:1/18:1);TG(14:0/16:0/18:2);TG(14:0/16:0/16:0);TG(12:0/14:0/18:1);TG(14:0/18:3/20:4);TG(14:0/18:0/18:1);TG(14:0/18:1/18:4);TG(14:0/16:1/20:4);TG(14:0/22:3/22:6);TG(14:0/22:0/22:2) |
| Insulin resistance | 5.73E-06 | TG(16:0/20:4/22:3);TG(16:0/20:4/22:1);TG(14:0/20:0/20:2);TG(14:0/14:1/18:1);TG(14:0/18:0/18:3);TG(14:0/16:0/18:0);TG(14:0/18:2/22:0);TG(14:1/16:1/18:1);TG(14:0/18:0/20:1);TG(14:0/14:0/20:1);TG(14:0/18:1/20:4);TG(14:0/16:0/16:1);TG(14:0/18:4/20:1);TG(18:0/18:2/18:3);TG(12:0/18:0/18:3);TG(14:0/18:0/18:2);FFA(13:0);TG(14:0/18:2/20:2);TG(14:0/20:2/20:2);TG(14:0/20:0/20:1);FFA(17:1);TG(12:0/18:1/18:3);TG(14:0/16:0/22:4);CER(D18:0/22:0);TG(14:0/18:0/22:4);TG(14:0/22:1/22:2);TG(14:0/18:3/22:3);TG(14:0/20:3/20:4);TG(18:0/18:1/18:2);TG(16:0/16:1/16:1);TG(14:0/14:0/18:2);TG(14:0/18:1/20:0);TG(14:0/20:1/20:4);TG(14:1/14:1/18:1);TG(14:0/20:4/22:2);TG(14:0/16:1/18:3);TG(14:0/16:0/18:1);TG(14:0/16:0/22:5);TG(16:0/16:0/16:1);TG(16:0/16:1/22:4);TG(12:0/16:0/18:3);TG(16:0/16:1/18:3);TG(16:0/16:1/22:3);TG(14:0/18:3/22:1);TG(14:0/18:1/18:3);FFA(16:1);TG(14:0/20:3/22:1);FFA(22:2);TG(14:0/18:2/22:6);TG(14:0/20:2/20:5);TG(14:1/16:0/16:1);TG(18:1/18:3/20:0);TG(18:0/18:3/20:1);TG(14:0/18:1/18:2);FFA(24:1);TG(14:0/18:0/20:4);TG(14:0/22:3/22:6);TG(14:0/22:0/22:2);TG(14:0/16:1/20:4);TG(14:0/18:1/18:4);TG(14:0/18:3/20:4);CER(D18:0/20:0);TG(14:0/18:0/18:1);TG(12:0/14:0/18:1);TG(14:0/16:0/16:0);TG(14:0/16:0/18:2);TG(14:0/16:1/18:1);TG(16:0/16:0/22:3);TG(12:0/22:1/22:3);TG(14:0/20:4/20:4);TG(16:0/16:1/22:6);FFA(24:6);FFA(22:0);FFA(18:2);TG(16:1/20:4/20:4);FFA(20:2);TG(14:1/14:1/22:2);TG(14:0/14:1/18:2);TG(14:0/20:3/22:6);TG(14:0/18:0/20:0);TG(14:0/18:1/20:3);TG(14:0/18:2/18:3);TG(16:0/16:1/20:2);TG(14:0/14:0/18:1);TG(14:0/18:1/20:2);TG(16:0/16:1/18:4);TG(14:0/18:0/20:3);TG(14:0/18:0/18:0);TG(16:1/18:1/20:0);TG(12:0/16:0/16:1);TG(14:0/16:0/22:0);TG(14:0/20:1/20:1);TG(14:0/18:2/20:5);TG(16:0/16:0/18:0) |
| Glycerolipid metabolism | 0.002669 | DG(16:1/18:0/0:0);TG(16:0/16:0/22:3);TG(14:0/16:1/18:1);DG(18:2/22:4/0:0);TG(14:0/16:0/18:2);TG(14:0/16:0/16:0);TG(14:0/18:0/18:1);TG(14:0/18:3/20:4);TG(12:0/14:0/18:1);TG(14:0/18:1/18:4);TG(14:0/16:1/20:4);TG(14:0/22:0/22:2);TG(14:0/22:3/22:6);TG(14:0/18:0/20:4);FFA(24:1);TG(14:0/18:1/18:2);TG(18:0/18:3/20:1);TG(14:0/18:2/22:6);TG(14:0/20:2/20:5);TG(14:1/16:0/16:1);TG(18:1/18:3/20:0);FFA(22:2);PA(20:1/18:0);TG(16:0/16:0/18:0);DG(14:0/18:1/0:0);DG(18:1/22:5/0:0);TG(14:0/18:2/20:5);DG(14:0/18:2/0:0);TG(14:0/20:1/20:1);TG(14:0/16:0/22:0);TG(12:0/16:0/16:1);TG(14:0/18:0/18:0);TG(16:1/18:1/20:0);TG(16:0/16:1/18:4);TG(14:0/18:0/20:3);TG(14:0/18:1/20:2);TG(14:0/14:0/18:1);TG(14:0/18:2/18:3);DG(16:1/16:1/0:0);TG(16:0/16:1/20:2);TG(14:0/18:0/20:0);TG(14:0/18:1/20:3);TG(14:0/14:1/18:2);TG(14:0/20:3/22:6);FFA(20:2);TG(14:1/14:1/22:2);TG(16:1/20:4/20:4);FFA(22:0);FFA(18:2);FFA(24:6);TG(16:0/16:1/22:6);TG(12:0/22:1/22:3);TG(14:0/20:4/20:4);DG(16:0/20:0/0:0);TG(14:0/22:1/22:2);TG(14:0/18:3/22:3);TG(14:0/18:0/22:4);TG(14:0/16:0/22:4);TG(14:0/20:0/20:1);TG(12:0/18:1/18:3);FFA(17:1);TG(14:0/20:2/20:2);FFA(13:0);TG(14:0/18:2/20:2);TG(12:0/18:0/18:3);TG(18:0/18:2/18:3);TG(14:0/18:0/18:2);TG(14:0/18:4/20:1);TG(14:0/14:0/20:1);TG(14:0/18:1/20:4);TG(14:0/16:0/16:1);TG(14:1/16:1/18:1);TG(14:0/18:0/20:1);TG(14:0/18:2/22:0);TG(14:0/16:0/18:0);TG(14:0/14:1/18:1);TG(14:0/18:0/18:3);TG(16:0/20:4/22:1);TG(16:0/20:4/22:3);TG(14:0/20:0/20:2);TG(14:0/20:3/22:1);FFA(16:1);TG(14:0/18:1/18:3);TG(14:0/18:3/22:1);TG(16:0/16:1/22:3);TG(16:0/16:1/18:3);TG(16:0/16:1/22:4);TG(12:0/16:0/18:3);TG(14:0/16:0/22:5);TG(16:0/16:0/16:1);TG(14:0/16:1/18:3);TG(14:0/16:0/18:1);TG(14:0/18:1/20:0);TG(14:0/20:4/22:2);TG(14:1/14:1/18:1);TG(14:0/20:1/20:4);TG(14:0/14:0/18:2);PA(18:0/18:1);TG(16:0/16:1/16:1);TG(18:0/18:1/18:2);TG(14:0/20:3/20:4) |
| Amoebiasis | 0.010131 | PS(18:0/16:1);PS(20:4/20:0);FFA(20:4);PS(18:0/22:6);PS(20:5/18:0) |
| Systemic lupus erythematosus | 0.029214 | PS(20:5/18:0);PS(18:0/22:6);PS(18:0/16:1);PS(20:4/20:0) |
| Glycine, serine and threonine metabolism | 0.029214 | PS(18:0/22:6);PS(20:5/18:0);PS(20:4/20:0);PS(18:0/16:1) |
| Inflammatory mediator regulation of TRP channels | 0.297445 | FFA(20:4) |
| Aldosterone synthesis and secretion | 0.297445 | FFA(20:4) |
| Serotonergic synapse | 0.297445 | FFA(20:4) |
| Phototransduction - fly | 0.297445 | FFA(20:4) |
| Fc epsilon RI signaling pathway | 0.297445 | FFA(20:4) |
| Vascular smooth muscle contraction | 0.297445 | FFA(20:4) |
| Ovarian steroidogenesis | 0.297445 | FFA(20:4) |
| Biosynthesis of unsaturated fatty acids | 0.297445 | FFA(20:4) |
| Ferroptosis | 0.297445 | FFA(20:4) |
| Platelet activation | 0.297445 | FFA(20:4) |
| Oxytocin signaling pathway | 0.297445 | FFA(20:4) |
| Leishmaniasis | 0.438999 | CER(D18:0/20:0);FFA(20:4);PS(20:4/20:0);CER(D18:0/22:0);PS(18:0/16:1);PS(20:5/18:0);PS(18:0/22:6) |
| Metabolic pathways | 0.580626 | PC(14:1/18:2);SM(D18:0/18:0);TG(14:0/18:0/20:4);TG(14:0/18:1/18:2);TG(14:0/18:2/22:6);TG(14:0/20:2/20:5);TG(14:1/16:0/16:1);TG(18:1/18:3/20:0);SM(D18:0/24:0);TG(18:0/18:3/20:1);PE(18:1/18:1);TG(14:0/16:1/18:1);DG(18:2/22:4/0:0);DG(16:1/18:0/0:0);TG(16:0/16:0/22:3);TG(14:0/16:0/16:0);PC(18:1/20:4);PE(24:0/18:1);PE(22:6/18:0);PE(22:1/20:1);TG(14:0/16:0/18:2);TG(14:0/18:1/18:4);TG(14:0/18:3/20:4);FFA(20:4);CER(D18:0/20:0);TG(14:0/18:0/18:1);TG(12:0/14:0/18:1);TG(14:0/22:0/22:2);TG(14:0/22:3/22:6);TG(14:0/16:1/20:4);TG(14:1/14:1/22:2);SM(D18:0/14:0);TG(14:0/14:1/18:2);PC(16:0/20:3);TG(14:0/20:3/22:6);TG(16:1/20:4/20:4);TG(12:0/22:1/22:3);TG(14:0/20:4/20:4);SM(D18:0/22:0);TG(16:0/16:1/22:6);PC(20:4/20:4);PC(22:2/22:6);DG(16:0/20:0/0:0);DG(14:0/18:2/0:0);PC(18:1/18:4);PE(22:0/18:0);TG(14:0/20:1/20:1);TG(14:0/18:2/20:5);DG(18:1/22:5/0:0);PA(20:1/18:0);TG(16:0/16:0/18:0);DG(14:0/18:1/0:0);PE(22:2/12:0);PE(18:2/14:1);TG(14:0/18:0/18:0);PE(20:1/16:1);TG(16:1/18:1/20:0);TG(12:0/16:0/16:1);TG(14:0/16:0/22:0);TG(14:0/14:0/18:1);TG(14:0/18:1/20:2);PC(14:0/18:1);PC(18:0/22:6);PC(14:0/18:2);PG(18:0/16:0);TG(16:0/16:1/18:4);PC(18:0/14:1);TG(14:0/18:0/20:3);TG(14:0/18:0/20:0);TG(14:0/18:1/20:3);DG(16:1/16:1/0:0);TG(14:0/18:2/18:3);PI(18:1/20:4);PC(18:0/22:0);PG(18:2/18:1);TG(16:0/16:1/20:2);TG(14:0/18:4/20:1);TG(18:0/18:2/18:3);TG(12:0/18:0/18:3);TG(14:0/18:0/18:2);TG(14:1/16:1/18:1);TG(14:0/18:0/20:1);TG(14:0/14:0/20:1);TG(14:0/18:1/20:4);TG(14:0/16:0/16:1);PE(18:0/18:2);PC(18:2/20:4);TG(14:0/14:1/18:1);TG(14:0/18:0/18:3);TG(14:0/16:0/18:0);TG(14:0/18:2/22:0);TG(16:0/20:4/22:3);TG(16:0/20:4/22:1);TG(14:0/20:0/20:2);PC(18:2/20:3);PE(20:1/20:5);TG(14:0/22:1/22:2);TG(14:0/18:3/22:3);PE(18:2/14:0);TG(14:0/18:0/22:4);SM(D18:0/18:1);PC(20:5/12:0);PE(22:0/18:1);TG(14:0/20:0/20:1);PC(18:3/14:1);TG(12:0/18:1/18:3);PS(20:5/18:0);TG(14:0/16:0/22:4);CER(D18:0/22:0);TG(14:0/18:2/20:2);TG(14:0/20:2/20:2);TG(14:0/16:0/22:5);TG(16:0/16:0/16:1);TG(16:0/16:1/22:4);TG(12:0/16:0/18:3);PS(18:0/16:1);TG(14:0/18:1/20:0);TG(14:1/14:1/18:1);TG(14:0/20:1/20:4);PE(18:4/20:0);TG(14:0/20:4/22:2);TG(14:0/16:1/18:3);TG(14:0/16:0/18:1);TG(14:0/14:0/18:2);PC(20:5/18:0);PE(22:2/14:0);TG(14:0/20:3/20:4);PA(18:0/18:1);TG(18:0/18:1/18:2);TG(16:0/16:1/16:1);SM(D18:1/20:1);PC(16:0/18:3);PC(18:0/18:2);PG(18:1/20:4);TG(14:0/20:3/22:1);TG(14:0/18:3/22:1);TG(14:0/18:1/18:3);PC(16:1/18:3);PS(18:0/22:6);TG(16:0/16:1/18:3);PS(20:4/20:0);TG(16:0/16:1/22:3) |
| Type II diabetes mellitus | 0.60568 | FFA(24:6);FFA(16:1);FFA(17:1);FFA(20:2);FFA(24:1);FFA(22:2);FFA(18:2);FFA(13:0);FFA(22:0) |
| Non-alcoholic fatty liver disease (NAFLD) | 0.60568 | FFA(13:0);FFA(22:0);FFA(18:2);FFA(22:2);FFA(24:1);FFA(20:2);FFA(17:1);FFA(16:1);FFA(24:6) |
| AMPK signaling pathway | 0.60568 | FFA(22:0);FFA(13:0);FFA(18:2);FFA(22:2);FFA(24:1);FFA(20:2);FFA(17:1);FFA(16:1);FFA(24:6) |
| Insulin secretion | 0.60568 | FFA(16:1);FFA(24:6);FFA(20:2);FFA(17:1);FFA(24:1);FFA(13:0);FFA(22:0);FFA(18:2);FFA(22:2) |
| Fatty acid degradation | 0.60568 | FFA(22:2);FFA(18:2);FFA(22:0);FFA(13:0);FFA(24:1);FFA(17:1);FFA(20:2);FFA(24:6);FFA(16:1) |
| Fatty acid elongation | 0.60568 | FFA(24:1);FFA(18:2);FFA(22:2);FFA(22:0);FFA(13:0);FFA(24:6);FFA(16:1);FFA(17:1);FFA(20:2) |
| GnRH signaling pathway | 0.612052 | PA(20:1/18:0);PA(18:0/18:1);FFA(20:4) |
| Fc gamma R-mediated phagocytosis | 0.612052 | PA(18:0/18:1);PA(20:1/18:0);FFA(20:4) |
| Linoleic acid metabolism | 0.775662 | PC(20:4/20:4);PC(18:2/20:3);PC(18:0/22:0);PC(22:2/22:6);PC(18:0/22:6);PC(14:0/18:1);PC(18:2/20:4);PC(14:0/18:2);PC(18:3/14:1);PC(18:0/14:1);PC(20:5/18:0);FFA(20:4);PC(16:1/18:3);PC(18:1/20:4);PC(20:5/12:0);PC(18:1/18:4);PC(14:1/18:2);PC(16:0/18:3);PC(18:0/18:2);PC(16:0/20:3) |
| Arachidonic acid metabolism | 0.798313 | PC(18:0/22:0);PC(22:2/22:6);PC(18:2/20:3);PC(20:4/20:4);PC(16:1/18:3);FFA(20:4);PC(18:0/14:1);PC(20:5/18:0);PC(18:3/14:1);PC(14:0/18:2);PC(18:2/20:4);PC(14:0/18:1);PC(18:0/22:6);PC(20:5/12:0);PC(18:1/20:4);PC(16:0/20:3);PC(18:0/18:2);PC(16:0/18:3);PC(14:1/18:2);PC(18:1/18:4) |
| Pancreatic cancer | 0.801706 | PA(18:0/18:1);PA(20:1/18:0) |
| cAMP signaling pathway | 0.801706 | PA(20:1/18:0);PA(18:0/18:1) |
| Choline metabolism in cancer | 0.82809 | FFA(13:0);LPC(18:3/0:0);PC(18:0/22:0);LPC(18:2/0:0);LPC(22:4/0:0);PC(18:0/22:6);PC(14:0/18:1);FFA(17:1);PC(18:3/14:1);PC(14:0/18:2);FFA(16:1);PC(18:0/14:1);PC(16:1/18:3);PC(18:1/20:4);PC(20:5/12:0);PC(18:1/18:4);PC(18:0/18:2);PC(16:0/18:3);PA(20:1/18:0);PC(20:4/20:4);PC(18:2/20:3);LPC(16:1/0:0);FFA(22:2);PA(18:0/18:1);PC(22:2/22:6);PC(18:2/20:4);PC(20:5/18:0);FFA(24:6);LPC(22:6/0:0);FFA(22:0);FFA(18:2);FFA(24:1);FFA(20:2);LPC(20:3/0:0);PC(14:1/18:2);PC(16:0/20:3) |
| Tuberculosis | 0.830167 | PI(18:1/20:4) |
| Salmonella infection | 0.830167 | PI(18:1/20:4) |
| alpha-Linolenic acid metabolism | 0.831215 | PC(18:1/20:4);PC(20:5/12:0);PC(18:1/18:4);PC(18:0/18:2);PC(16:0/18:3);PC(14:1/18:2);PC(16:0/20:3);PC(20:4/20:4);PC(18:2/20:3);PC(22:2/22:6);PC(18:0/22:0);PC(18:2/20:4);PC(18:0/22:6);PC(14:0/18:1);PC(18:3/14:1)  ;PC(14:0/18:2);PC(20:5/18:0);PC(18:0/14:1);PC(16:1/18:3) |
| Phospholipase D signaling pathway | 0.84912 | PA(20:1/18:0);PA(18:0/18:1) |
| Pathways in cancer | 0.84912 | PA(18:0/18:1);PA(20:1/18:0) |
| Adipocytokine signaling pathway | 0.85907 | FFA(22:0);FFA(18:2);FFA(24:1);FFA(20:2);FFA(13:0);FFA(22:2);FFA(17:1);FFA(16:1);CER(D18:0/20:0);FFA(24:6);CER(D18:0/22:0) |
| Necroptosis | 0.908429 | SM(D18:0/18:0);SM(D18:0/22:0);CER(D18:0/20:0);SM(D18:0/24:0);SM(D18:0/14:0);FFA(20:4);CER(D18:0/22:0);SM(D18:0/18:1);SM(D18:1/20:1) |
| Sphingolipid signaling pathway | 0.947446 | CER(D18:0/20:0);SM(D18:0/24:0);SM(D18:0/14:0);CER(D18:0/22:0);SM(D18:0/22:0);SM(D18:0/18:0);SM(D18:1/20:1);SM(D18:0/18:1) |
| Sphingolipid metabolism | 0.947446 | SM(D18:0/18:1);SM(D18:1/20:1);SM(D18:0/18:0);SM(D18:0/22:0);SM(D18:0/14:0);SM(D18:0/24:0);CER(D18:0/20:0);CER(D18:0/22:0) |
| Neurotrophin signaling pathway | 0.964968 | CER(D18:0/20:0);CER(D18:0/22:0) |
| AGE-RAGE signaling pathway in diabetic complications | 0.964968 | CER(D18:0/20:0);CER(D18:0/22:0) |
| Retrograde endocannabinoid signaling | 0.998092 | PE(18:2/14:0);PE(20:1/16:1);PE(22:6/18:0);PE(24:0/18:1);PC(20:5/12:0);PC(18:1/20:4);PE(18:2/14:1);PE(22:1/20:1);PE(22:0/18:1);PE(22:0/18:0);PE(20:1/20:5);PC(16:0/18:3);PC(18:0/18:2);PC(18:1/18:4);PE(22:2/12:0);PC(18:0/22:0);PC(18:3/14:1);PC(14:0/18:2);PC(14:0/18:1);PC(18:0/22:6);PC(16:1/18:3);FFA(20:4);PC(18:0/14:1);PE(18:4/20:0);PC(14:1/18:2);PC(16:0/20:3);PC(18:2/20:3);PC(20:4/20:4);PC(22:2/22:6);PC(18:2/20:4);PE(18:0/18:2);PE(22:2/14:0);PE(18:1/18:1);PC(20:5/18:0) |
| Long-term depression | 0.998647 | DG(14:0/18:2/0:0);DG(18:2/22:4/0:0);FFA(20:4);DG(18:1/22:5/0:0);DG(14:0/18:1/0:0);DG(16:1/18:0/0:0);DG(16:1/16:1/0:0);DG(16:0/20:0/0:0) |
| Kaposi sarcoma-associated herpesvirus infection | 0.999075 | PE(22:2/12:0);PE(20:1/20:5);PE(22:0/18:0);PE(22:0/18:1);PE(22:1/20:1);PE(18:2/14:1);PE(20:1/16:1);PE(18:2/14:0)  ;PE(24:0/18:1);PE(18:4/20:0);PE(22:6/18:0);PE(22:2/14:0);PE(18:1/18:1);PE(18:0/18:2) |
| Glycosylphosphatidylinositol (GPI)-anchor biosynthesis | 0.999226 | PE(22:2/14:0);PE(18:1/18:1);PE(18:0/18:2);PI(18:1/20:4);PE(22:2/12:0);PE(22:0/18:0);PE(20:1/20:5);PE(22:1/20:1);PE(22:0/18:1);PE(18:2/14:0);PE(20:1/16:1);PE(18:4/20:0);PE(22:6/18:0);PE(24:0/18:1);PE(18:2/14:1) |
| Autophagy - other | 0.999226 | PI(18:1/20:4);PE(18:1/18:1);PE(22:2/14:0);PE(18:0/18:2);PE(22:0/18:1);PE(22:1/20:1);PE(18:2/14:1);PE(24:0/18:1);PE(18:4/20:0);PE(22:6/18:0);PE(18:2/14:0);PE(20:1/16:1);PE(22:2/12:0);PE(20:1/20:5);PE(22:0/18:0) |
| Autophagy - animal | 0.999226 | PE(22:2/12:0);PE(22:0/18:0);PE(20:1/20:5);PE(22:1/20:1);PE(22:0/18:1);PE(20:1/16:1);PE(18:2/14:0);PE(18:4/20:0);PE(24:0/18:1);PE(22:6/18:0);PE(18:2/14:1);PE(22:2/14:0);PE(18:1/18:1);PE(18:0/18:2);PI(18:1/20:4) |
| Pathogenic Escherichia coli infection | 0.999267 | PE(22:2/12:0);PE(22:0/18:0);PE(20:1/20:5);PE(22:0/18:1);PE(22:1/20:1);PE(18:2/14:1);PE(22:6/18:0);PE(18:4/20:0);PE(24:0/18:1);PE(18:2/14:0);PE(20:1/16:1);PE(18:1/18:1);PE(22:2/14:0);PE(18:0/18:2) |
| Phosphatidylinositol signaling system | 0.999497 | DG(16:0/20:0/0:0);PA(18:0/18:1);PI(18:1/20:4);DG(16:1/16:1/0:0);DG(16:1/18:0/0:0);DG(14:0/18:1/0:0);PA(20:1/18:0);DG(18:1/22:5/0:0);DG(18:2/22:4/0:0);DG(14:0/18:2/0:0) |
| Inositol phosphate metabolism | 0.999527 | DG(16:1/18:0/0:0);DG(14:0/18:1/0:0);DG(18:1/22:5/0:0);DG(18:2/22:4/0:0);DG(14:0/18:2/0:0);DG(16:0/20:0/0:0);PI(18:1/20:4);DG(16:1/16:1/0:0) |
| Glycerophospholipid metabolism | 0.999986 | DG(16:1/18:0/0:0);DG(18:2/22:4/0:0);PE(20:1/20:5);PE(22:0/18:1);PE(22:1/20:1);PC(18:1/20:4);PC(20:5/12:0);PE(24:0/18:1);PE(22:6/18:0);PE(18:2/14:0);LPC(18:2/0:0);PS(20:5/18:0);PC(18:3/14:1);LPC(18:3/0:0);PC(14:1/18:2);LPE(0:0/22:4);PE(18:1/18:1);PC(18:2/20:4);PE(18:0/18:2);LPE(0:0/20:3)  ;LPC(16:1/0:0);PC(18:2/20:3);DG(14:0/18:1/0:0);PA(20:1/18:0);DG(18:1/22:5/0:0);PG(18:1/20:4);PE(22:2/12:0);PC(18:1/18:4);DG(14:0/18:2/0:0);PE(22:0/18:0);PC(16:0/18:3);PC(18:0/18:2);PE(18:2/14:1);LPE(0:0/22:6)  ;PE(20:1/16:1);PC(18:0/14:1);PG(18:0/16:0);PC(16:1/18:3);PC(18:0/22:6);PC(14:0/18:1);LPC(22:4/0:0);LPE(0:0/18:1)  ;LPE(0:0/18:0);PC(14:0/18:2);PI(18:1/20:4);PG(18:2/18:1);PC(18:0/22:0);DG(16:1/16:1/0:0)  ;PS(20:4/20:0);LPE(0:0/16:0);PS(18:0/22:6);PS(18:0/16:1);PC(16:0/20:3);LPC(20:3/0:0);LPC(22:6/0:0);PE(18:4/20:0)  ;PC(20:5/18:0);PE(22:2/14:0);PC(22:2/22:6);PA(18:0/18:1);DG(16:0/20:0/0:0);PC(20:4/20:4) |
| **Comparison I: LG vs. LS comparison** | | |
| Cholesterol metabolism | 0.060984655 | TG(14:0/20:4/22:0);TG(18:2/18:3/20:2);TG(16:1/16:1/20:5);TG(16:1/20:2/22:0);FFA(17:0);TG(16:0/18:0/22:0);TG(18:2/18:3/20:3);TG(14:0/18:0/18:0);TG(14:0/16:1/22:6);FFA(18:0);TG(16:0/16:1/20:4);TG(14:0/20:4/22:4);TG(14:0/20:5/22:4);TG(14:0/20:0/22:3);FFA(20:0);TG(16:0/16:0/18:1);FFA(6:0);TG(16:0/18:3/22:5);TG(14:0/20:1/22:5);TG(18:1/20:1/20:2);TG(14:0/18:4/20:1);FFA(4:0);TG(16:0/20:4/22:2);TG(14:0/22:0/22:2);TG(14:0/22:1/22:2);TG(16:0/16:1/20:5);TG(16:0/16:1/22:5);TG(14:0/20:1/20:3);TG(12:0/18:1/18:3);TG(14:0/22:4/22:4);TG(16:0/16:1/18:4);FFA(22:2);TG(18:0/18:3/20:3);TG(14:0/18:3/22:3);TG(16:0/20:0/22:0);TG(14:0/18:2/18:3) |
| Thermogenesis | 0.069793052 | FFA(20:0);TG(16:0/16:0/18:1);FFA(6:0);TG(16:0/18:3/22:5);TG(14:0/20:5/22:4);TG(14:0/20:0/22:3);TG(18:2/18:3/20:3);TG(14:0/16:1/22:6);TG(14:0/18:0/18:0);TG(14:0/20:4/22:4);TG(16:0/16:1/20:4);FFA(18:0);TG(14:0/20:4/22:0);TG(16:1/16:1/20:5);TG(18:2/18:3/20:2);TG(16:0/18:0/22:0);TG(16:1/20:2/22:0);FFA(17:0);TG(14:0/18:3/22:3);TG(16:0/20:0/22:0);TG(14:0/18:2/18:3);TG(16:0/16:1/18:4);TG(18:0/18:3/20:3);FFA(22:2);TG(16:0/16:1/20:5);TG(14:0/22:0/22:2);TG(14:0/22:1/22:2);TG(16:0/16:1/22:5);TG(14:0/22:4/22:4);TG(12:0/18:1/18:3);TG(14:0/20:1/20:3);TG(14:0/20:1/22:5);TG(18:1/20:1/20:2);TG(14:0/18:4/20:1);FFA(4:0);TG(16:0/20:4/22:2) |
| Regulation of lipolysis in adipocytes | 0.074540684 | TG(14:0/20:0/22:3);TG(14:0/20:5/22:4);TG(16:0/16:0/18:1);TG(16:0/18:3/22:5);FFA(6:0);FFA(20:0);TG(16:1/16:1/20:5);TG(18:2/18:3/20:2);TG(16:0/18:0/22:0);FFA(17:0);TG(16:1/20:2/22:0);TG(14:0/20:4/22:0);TG(14:0/16:1/22:6);TG(14:0/18:0/18:0);TG(14:0/20:4/22:4);FFA(18:0);TG(16:0/16:1/20:4);TG(18:2/18:3/20:3);TG(18:0/18:3/20:3);FFA(22:2);TG(16:0/16:1/18:4);TG(14:0/18:2/18:3);TG(14:0/18:3/22:3);TG(16:0/20:0/22:0);FFA(4:0);TG(16:0/20:4/22:2);TG(18:1/20:1/20:2);TG(14:0/20:1/22:5);TG(14:0/18:4/20:1);TG(14:0/22:4/22:4);TG(14:0/20:1/20:3);TG(12:0/18:1/18:3);TG(16:0/16:1/20:5);TG(14:0/22:1/22:2);TG(14:0/22:0/22:2);TG(16:0/16:1/22:5) |
| Vitamin digestion and absorption | 0.074540684 | TG(14:0/20:4/22:0);TG(18:2/18:3/20:2);TG(16:1/16:1/20:5);FFA(17:0);TG(16:1/20:2/22:0);TG(16:0/18:0/22:0);TG(18:2/18:3/20:3);TG(14:0/18:0/18:0);TG(14:0/16:1/22:6);TG(16:0/16:1/20:4);FFA(18:0);TG(14:0/20:4/22:4);TG(14:0/20:5/22:4);TG(14:0/20:0/22:3);FFA(20:0);TG(16:0/16:0/18:1);TG(16:0/18:3/22:5);FFA(6:0);TG(14:0/20:1/22:5);TG(18:1/20:1/20:2);TG(14:0/18:4/20:1);FFA(4:0);TG(16:0/20:4/22:2);TG(14:0/22:0/22:2);TG(14:0/22:1/22:2);TG(16:0/16:1/20:5);TG(16:0/16:1/22:5);TG(14:0/20:1/20:3);TG(12:0/18:1/18:3);TG(14:0/22:4/22:4);TG(16:0/16:1/18:4);FFA(22:2);TG(18:0/18:3/20:3);TG(14:0/18:3/22:3);TG(16:0/20:0/22:0);TG(14:0/18:2/18:3) |
| Insulin resistance | 0.09972576 | FFA(4:0);TG(16:0/20:4/22:2);TG(14:0/20:1/22:5);TG(18:1/20:1/20:2);TG(14:0/18:4/20:1);TG(14:0/22:4/22:4);TG(14:0/20:1/20:3);TG(12:0/18:1/18:3);TG(16:0/16:1/20:5);TG(14:0/22:0/22:2);TG(14:0/22:1/22:2);TG(16:0/16:1/22:5);TG(18:0/18:3/20:3);FFA(22:2);TG(16:0/16:1/18:4);TG(14:0/18:2/18:3);TG(14:0/18:3/22:3);TG(16:0/20:0/22:0);TG(16:1/16:1/20:5);TG(18:2/18:3/20:2);TG(16:0/18:0/22:0);TG(16:1/20:2/22:0);FFA(17:0);TG(14:0/20:4/22:0);TG(14:0/16:1/22:6);TG(14:0/18:0/18:0);TG(14:0/20:4/22:4);FFA(18:0);TG(16:0/16:1/20:4);TG(18:2/18:3/20:3);TG(14:0/20:0/22:3);TG(14:0/20:5/22:4);CER(D18:0/16:0);TG(16:0/16:0/18:1);FFA(6:0);TG(16:0/18:3/22:5);FFA(20:0) |
| Fat digestion and absorption | 0.128492097 | TG(16:1/16:1/20:5);TG(18:2/18:3/20:2);TG(16:0/18:0/22:0);FFA(17:0);TG(16:1/20:2/22:0);TG(14:0/20:4/22:0)  ;TG(14:0/16:1/22:6);TG(14:0/18:0/18:0);TG(14:0/20:4/22:4);FFA(18:0);TG(16:0/16:1/20:4);TG(18:2/18:3/20:3);  TG(14:0/20:0/22:3);TG(14:0/20:5/22:4);TG(16:0/16:0/18:1);TG(16:0/18:3/22:5);FFA(6:0);FFA(20:0);FFA(4:0);TG(16:0/20:4/22:2);TG(14:0/20:1/22:5);TG(18:1/20:1/20:2);TG(14:0/18:4/20:1);TG(14:0/22:4/22:4)  ;TG(14:0/20:1/20:3);TG(12:0/18:1/18:3);TG(16:0/16:1/20:5);TG(14:0/22:1/22:2);TG(14:0/22:0/22:2);TG(16:0/16:1/22:5);  TG(18:0/18:3/20:3);FFA(22:2);TG(16:0/16:1/18:4);TG(14:0/18:2/18:3);TG(14:0/18:3/22:3);TG(16:0/20:0/22:0) |
| Salmonella infection | 0.133984952 | PI(18:0/20:5);PI(20:4/16:0) |
| Systemic lupus erythematosus | 0.133984952 | PS(20:5/18:0);PS(20:4/20:0) |
| Tuberculosis | 0.133984952 | PI(20:4/16:0);PI(18:0/20:5) |
| Glycine, serine and threonine metabolism | 0.133984952 | PS(20:5/18:0);PS(20:4/20:0) |
| Amoebiasis | 0.184120376 | PS(20:4/20:0);PS(20:5/18:0) |
| Non-alcoholic fatty liver disease (NAFLD) | 0.219764461 | FFA(6:0);FFA(18:0);FFA(20:0);FFA(17:0);FFA(22:2);FFA(4:0) |
| Fatty acid elongation | 0.219764461 | FFA(18:0);FFA(6:0);FFA(20:0);FFA(4:0);FFA(22:2);FFA(17:0) |
| Type II diabetes mellitus | 0.219764461 | FFA(4:0);FFA(22:2);FFA(17:0);FFA(6:0);FFA(18:0);FFA(20:0) |
| AMPK signaling pathway | 0.219764461 | FFA(18:0);FFA(6:0);FFA(20:0);FFA(22:2);FFA(17:0);FFA(4:0) |
| Fatty acid degradation | 0.219764461 | FFA(20:0);FFA(18:0);FFA(6:0);FFA(4:0);FFA(22:2);FFA(17:0) |
| Insulin secretion | 0.219764461 | FFA(22:2);FFA(17:0);FFA(4:0);FFA(20:0);FFA(18:0);FFA(6:0) |
| Glycerolipid metabolism | 0.295966606 | DG(16:0/20:1/0:0);FFA(20:0);TG(16:0/18:3/22:5);FFA(6:0);TG(16:0/16:0/18:1);TG(14:0/20:5/22:4);DG(18:1/22:0/0:0);TG(14:0/20:0/22:3);TG(18:2/18:3/20:3);TG(14:0/20:4/22:4);DG(18:4/18:1/0:0);FFA(18:0);TG(16:0/16:1/20:4);TG(14:0/16:1/22:6);DG(18:2/22:4/0:0);TG(14:0/18:0/18:0);TG(14:0/20:4/22:0);DG(16:0/18:3/0:0);TG(16:0/18:0/22:0);FFA(17:0);TG(16:1/20:2/22:0);TG(16:1/16:1/20:5);TG(18:2/18:3/20:2);TG(16:0/20:0/22:0);TG(14:0/18:3/22:3);TG(14:0/18:2/18:3);TG(16:0/16:1/18:4);TG(18:0/18:3/20:3);FFA(22:2);TG(16:0/16:1/22:5);TG(16:0/16:1/20:5);TG(14:0/22:0/22:2);TG(14:0/22:1/22:2);TG(14:0/22:4/22:4);TG(12:0/18:1/18:3);TG(14:0/20:1/20:3);TG(14:0/18:4/20:1);TG(18:1/20:1/20:2);TG(14:0/20:1/22:5);TG(16:0/20:4/22:2);FFA(4:0) |
| Metabolic pathways | 0.390392119 | PS(20:5/18:0);PI(20:4/16:0);PE(20:3/18:2);PE(22:2/14:0);SM(D18:0/16:0);TG(14:0/22:4/22:4);TG(12:0/18:1/18:3);PE(20:3/20:4);TG(14:0/22:0/22:2);TG(14:0/22:1/22:2);TG(14:0/20:0/22:3);PC(18:1/18:4);TG(14:0/20:5/22:4);SM(D18:0/18:1);PE(18:1/20:3);PI(18:0/20:5);DG(16:0/18:3/0:0);PE(16:1/22:0);PC(16:0/20:3);TG(16:1/16:1/20:5);TG(18:2/18:3/20:2);TG(14:0/20:4/22:0);PC(22:4/18:1);PS(20:4/20:0);PC(18:2/20:3);PE(18:3/18:1);DG(18:2/22:4/0:0);TG(14:0/18:0/18:0);TG(18:0/18:3/20:3);PE(16:0/20:5);PE(20:1/20:5);TG(16:0/16:1/18:4);TG(14:0/18:2/18:3);PC(18:0/14:0);TG(16:0/20:0/22:0);PE(22:2/12:0);PC(18:2/20:4);PC(20:0/22:6);TG(14:0/18:3/22:3);TG(16:0/20:4/22:2);TG(14:0/18:4/20:1);TG(18:1/20:1/20:2);TG(14:0/20:1/22:5);PC(20:1/22:1);TG(14:0/20:1/20:3);PC(20:5/18:0);TG(16:0/16:1/22:5);TG(16:0/16:1/20:5);DG(18:1/22:0/0:0);PE(16:0/14:0);TG(16:0/18:3/22:5);CER(D18:0/16:0);TG(16:0/16:0/18:1);DG(16:0/20:1/0:0);TG(16:0/18:0/22:0);TG(16:1/20:2/22:0);PC(18:0/14:1);TG(14:0/20:4/22:4);TG(16:0/16:1/20:4);PC(18:0/12:0);DG(18:4/18:1/0:0);TG(14:0/16:1/22:6);SM(D18:0/18:0);PE(20:0/18:0);TG(18:2/18:3/20:3) |
| alpha-Linolenic acid metabolism | 0.394672734 | PC(20:5/18:0);PC(18:2/20:4);PC(20:0/22:6);PC(18:0/12:0);PC(20:1/22:1);PC(18:2/20:3);PC(18:0/14:0);PC(18:1/18:4);PC(22:4/18:1);PC(18:0/14:1);PC(16:0/20:3) |
| Autophagy - other | 0.402748077 | PE(22:2/14:0);PE(20:3/18:2);PE(16:1/22:0);PE(18:3/18:1);PE(20:0/18:0);PE(20:3/20:4);PE(16:0/14:0);PE(16:0/20:5);PE(20:1/20:5);PE(18:1/20:3);PE(22:2/12:0);PI(20:4/16:0);PI(18:0/20:5) |
| Glycosylphosphatidylinositol (GPI)-anchor biosynthesis | 0.402748077 | PE(16:1/22:0);PE(20:3/18:2);PE(22:2/14:0);PE(20:3/20:4);PE(20:0/18:0);PE(18:3/18:1);PE(20:1/20:5);PE(16:0/20:5);PE(16:0/14:0);PI(18:0/20:5);PI(20:4/16:0);PE(22:2/12:0);PE(18:1/20:3) |
| Autophagy - animal | 0.402748077 | PE(16:0/20:5);PE(20:1/20:5);PE(16:0/14:0);PI(20:4/16:0);PI(18:0/20:5);PE(22:2/12:0);PE(18:1/20:3);PE(20:3/18:2);PE(16:1/22:0);PE(22:2/14:0);PE(20:3/20:4);PE(20:0/18:0);PE(18:3/18:1) |
| Linoleic acid metabolism | 0.413883571 | PC(18:2/20:3);PC(20:1/22:1);PC(18:0/12:0);PC(18:0/14:0);PC(20:5/18:0);PC(18:2/20:4);PC(20:0/22:6);PC(16:0/20:3);PC(18:1/18:4);PC(22:4/18:1);PC(18:0/14:1) |
| Adipocytokine signaling pathway | 0.41533461 | FFA(17:0);FFA(22:2);FFA(4:0);FFA(20:0);FFA(6:0);FFA(18:0);CER(D18:0/16:0) |
| Arachidonic acid metabolism | 0.433144396 | PC(16:0/20:3);PC(22:4/18:1);PC(18:1/18:4);PC(18:0/14:1);PC(18:2/20:3);PC(20:1/22:1);PC(18:0/12:0);PC(18:0/14:0);PC(20:5/18:0);PC(20:0/22:6);PC(18:2/20:4) |
| Retrograde endocannabinoid signaling | 0.459150738 | PE(22:2/12:0);PC(18:2/20:4);PC(20:0/22:6);PC(18:0/14:0);PE(18:1/20:3);PE(16:0/20:5);PE(20:1/20:5);PC(18:1/18:4);PE(16:0/14:0);PE(20:0/18:0);PE(20:3/20:4);PC(20:5/18:0);PE(18:3/18:1);PC(18:2/20:3);PC(20:1/22:1);PC(18:0/12:0);PC(18:0/14:1);PC(22:4/18:1);PC(16:0/20:3);PE(22:2/14:0);PE(20:3/18:2);PE(16:1/22:0) |
| Choline metabolism in cancer | 0.533503062 | PC(22:4/18:1);PC(18:0/14:1);FFA(17:0);PC(16:0/20:3);FFA(4:0);PC(20:5/18:0);FFA(18:0);PC(20:1/22:1);PC(18:2/20:3);PC(18:0/12:0);PC(18:1/18:4);FFA(22:2);LPC(18:3/0:0);PC(18:2/20:4);FFA(20:0);PC(20:0/22:6);FFA(6:0);PC(18:0/14:0) |
| Leishmaniasis | 0.54692448 | PS(20:5/18:0);CER(D18:0/16:0);PS(20:4/20:0) |
| Kaposi sarcoma-associated herpesvirus infection | 0.583899147 | PE(20:3/20:4);PE(20:0/18:0);PE(22:2/12:0);PE(18:3/18:1);PE(18:1/20:3);PE(16:0/20:5);PE(20:1/20:5);PE(20:3/18:2);PE(16:1/22:0);PE(16:0/14:0);PE(22:2/14:0) |
| Pathogenic Escherichia coli infection | 0.601752878 | PE(18:1/20:3);PE(18:3/18:1);PE(20:0/18:0);PE(22:2/12:0);PE(20:3/20:4);PE(22:2/14:0);PE(16:0/14:0);PE(16:1/22:0);PE(20:3/18:2);PE(20:1/20:5);PE(16:0/20:5) |
| Inositol phosphate metabolism | 0.717245278 | DG(18:1/22:0/0:0);DG(16:0/18:3/0:0);DG(18:4/18:1/0:0);DG(18:2/22:4/0:0);PI(20:4/16:0);PI(18:0/20:5);DG(16:0/20:1/0:0) |
| Sphingolipid signaling pathway | 0.808590541 | CER(D18:0/16:0);SM(D18:0/18:1);SM(D18:0/16:0);SM(D18:0/18:0) |
| Sphingolipid metabolism | 0.808590541 | SM(D18:0/18:1);CER(D18:0/16:0);SM(D18:0/18:0);SM(D18:0/16:0) |
| Necroptosis | 0.824467323 | SM(D18:0/16:0);SM(D18:0/18:0);CER(D18:0/16:0);SM(D18:0/18:1) |
| Phosphatidylinositol signaling system | 0.846860176 | PI(18:0/20:5);DG(16:0/20:1/0:0);PI(20:4/16:0);DG(18:4/18:1/0:0);DG(18:1/22:0/0:0);DG(16:0/18:3/0:0);DG(18:2/22:4/0:0) |
| AGE-RAGE signaling pathway in diabetic complications | 0.886326574 | CER(D18:0/16:0) |
| Neurotrophin signaling pathway | 0.886326574 | CER(D18:0/16:0) |
| Long-term depression | 0.895550969 | DG(16:0/18:3/0:0);DG(18:1/22:0/0:0);DG(18:4/18:1/0:0);DG(18:2/22:4/0:0);DG(16:0/20:1/0:0) |
| Glycerophospholipid metabolism | 0.898721548 | PS(20:5/18:0);PC(18:0/14:0);PI(20:4/16:0);PE(22:2/12:0);PC(18:2/20:4);PC(20:0/22:6);LPC(18:3/0:0);PE(16:0/20:5);PE(20:1/20:5);PC(20:1/22:1);PE(20:3/20:4);PC(20:5/18:0);PE(20:3/18:2);PE(22:2/14:0);PE(18:1/20:3);DG(16:0/20:1/0:0);PI(18:0/20:5);DG(18:1/22:0/0:0);PE(16:0/14:0);PC(18:1/18:4);PS(20:4/20:0);PC(18:0/12:0);DG(18:4/18:1/0:0);PC(18:2/20:3);DG(18:2/22:4/0:0);PE(18:3/18:1);PE(20:0/18:0);DG(16:0/18:3/0:0);PE(16:1/22:0);PC(16:0/20:3);LPE(0:0/22:5);PC(22:4/18:1);PC(18:0/14:1) |
| **Comparison III: LC vs. LT comparison** | | |
| Insulin secretion | 0.000509019 | FFA(20:2);FFA(6:0);FFA(22:1);FFA(24:6);FFA(22:3);FFA(14:0);FFA(17:0);FFA(4:0);FFA(18:0);FFA(24:0);FFA(22:2);FFA(13:0) |
| Type II diabetes mellitus | 0.000509019 | FFA(24:0);FFA(18:0);FFA(4:0);FFA(22:2);FFA(13:0);FFA(20:2);FFA(24:6);FFA(22:1);FFA(6:0);FFA(17:0);FFA(22:3);FFA(14:0) |
| Non-alcoholic fatty liver disease (NAFLD) | 0.000509019 | FFA(4:0);FFA(18:0);FFA(24:0);FFA(22:2);FFA(13:0);FFA(20:2);FFA(6:0);FFA(22:1);FFA(24:6);FFA(14:0);FFA(22:3);FFA(17:0) |
| Fatty acid degradation | 0.000509019 | FFA(13:0);FFA(22:2);FFA(18:0);FFA(24:0);FFA(4:0);FFA(17:0);FFA(14:0);FFA(22:3);FFA(24:6);FFA(6:0);FFA(22:1);FFA(20:2) |
| AMPK signaling pathway | 0.000509019 | FFA(13:0);FFA(22:2);FFA(18:0);FFA(24:0);FFA(4:0);FFA(24:6);FFA(6:0);FFA(22:1);FFA(20:2);FFA(17:0);FFA(14:0);FFA(22:3) |
| Fatty acid elongation | 0.000509019 | FFA(13:0);FFA(22:2);FFA(4:0);FFA(18:0);FFA(24:0);FFA(6:0);FFA(22:1);FFA(24:6);FFA(20:2);FFA(22:3);FFA(14:0);FFA(17:0) |
| Adipocytokine signaling pathway | 0.002752792 | CER(D18:1/22:0);FFA(13:0);FFA(22:2);FFA(24:0);FFA(18:0);FFA(4:0);FFA(17:0);FFA(22:3);FFA(14:0);CER(D18:1/24:0);FFA(24:6);FFA(22:1);FFA(6:0);FFA(20:2) |
| Choline metabolism in cancer | 0.005846328 | PC(18:0/22:0);FFA(17:0);PA(18:0/18:1);PC(18:2/18:2);FFA(22:1);PC(20:4/20:4);PC(18:1/22:6);PA(16:0/18:0);PC(20:5/12:0);FFA(22:2);FFA(14:0);FFA(22:3);PC(20:1/20:4);LPC(18:3/0:0);PC(16:0/20:3);FFA(6:0);FFA(24:6);PC(18:2/20:3);FFA(20:2);PC(22:2/18:1);PC(18:2/20:4);PC(18:1/18:4);FFA(13:0);PA(16:0/20:1);PC(16:1/18:3);FFA(4:0);PC(20:1/14:1);FFA(24:0);FFA(18:0) |
| Salmonella infection | 0.023260643 | PI(20:3/18:0);PI(18:1/20:4);PI(20:4/16:0) |
| Tuberculosis | 0.023260643 | PI(18:1/20:4);PI(20:3/18:0);PI(20:4/16:0) |
| Phosphatidylinositol signaling system | 0.099416163 | PA(18:0/18:1);DG(16:0/20:0/0:0);DG(14:0/18:2/0:0);PA(16:0/18:0);PI(20:3/18:0);DG(12:0/22:0/0:0);DG(16:0/18:0/0:0);PI(20:4/16:0);DG(20:0/18:2/0:0);DG(14:0/20:0/0:0);DG(16:0/18:3/0:0);PI(18:1/20:4);DG(16:1/22:0/0:0);PA(16:0/20:1) |
| Pancreatic cancer | 0.126987759 | PA(16:0/18:0);PA(16:0/20:1);PA(18:0/18:1) |
| cAMP signaling pathway | 0.126987759 | PA(16:0/20:1);PA(16:0/18:0);PA(18:0/18:1) |
| Systemic lupus erythematosus | 0.153427777 | PS(18:0/22:6);PS(20:4/20:0) |
| Glycine, serine and threonine metabolism | 0.153427777 | PS(18:0/22:6);PS(20:4/20:0) |
| Pathways in cancer | 0.163233209 | PA(18:0/18:1);PA(16:0/20:1);PA(16:0/18:0) |
| Phospholipase D signaling pathway | 0.163233209 | PA(16:0/20:1);PA(16:0/18:0);PA(18:0/18:1) |
| Fc gamma R-mediated phagocytosis | 0.163233209 | PA(18:0/18:1);PA(16:0/20:1);PA(16:0/18:0) |
| GnRH signaling pathway | 0.163233209 | PA(16:0/20:1);PA(16:0/18:0);PA(18:0/18:1) |
| Arachidonic acid metabolism | 0.183573167 | PC(16:1/18:3);PC(20:1/14:1);15OXOETE;PC(18:1/22:6);PC(22:2/18:1);PC(20:5/12:0);PC(18:2/20:4);PC(18:1/18:4);PC(20:4/20:4);PC(18:2/20:3);PC(20:1/20:4);PC(18:0/22:0);PC(16:0/20:3);PC(18:2/18:2) |
| Amoebiasis | 0.209282182 | PS(20:4/20:0);PS(18:0/22:6) |
| Inositol phosphate metabolism | 0.230220157 | DG(16:0/18:3/0:0);DG(14:0/20:0/0:0);DG(16:1/22:0/0:0);PI(18:1/20:4);DG(14:0/18:2/0:0);DG(16:0/20:0/0:0);DG(20:0/18:2/0:0);DG(12:0/22:0/0:0);DG(16:0/18:0/0:0);PI(20:4/16:0);PI(20:3/18:0) |
| alpha-Linolenic acid metabolism | 0.251588575 | PC(16:1/18:3);PC(20:1/14:1);PC(18:1/22:6);PC(18:2/20:4);PC(18:1/18:4);PC(20:5/12:0);PC(22:2/18:1);PC(20:4/20:4);PC(18:2/20:3);PC(16:0/20:3);PC(18:0/22:0);PC(20:1/20:4);PC(18:2/18:2) |
| Linoleic acid metabolism | 0.268745891 | PC(16:1/18:3);PC(20:1/14:1);PC(18:1/22:6);PC(22:2/18:1);PC(20:5/12:0);PC(18:2/20:4);PC(18:1/18:4);PC(20:4/20:4);PC(18:2/20:3);PC(18:0/22:0);PC(20:1/20:4);PC(16:0/20:3);PC(18:2/18:2) |
| Glycerolipid metabolism | 0.315181502 | FFA(17:0);TG(18:2/18:3/20:0);PA(18:0/18:1);TG(16:0/20:4/22:6);TG(14:0/22:0/22:2);TG(18:1/18:3/20:1);DG(14:0/18:2/0:0);FFA(22:2);TG(16:0/20:0/22:0);TG(18:1/18:2/20:0);TG(14:0/20:1/22:5);TG(14:0/20:1/22:2);FFA(14:0);DG(16:0/20:0/0:0);TG(14:0/20:3/22:4);TG(14:0/14:1/18:1);TG(14:0/20:4/22:3);FFA(6:0);DG(16:0/18:3/0:0);TG(14:0/18:1/20:3);PA(16:0/20:1);FFA(18:0);TG(16:1/18:1/18:4);TG(14:0/20:1/20:3);TG(18:1/18:2/18:3);TG(16:1/16:1/22:5);FFA(22:1);PA(16:0/18:0);DG(16:0/18:0/0:0);TG(18:2/18:2/22:0);DG(20:0/18:2/0:0);TG(14:0/18:3/22:3);DG(14:0/20:0/0:0);DG(12:0/22:0/0:0);FFA(22:3);TG(14:0/16:0/22:0);FFA(24:6);DG(16:1/22:0/0:0);FFA(20:2);TG(16:0/16:1/22:1);TG(18:2/18:3/20:1);FFA(13:0);FFA(24:0);FFA(4:0) |
| Fat digestion and absorption | 0.318057981 | TG(16:0/20:4/22:6);TG(16:1/16:1/22:5);FFA(22:1);TG(18:1/18:2/18:3);PA(18:0/18:1);FFA(17:0);TG(14:0/20:1/20:3);TG(18:2/18:3/20:0);TG(18:1/18:2/20:0);TG(16:0/20:0/22:0);FFA(22:2);TG(14:0/18:3/22:3);TG(18:1/18:3/20:1);TG(14:0/22:0/22:2);PA(16:0/18:0);TG(18:2/18:2/22:0);FFA(20:2);TG(16:0/16:1/22:1);FFA(24:6);TG(14:0/14:1/18:1);FFA(6:0);TG(14:0/20:4/22:3);TG(14:0/16:0/22:0);TG(14:0/20:3/22:4);TG(14:0/20:1/22:5);FFA(14:0);FFA(22:3);TG(14:0/20:1/22:2);FFA(18:0);FFA(24:0);TG(16:1/18:1/18:4);FFA(4:0);PA(16:0/20:1);TG(14:0/18:1/20:3);FFA(13:0);TG(18:2/18:3/20:1) |
| Glycerophospholipid metabolism | 0.326929971 | DG(16:0/18:3/0:0);PC(20:1/20:4);PG(18:1/18:1);DG(16:0/20:0/0:0);PC(16:1/18:3);PA(16:0/20:1);PC(18:2/20:4);PC(22:2/18:1);PE(22:2/12:0);PG(18:0/18:1);PI(20:3/18:0);PE(22:1/20:3);LPE(0:0/18:0);PA(18:0/18:1);PS(18:0/22:6);PC(18:1/22:6);PE(18:2/16:0);PC(20:5/12:0);DG(14:0/18:2/0:0);DG(16:1/22:0/0:0);PC(18:2/20:3);DG(12:0/22:0/0:0);PC(16:0/20:3);LPC(18:3/0:0);PI(18:1/20:4);PC(20:1/14:1);PE(24:0/18:1);PC(18:1/18:4);PE(16:0/14:0);PE(20:4/14:0);PC(20:4/20:4);PE(20:2/16:0);PC(18:0/22:0);PC(18:2/18:2);PG(18:1/20:4);LPE(0:0/22:4);DG(14:0/20:0/0:0);PS(20:4/20:0);PA(16:0/18:0);DG(16:0/18:0/0:0);PI(20:4/16:0);DG(20:0/18:2/0:0);PE(20:5/18:1) |
| Leishmaniasis | 0.358369241 | CER(D18:1/24:0);CER(D18:1/22:0);PS(18:0/22:6);PS(20:4/20:0) |
| Cholesterol metabolism | 0.434770226 | TG(16:1/16:1/22:5);FFA(22:1);TG(16:0/20:4/22:6);FFA(17:0);TG(14:0/20:1/20:3);TG(18:2/18:3/20:0);TG(18:1/18:2/18:3);TG(16:0/20:0/22:0);FFA(22:2);TG(18:1/18:2/20:0);TG(14:0/22:0/22:2);TG(18:2/18:2/22:0);TG(14:0/18:3/22:3);TG(18:1/18:3/20:1);FFA(24:6);TG(14:0/14:1/18:1);FFA(6:0);TG(14:0/20:4/22:3);FFA(20:2);TG(16:0/16:1/22:1);TG(14:0/20:1/22:5);FFA(14:0);FFA(22:3);TG(14:0/20:1/22:2);TG(14:0/16:0/22:0);TG(14:0/20:3/22:4);TG(14:0/18:1/20:3);FFA(13:0);FFA(18:0);FFA(24:0);TG(16:1/18:1/18:4);FFA(4:0);TG(18:2/18:3/20:1) |
| Insulin resistance | 0.451710943 | TG(16:0/16:1/22:1);FFA(20:2);TG(14:0/20:4/22:3);FFA(6:0);CER(D18:1/24:0);TG(14:0/14:1/18:1);FFA(24:6);TG(14:0/20:3/22:4);TG(14:0/16:0/22:0);TG(14:0/20:1/22:2);FFA(14:0);FFA(22:3);TG(14:0/20:1/22:5);FFA(4:0);TG(16:1/18:1/18:4);FFA(24:0);FFA(18:0);FFA(13:0);TG(14:0/18:1/20:3);TG(18:2/18:3/20:1);TG(16:0/20:4/22:6);FFA(22:1);TG(16:1/16:1/22:5);TG(18:1/18:2/18:3);TG(18:2/18:3/20:0);FFA(17:0);TG(14:0/20:1/20:3);TG(18:1/18:2/20:0);FFA(22:2);TG(16:0/20:0/22:0);TG(18:1/18:3/20:1);TG(14:0/18:3/22:3);CER(D18:1/22:0);TG(18:2/18:2/22:0);TG(14:0/22:0/22:2) |
| Thermogenesis | 0.463121019 | TG(18:2/18:2/22:0);TG(14:0/22:0/22:2);TG(18:1/18:3/20:1);TG(14:0/18:3/22:3);FFA(22:2);TG(16:0/20:0/22:0);TG(18:1/18:2/20:0);TG(18:2/18:3/20:0);FFA(17:0);TG(14:0/20:1/20:3);TG(18:1/18:2/18:3);FFA(22:1);TG(16:1/16:1/22:5);TG(16:0/20:4/22:6);TG(18:2/18:3/20:1);FFA(13:0);TG(14:0/18:1/20:3);FFA(4:0);TG(16:1/18:1/18:4);FFA(24:0);FFA(18:0);TG(14:0/20:1/22:2);FFA(22:3);FFA(14:0);TG(14:0/20:1/22:5);TG(14:0/20:3/22:4);TG(14:0/16:0/22:0);TG(14:0/20:4/22:3);FFA(6:0);TG(14:0/14:1/18:1);FFA(24:6);TG(16:0/16:1/22:1);FFA(20:2) |
| Regulation of lipolysis in adipocytes | 0.477354029 | TG(18:2/18:2/22:0);TG(14:0/22:0/22:2);TG(18:1/18:3/20:1);TG(14:0/18:3/22:3);FFA(22:2);TG(16:0/20:0/22:0);TG(18:1/18:2/20:0);TG(18:2/18:3/20:0);FFA(17:0);TG(14:0/20:1/20:3);TG(18:1/18:2/18:3);FFA(22:1);TG(16:1/16:1/22:5);TG(16:0/20:4/22:6);TG(18:2/18:3/20:1);TG(14:0/18:1/20:3);FFA(13:0);FFA(4:0);TG(16:1/18:1/18:4);FFA(24:0);FFA(18:0);FFA(22:3);TG(14:0/20:1/22:2);FFA(14:0);TG(14:0/20:1/22:5);TG(14:0/20:3/22:4);TG(14:0/16:0/22:0);TG(14:0/20:4/22:3);FFA(6:0);TG(14:0/14:1/18:1);FFA(24:6);TG(16:0/16:1/22:1);FFA(20:2) |
| Vitamin digestion and absorption | 0.477354029 | TG(18:1/18:2/18:3);TG(18:2/18:3/20:0);FFA(17:0);TG(14:0/20:1/20:3);TG(16:0/20:4/22:6);FFA(22:1);TG(16:1/16:1/22:5);TG(18:1/18:3/20:1);TG(14:0/18:3/22:3);TG(18:2/18:2/22:0);TG(14:0/22:0/22:2);TG(18:1/18:2/20:0);TG(16:0/20:0/22:0);FFA(22:2);TG(14:0/20:3/22:4);TG(14:0/16:0/22:0);TG(14:0/20:1/22:2);FFA(14:0);FFA(22:3);TG(14:0/20:1/22:5);TG(16:0/16:1/22:1)  ;FFA(20:2);FFA(6:0);TG(14:0/20:4/22:3);FFA(24:6);TG(14:0/14:1/18:1);TG(18:2/18:3/20:1);TG(16:1/18:1/18:4);FFA(4:0);FFA(18:0);FFA(24:0);TG(14:0/18:1/20:3);FFA(13:0) |
| Long-term depression | 0.576412969 | DG(16:0/18:3/0:0);DG(14:0/20:0/0:0);DG(16:1/22:0/0:0);DG(14:0/18:2/0:0);DG(16:0/20:0/0:0);DG(20:0/18:2/0:0);DG(16:0/18:0/0:0);DG(12:0/22:0/0:0) |
| AGE-RAGE signaling pathway in diabetic complications | 0.6623817 | CER(D18:1/24:0);CER(D18:1/22:0) |
| Neurotrophin signaling pathway | 0.6623817 | CER(D18:1/22:0);CER(D18:1/24:0) |
| Retrograde endocannabinoid signaling | 0.73914848 |  |
| Glycosylphosphatidylinositol (GPI)-anchor biosynthesis | 0.775483188 | PE(22:1/20:3);PI(20:3/18:0);PE(22:2/12:0);PE(20:2/16:0);PE(24:0/18:1);PE(18:2/16:0);PI(20:4/16:0);PE(20:4/14:0);PE(20:5/18:1);PE(16:0/14:0);PI(18:1/20:4) |
| Autophagy - animal | 0.775483188 | PI(18:1/20:4);PE(20:4/14:0);PE(20:5/18:1);PE(16:0/14:0);PE(18:2/16:0);PE(24:0/18:1);PI(20:4/16:0);PE(20:2/16:0);PE(22:2/12:0);PE(22:1/20:3);PI(20:3/18:0) |
| Autophagy - other | 0.775483188 | PI(18:1/20:4);PE(16:0/14:0);PE(20:4/14:0);PE(20:5/18:1);PI(20:4/16:0);PE(18:2/16:0);PE(24:0/18:1);PE(20:2/16:0);PE(22:2/12:0);PI(20:3/18:0);PE(22:1/20:3) |
| Kaposi sarcoma-associated herpesvirus infection | 0.945933649 | PE(20:2/16:0);PE(22:2/12:0);PE(16:0/14:0);PE(20:5/18:1);PE(20:4/14:0);PE(18:2/16:0);PE(22:1/20:3);PE(24:0/18:1) |
| Pathogenic Escherichia coli infection | 0.950707688 | PE(20:5/18:1);PE(20:4/14:0);PE(16:0/14:0);PE(24:0/18:1);PE(18:2/16:0);PE(22:1/20:3);PE(22:2/12:0);PE(20:2/16:0) |
| Metabolic pathways | 0.987005889 | TG(14:0/20:1/20:3);PC(18:0/22:0);TG(18:1/18:2/18:3);PC(18:2/18:2);PC(20:4/20:4);TG(16:1/16:1/22:5);PE(20:2/16:0);  PA(16:0/18:0);PI(20:4/16:0);DG(16:0/18:0/0:0);TG(18:2/18:2/22:0);DG(20:0/18:2/0:0);TG(14:0/18:3/22:3);PE(20:5/18:1);PG(18:1/20:4);DG(14:0/20:0/0:0);PS(20:4/20:0);PC(16:0/20:3);DG(12:0/22:0/0:0);TG(14:0/16:0/22:0);DG(16:1/22:0/0:0);TG(16:0/16:1/22:1);PC(18:2/20:3);TG(18:2/18:3/20:1);PE(24:0/18:1);PC(18:1/18:4);PE(16:0/14:0);PE(20:4/14:0);PI(18:1/20:4);PC(20:1/14:1);PI(20:3/18:0);TG(18:2/18:3/20:0);PE(22:1/20:3);PA(18:0/18:1);TG(16:0/20:4/22:6);PE(22:2/12:0);PG(18:0/18:1);TG(14:0/22:0/22:2);PC(18:1/22:6);PE(18:2/16:0);CER(D18:1/22:0);PC(20:5/12:0);TG(18:1/18:3/20:1);DG(14:0/18:2/0:0);TG(16:0/20:0/22:0);PS(18:0/22:6);TG(18:1/18:2/20:0);TG(14:0/20:1/22:5);TG(14:0/20:1/22:2);PC(20:1/20:4);PG(18:1/18:1);DG(16:0/20:0/0:0);TG(14:0/20:3/22:4);TG(14:0/14:1/18:1);CER(D18:1/24:0);TG(14:0/20:4/22:3);DG(16:0/18:3/0:0);PC(18:2/20:4);PC(22:2/18:1);PC(16:1/18:3);TG(14:0/18:1/20:3);PA(16:0/20:1);TG(16:1/18:1/18:4) |
| Sphingolipid signaling pathway | 0.987193572 | CER(D18:1/22:0);CER(D18:1/24:0) |
| Sphingolipid metabolism | 0.987193572 | CER(D18:1/24:0);CER(D18:1/22:0) |
| Necroptosis | 0.98892439 | CER(D18:1/24:0);CER(D18:1/22:0) |
| **LC vs. LF comparison** | | |
| Insulin secretion | 0.00063685 | FFA(22:3);FFA(24:6);FFA(22:2);FFA(22:4);FFA(16:1);FFA(24:5);FFA(12:0);FFA(24:4);FFA(8:0);FFA(22:1);FFA(4:0);FFA(20:2);FFA(17:1) |
| AMPK signaling pathway | 0.00063685 | FFA(24:5);FFA(16:1);FFA(24:4);FFA(12:0);FFA(22:1);FFA(8:0);FFA(4:0);FFA(17:1);FFA(20:2);FFA(24:6);FFA(22:3);FFA(22:4);FFA(22:2) |
| Type II diabetes mellitus | 0.00063685 | FFA(17:1);FFA(20:2);FFA(22:1);FFA(8:0);FFA(4:0);FFA(24:4);FFA(12:0);FFA(24:5);FFA(16:1);FFA(22:4);FFA(22:2);FFA(24:6);FFA(22:3) |
| Fatty acid elongation | 0.00063685 | FFA(12:0);FFA(24:4);FFA(16:1);FFA(24:5);FFA(20:2);FFA(17:1);FFA(22:1);FFA(8:0);FFA(4:0);FFA(24:6);FFA(22:3);FFA(22:2);FFA(22:4) |
| Fatty acid degradation | 0.00063685 | FFA(22:4);FFA(22:2);FFA(22:3);FFA(24:6);FFA(20:2);FFA(17:1);FFA(4:0);FFA(22:1);FFA(8:0);FFA(12:0);FFA(24:4);FFA(16:1);FFA(24:5) |
| Non-alcoholic fatty liver disease (NAFLD) | 0.00063685 | FFA(20:2);FFA(17:1);FFA(22:1);FFA(4:0);FFA(8:0);FFA(12:0);FFA(24:4);FFA(16:1);FFA(24:5);FFA(22:4);FFA(22:2);FFA(22:3);FFA(24:6) |
| Choline metabolism in cancer | 0.001286101 | LPC(22:4/0:0);FFA(16:1);FFA(24:4);LPC(14:0/0:0);FFA(22:1);LPC(12:0/0:0);LPC(18:0/0:0);PC(18:2/20:3);FFA(17:1);FFA(20:2);PC(16:0/20:3);PC(16:0/20:5);LPC(16:0/0:0);LPC(16:1/0:0);PC(20:5/12:0);PC(20:1/18:2);PC(14:0/18:2);PC(18:1/18:4);FFA(24:5);PC(20:3/18:1);PC(18:0/20:3);FFA(12:0);LPC(18:3/0:0);FFA(8:0);PC(22:4/18:1);FFA(4:0);LPC(18:2/0:0);PC(18:0/22:0);FFA(22:3);FFA(24:6);LPC(20:4/0:0);PC(20:1/20:4);PC(18:1/20:5);FFA(22:2);FFA(22:4) |
| Adipocytokine signaling pathway | 0.001706771 | FFA(17:1);CER(D18:1/22:1);FFA(20:2);FFA(4:0);FFA(22:1);FFA(8:0);FFA(12:0);FFA(24:4);CER(D18:1/26:1);CER(D18:1/22:0);FFA(24:5);FFA(16:1);FFA(22:2);FFA(22:4);FFA(22:3);FFA(24:6) |
| Tuberculosis | 0.003413864 | PI(18:0/20:5);PI(20:3/18:0);PI(18:1/20:4);PI(20:4/16:0) |
| Salmonella infection | 0.003413864 | PI(18:1/20:4);PI(20:4/16:0);PI(20:3/18:0);PI(18:0/20:5) |
| Glycerophospholipid metabolism | 0.013772438 | LPC(18:0/0:0);LPE(0:0/16:0);DG(18:0/18:2/0:0);DG(18:0/18:0/0:0);PE(20:4/14:0);LPC(22:4/0:0);PC(20:1/18:2);PC(20:5/12:0);PE(20:1/20:4);PE(22:2/12:0);PC(18:1/18:4);PC(14:0/18:2);PC(16:0/20:3);LPE(0:0/22:1);PE(16:0/22:4);PC(16:0/20:5);LPC(16:0/0:0);LPC(16:1/0:0);PI(18:0/20:5);LPC(18:2/0:0);DG(16:0/20:1/0:0);PG(18:1/16:1);DG(18:4/18:1/0:0);LPC(18:3/0:0);PC(20:1/20:4);LPC(20:4/0:0);LPE(0:0/22:4);DG(20:0/18:2/0:0);DG(16:1/20:0/0:0);PE(16:0/20:5);PE(16:1/16:0);PE(18:0/14:0);LPE(0:0/24:0);LPC(12:0/0:0);PS(18:0/20:4);PC(18:2/20:3);LPC(14:0/0:0);PE(18:0/20:5);PE(16:0/20:3);PG(18:0/16:0);LPE(0:0/20:0);PE(20:4/22:2);PI(20:4/16:0);PC(22:4/18:1);PE(18:2/16:0);PS(20:4/20:0);PI(18:1/20:4);PC(18:0/20:3);DG(18:2/20:3/0:0);PC(20:3/18:1);PE(18:1/16:1);PC(18:1/20:5);DG(14:0/18:2/0:0);DG(16:0/18:0/0:0);PC(18:0/22:0);PE(16:0/18:0);PG(18:0/18:1);PI(20:3/18:0) |
| Oxidative phosphorylation | 0.169708029 | COENZYME Q10 |
| Ubiquinone and other terpenoid-quinone biosynthesis | 0.169708029 | COENZYME Q10 |
| Inositol phosphate metabolism | 0.196523277 | DG(18:0/18:2/0:0);DG(16:0/20:1/0:0);DG(18:0/18:0/0:0);PI(18:0/20:5);DG(18:4/18:1/0:0);DG(18:2/20:3/0:0);PI(18:1/20:4);DG(20:0/18:2/0:0);DG(16:0/18:0/0:0);DG(16:1/20:0/0:0);DG(14:0/18:2/0:0);PI(20:4/16:0);PI(20:3/18:0) |
| Glycine, serine and threonine metabolism | 0.201557896 | PS(20:4/20:0);PS(18:0/20:4) |
| Systemic lupus erythematosus | 0.201557896 | PS(18:0/20:4);PS(20:4/20:0) |
| Amoebiasis | 0.270231352 | PS(20:4/20:0);PS(18:0/20:4) |
| Leishmaniasis | 0.275490978 | PS(18:0/20:4);PS(20:4/20:0);CER(D18:1/22:0);CER(D18:1/26:1);CER(D18:1/22:1) |
| Glycosylphosphatidylinositol (GPI)-anchor biosynthesis | 0.326675901 | PE(18:0/20:5);PE(18:1/16:1);PE(16:0/20:3);PE(20:4/14:0);PI(18:1/20:4);PI(18:0/20:5);PE(18:2/16:0);PI(20:3/18:0);PE(18:0/14:0);PE(16:0/18:0);PI(20:4/16:0);PE(16:1/16:0);PE(16:0/22:4);PE(22:2/12:0);PE(20:4/22:2);PE(16:0/20:5);PE(20:1/20:4) |
| Autophagy - other | 0.326675901 | PE(16:0/20:3);PE(18:0/20:5);PE(18:1/16:1);PE(20:4/14:0);PI(18:1/20:4);PI(18:0/20:5);PE(18:2/16:0);PE(18:0/14:0);PI(20:3/18:0);PE(16:0/22:4);PE(16:0/18:0);PE(16:1/16:0);PI(20:4/16:0);PE(20:4/22:2);PE(16:0/20:5);PE(22:2/12:0);PE(20:1/20:4) |
| Autophagy - animal | 0.326675901 | PE(20:4/22:2);PE(16:0/20:5);PE(22:2/12:0);PE(20:1/20:4);PE(18:0/14:0);PI(20:3/18:0);PE(16:0/22:4);PE(16:0/18:0);PE(16:1/16:0);PI(20:4/16:0);PI(18:0/20:5);PE(18:2/16:0);PE(16:0/20:3);PE(18:1/16:1);PE(18:0/20:5);PE(20:4/14:0);PI(18:1/20:4) |
| Phosphatidylinositol signaling system | 0.383219083 | PI(20:4/16:0);PI(20:3/18:0);DG(20:0/18:2/0:0);DG(16:1/20:0/0:0);DG(14:0/18:2/0:0);DG(16:0/18:0/0:0);PI(18:1/20:4);DG(18:4/18:1/0:0);DG(18:2/20:3/0:0);DG(18:0/18:2/0:0);PI(18:0/20:5);DG(18:0/18:0/0:0);DG(16:0/20:1/0:0) |
| Arachidonic acid metabolism | 0.411622438 | PC(16:0/20:5);PC(16:0/20:3);PC(18:0/22:0);PC(20:5/12:0);PC(20:1/20:4);PC(18:1/20:5);PC(20:1/18:2);PC(14:0/18:2);PC(18:1/18:4);PC(20:3/18:1);PC(18:0/20:3);15-OXOETE;PC(22:4/18:1);PC(18:2/20:3) |
| Neurotrophin signaling pathway | 0.482126318 | CER(D18:1/22:1);CER(D18:1/26:1);CER(D18:1/22:0) |
| AGE-RAGE signaling pathway in diabetic complications | 0.482126318 | CER(D18:1/26:1);CER(D18:1/22:1);CER(D18:1/22:0) |
| alpha-Linolenic acid metabolism | 0.49693947 | PC(18:2/20:3);PC(22:4/18:1);PC(20:3/18:1);PC(18:0/20:3);PC(14:0/18:2);PC(18:1/18:4);PC(20:5/12:0);PC(20:1/18:2);PC(18:1/20:5);PC(20:1/20:4);PC(16:0/20:3);PC(16:0/20:5);PC(18:0/22:0) |
| Linoleic acid metabolism | 0.519430014 | PC(20:1/18:2);PC(20:1/20:4);PC(18:1/20:5);PC(20:5/12:0);PC(18:1/18:4);PC(14:0/18:2);PC(18:0/22:0);PC(16:0/20:5);PC(16:0/20:3);PC(22:4/18:1);PC(18:2/20:3);PC(18:0/20:3);PC(20:3/18:1) |
| Long-term depression | 0.635857434 | DG(20:0/18:2/0:0);DG(18:0/18:2/0:0);DG(16:0/20:1/0:0);DG(18:0/18:0/0:0);DG(16:0/18:0/0:0);DG(14:0/18:2/0:0);DG(16:1/20:0/0:0);DG(18:4/18:1/0:0);DG(18:2/20:3/0:0) |
| Retrograde endocannabinoid signaling | 0.641198507 | PC(18:0/22:0);PE(16:0/18:0);PE(16:1/16:0);PE(18:0/14:0);PC(18:1/20:5);PC(20:1/20:4);PE(16:0/20:5);PC(18:0/20:3);PC(20:3/18:1);PE(18:1/16:1);PC(22:4/18:1);PE(18:2/16:0);PC(16:0/20:5);PC(16:0/20:3);PE(16:0/22:4);PC(20:1/18:2);PE(20:1/20:4);PC(20:5/12:0);PC(14:0/18:2);PE(22:2/12:0);PC(18:1/18:4);PE(20:4/22:2);PE(20:4/14:0);PE(18:0/20:5);PE(16:0/20:3);PC(18:2/20:3) |
| Sphingolipid metabolism | 0.703076674 | CER(D18:1/22:0);SM(D18:0/16:0);CER(D18:1/26:1);SM(D18:0/18:1);CER(D18:1/22:1);SM(D18:2/24:1) |
| Sphingolipid signaling pathway | 0.703076674 | SM(D18:2/24:1);SM(D18:0/16:0);CER(D18:1/22:0);CER(D18:1/22:1);SM(D18:0/18:1);CER(D18:1/26:1) |
| Kaposi sarcoma-associated herpesvirus infection | 0.704349197 | PE(18:0/14:0);PE(16:0/20:3);PE(18:0/20:5);PE(18:1/16:1);PE(16:0/22:4);PE(16:0/18:0);PE(20:4/14:0);PE(16:1/16:0);PE(20:4/22:2);PE(16:0/20:5);PE(22:2/12:0);PE(20:1/20:4);PE(18:2/16:0) |
| Pathogenic Escherichia coli infection | 0.722152934 | PE(16:0/22:4);PE(16:1/16:0);PE(20:4/14:0);PE(16:0/18:0);PE(16:0/20:3);PE(18:0/14:0);PE(18:1/16:1);PE(18:0/20:5);PE(18:2/16:0);PE(20:1/20:4);PE(16:0/20:5);PE(20:4/22:2);PE(22:2/12:0) |
| Necroptosis | 0.727158628 | SM(D18:2/24:1);CER(D18:1/22:0);SM(D18:0/16:0);SM(D18:0/18:1);CER(D18:1/26:1);CER(D18:1/22:1) |
| Insulin resistance | 0.992869557 | FFA(4:0);FFA(8:0);TG(14:0/18:1/20:3);TG(14:0/20:5/22:4);FFA(24:5);TG(14:0/22:0/22:2);TG(16:1/16:1/20:3);FFA(12:0);TG(16:0/18:0/22:0);TG(14:0/20:3/22:6);FFA(22:2);FFA(22:4);TG(14:0/18:2/22:6);TG(18:2/18:2/20:0);TG(18:1/20:0/20:0);FFA(24:6);TG(14:0/16:0/22:0);FFA(22:3);FFA(22:1);TG(18:1/18:1/18:2);FFA(17:1);CER(D18:1/22:1);FFA(20:2);TG(14:0/20:0/22:0);FFA(16:1);FFA(24:4);CER(D18:1/26:1);CER(D18:1/22:0);TG(14:0/20:1/20:3);TG(16:0/20:0/22:0) |
| Cholesterol metabolism | 0.99522447 | FFA(22:1);TG(18:1/18:1/18:2);FFA(17:1);FFA(20:2);TG(14:0/20:0/22:0);FFA(16:1);FFA(24:4);TG(14:0/20:1/20:3);TG(16:0/20:0/22:0);TG(14:0/18:1/20:3);FFA(4:0);FFA(8:0);FFA(24:5);TG(14:0/20:5/22:4);TG(14:0/22:0/22:2);TG(16:1/16:1/20:3);FFA(12:0);TG(16:0/18:0/22:0);TG(14:0/20:3/22:6);FFA(22:4);FFA(22:2);TG(14:0/18:2/22:6);TG(18:2/18:2/20:0);TG(18:1/20:0/20:0);FFA(24:6);TG(14:0/16:0/22:0);FFA(22:3) |
| Thermogenesis | 0.996189845 | TG(14:0/20:3/22:6);TG(14:0/18:2/22:6);FFA(22:4);FFA(22:2);TG(14:0/16:0/22:0);FFA(22:3);FFA(24:6);TG(18:1/20:0/20:0);TG(18:2/18:2/20:0);TG(14:0/18:1/20:3);FFA(4:0);FFA(8:0);TG(14:0/22:0/22:2);FFA(24:5);TG(14:0/20:5/22:4);TG(16:0/18:0/22:0);TG(16:1/16:1/20:3);FFA(12:0);TG(16:0/20:0/22:0);TG(14:0/20:1/20:3);TG(18:1/18:1/18:2);FFA(22:1);FFA(20:2);FFA(17:1);FFA(16:1);TG(14:0/20:0/22:0);FFA(24:4) |
| Regulation of lipolysis in adipocytes | 0.996603165 | TG(14:0/16:0/22:0);FFA(22:3);FFA(24:6);TG(18:1/20:0/20:0);TG(18:2/18:2/20:0);TG(14:0/20:3/22:6);TG(14:0/18:2/22:6);FFA(22:2);FFA(22:4);TG(14:0/22:0/22:2);TG(14:0/20:5/22:4);FFA(24:5);TG(16:0/18:0/22:0);TG(16:1/16:1/20:3);FFA(12:0);FFA(4:0);FFA(8:0);TG(14:0/18:1/20:3);TG(16:0/20:0/22:0);TG(14:0/20:1/20:3);FFA(16:1);TG(14:0/20:0/22:0);FFA(24:4);TG(18:1/18:1/18:2);FFA(22:1);FFA(20:2);FFA(17:1) |
| Vitamin digestion and absorption | 0.996603165 | TG(16:0/20:0/22:0);TG(14:0/20:1/20:3);TG(18:1/18:1/18:2);FFA(22:1);FFA(20:2);FFA(17:1);FFA(16:1);TG(14:0/20:0/22:0);FFA(24:4);TG(14:0/20:3/22:6);TG(14:0/18:2/22:6);FFA(22:4);FFA(22:2);FFA(24:6);TG(14:0/16:0/22:0);FFA(22:3);TG(18:2/18:2/20:0);TG(18:1/20:0/20:0);TG(14:0/18:1/20:3);FFA(4:0);FFA(8:0);TG(14:0/22:0/22:2);FFA(24:5);TG(14:0/20:5/22:4);TG(16:0/18:0/22:0);TG(16:1/16:1/20:3);FFA(12:0) |
| Fat digestion and absorption | 0.998859234 | TG(18:1/20:0/20:0);TG(18:2/18:2/20:0);FFA(24:6);TG(14:0/16:0/22:0);FFA(22:3);TG(14:0/20:3/22:6);FFA(22:4);FFA(22:2);TG(14:0/18:2/22:6);FFA(24:5);TG(14:0/20:5/22:4);TG(14:0/22:0/22:2);FFA(12:0);TG(16:1/16:1/20:3);TG(16:0/18:0/22:0);TG(14:0/18:1/20:3);FFA(4:0);FFA(8:0);TG(14:0/20:1/20:3);TG(16:0/20:0/22:0);TG(14:0/20:0/22:0);FFA(16:1);FFA(24:4);FFA(22:1);TG(18:1/18:1/18:2);FFA(17:1);FFA(20:2) |
| Glycerolipid metabolism | 0.998869293 | TG(14:0/20:1/20:3);TG(16:0/20:0/22:0);DG(18:0/18:2/0:0);TG(18:1/18:1/18:2);FFA(22:1);FFA(17:1);DG(18:0/18:0/0:0);FFA(20:2);TG(14:0/20:0/22:0);FFA(16:1);FFA(24:4);DG(20:0/18:2/0:0);TG(14:0/20:3/22:6);FFA(22:4);DG(16:0/18:0/0:0);FFA(22:2);DG(14:0/18:2/0:0);TG(14:0/18:2/22:6);DG(16:1/20:0/0:0);TG(18:2/18:2/20:0);TG(18:1/20:0/20:0);TG(14:0/16:0/22:0);FFA(22:3);FFA(24:6);TG(14:0/18:1/20:3);FFA(8:0);FFA(4:0);DG(16:0/20:1/0:0);FFA(24:5);DG(18:2/20:3/0:0);DG(18:4/18:1/0:0);TG(14:0/20:5/22:4);TG(14:0/22:0/22:2);TG(16:1/16:1/20:3);FFA(12:0);TG(16:0/18:0/22:0) |
| Metabolic pathways | 0.999999867 | SM(D18:0/16:0);PE(18:0/14:0);PE(16:1/16:0);TG(18:1/20:0/20:0);DG(16:1/20:0/0:0);PE(16:0/20:5);PC(20:1/20:4);DG(20:0/18:2/0:0);TG(16:0/18:0/22:0);TG(14:0/22:0/22:2);DG(18:4/18:1/0:0);TG(14:0/20:5/22:4);PG(18:1/16:1);PI(18:0/20:5);DG(16:0/20:1/0:0);TG(14:0/18:1/20:3);PC(16:0/20:5);PE(16:0/22:4);PC(16:0/20:3);PE(22:2/12:0);PC(18:1/18:4);PC(14:0/18:2);PC(20:1/18:2);PE(20:1/20:4);PC(20:5/12:0);CER(D18:1/22:0);PE(20:4/14:0);TG(14:0/20:0/22:0);DG(18:0/18:0/0:0);DG(18:0/18:2/0:0);PI(20:3/18:0);PG(18:0/18:1);TG(14:0/16:0/22:0);PE(16:0/18:0);PC(18:0/22:0);TG(18:2/18:2/20:0);DG(14:0/18:2/0:0);TG(14:0/18:2/22:6);DG(16:0/18:0/0:0);TG(14:0/20:3/22:6);PC(18:1/20:5);PE(18:1/16:1);TG(16:1/16:1/20:3);PI(18:1/20:4);PC(18:0/20:3);DG(18:2/20:3/0:0);PC(20:3/18:1);PS(20:4/20:0);PC(22:4/18:1);PE(18:2/16:0);PI(20:4/16:0);PG(18:0/16:0);PE(20:4/22:2);TG(16:0/20:0/22:0);TG(14:0/20:1/20:3);PE(18:0/20:5);CER(D18:1/26:1);COENZYMEQ10;PE(16:0/20:3);SM(D18:2/24:1);CER(D18:1/22:1);SM(D18:0/18:1);PC(18:2/20:3);TG(18:1/18:1/18:2);PS(18:0/20:4) |
