# Supplementary material for: Lipidomics profiling of goose granulosa cell model of stearoyl-CoA desaturase function identifies a pattern of lipid droplets associated with follicle development
Source: Cell Biosci. 2021 May 22;11:95. doi: 10.1186/s13578-021-00604-6 (PMC8141238; doi:10.1186/s13578-021-00604-6)
Supplement: Supplementary file 8 — Additional file 8: Table S3. Basic characteristics of tags in each sample libraries and data of sequencing reads mapping to the reference genome. [file 13578_2021_604_MOESM8_ESM.docx]

| **Supplementary Table 3. Basic characteristics of tags in each sample libraries and data of sequencing reads mapping to the reference genom** | | | | | | | | | | | | | | | | | |  |  |
| --- | --- | --- | --- | --- | --- | --- | --- | --- | --- | --- | --- | --- | --- | --- | --- | --- | --- | --- | --- |
| Sample | Raw reads | Clean reads  (percentage^a^) | Clean Base(G) | Containing N (percentage) | Low quantity (percentage) | Adapter related (percentage) | Error Rate(%) | Q20(%) | Q30(%) | GC Content(%) | Reads mapped (percentage^b^) | Uniquely mapped | Multiple mapped | Read1 mapped | Read2 mapped | +' mapped | '-' mapped | |  |
| NC1 | 53722460 | 51361068(95.6%) | 7.7 | 6406(0.01%) | 296690(0.55%) | 2058296(3.83%) | 0.03 | 97.25 | 92.52 | 51.33 | 38221726(74.42%) | 37299257(72.62%) | 1300583(1.80%) | 18732723(36.47%) | 18566534(36.15%) | 18497023(36.01%) | 18802234(36.61%) | | |
| NC2 | 57336348 | 54920308(95.79%) | 8.24 | 6730(0.01%) | 358798(0.63%) | 2050512(3.58%) | 0.03 | 97.21 | 92.5 | 51.66 | 40334966(73.44%) | 39333650(71.62%) | 1427134(1.82%) | 19760261(35.98%) | 19573389(35.64%) | 19461789(35.44%) | 19871861(36.18%) | | |
| NC3 | 49956772 | 48416686(96.92%) | 7.26 | 532(0%) | 197972(0.4%) | 1341582(2.69%) | 0.03 | 97.58 | 93.11 | 50.23 | 37211780(76.86%) | 36429529(75.24%) | 1050226(1.62%) | 18285027(37.77%) | 18144502(37.48%) | 18112949(37.41%) | 18316580(37.83%) | | |
| OG1 | 57064320 | 54930402(96.26) | 8.24 | 6106(0.01%) | 335914(0.59%) | 1791898(3.14%) | 0.03 | 97.36 | 92.79 | 51.89 | 39799710(72.45%) | 38888534(70.80%) | 1249918(1.66%) | 19524898(35.54%) | 19363636(35.25%) | 19253573(35.05%) | 19634961(35.75%) | | |
| OG2 | 59376402 | 57123224(96.21%) | 8.57 | 6784(0.01%) | 346318(0.58%) | 1900076(3.2%) | 0.03 | 97.31 | 92.7 | 51.64 | 41869531(73.30%) | 40918261(71.63%) | 1342789(1.67%) | 20545830(35.97%) | 20372431(35.66%) | 20253603(35.46%) | 20664658(36.18%) | | |
| OG3 | 50664522 | 49271218(97.25%) | 7.39 | 524(0%) | 214532(0.42%) | 1178248(2.33%) | 0.03 | 97.4 | 92.79 | 50.85 | 36983049(75.06%) | 36187615(73.45%) | 1052113(1.61%) | 18180264(36.90%) | 18007351(36.55%) | 18001787(36.54%) | 18185828(36.91%) | | |
| OS1 | 48298886 | 46565782(96.41%) | 6.98 | 5842(0.01%) | 314606(0.65%) | 1412656(2.92%) | 0.03 | 97.14 | 92.34 | 52.73 | 33184462(71.26%) | 32421281(69.62%) | 1062503(1.64%) | 16292628(34.99%) | 16128653(34.64%) | 15992741(34.34%) | 16428540(35.28%) | | |
| OS2 | 60975480 | 58371054(95.73%) | 8.76 | 7452(0.01%) | 351990(0.58%) | 2244984(3.68%) | 0.03 | 97.25 | 92.56 | 51.74 | 42806504(73.34%) | 41806750(71.62%) | 1381343(1.71%) | 21006107(35.99%) | 20800643(35.64%) | 20705871(35.47%) | 21100879(36.15%) | | |
| OS3 | 64116744 | 62169854(96.96%) | 9.33 | 734(0%) | 306742(0.48%) | 1639414(2.56%) | 0.03 | 97.35 | 92.7 | 50.81 | 46947020(75.51%) | 45903639(73.84%) | 1396953(1.68%) | 23075470(37.12%) | 22828169(36.72%) | 22821171(36.71%) | 23082468(37.13%) | | |
| SC1 | 55108380 | 53139140(96.43%) | 7.97 | 662(0%) | 233170(0.42%) | 1735408(3.15%) | 0.03 | 97.43 | 92.81 | 50.6 | 40391480(76.01%) | 39464893(74.27%) | 1250187(1.74%) | 19824740(37.31%) | 19640153(36.96%) | 19645420(36.97%) | 19819473(37.30%) | | |
| SC2 | 72179346 | 69838540(96.76%) | 10.48 | 8120(0.01%) | 358094(0.5%) | 1974592(2.74%) | 0.03 | 97.34 | 92.65 | 51.16 | 52204820(74.75%) | 51026204(73.06%) | 1608902(1.69%) | 25646687(36.72%) | 25379517(36.34%) | 25361777(36.31%) | 25664427(36.75%) | | |
| SC3 | 51832194 | 50106618(96.67%) | 7.52 | 574(0%) | 216096(0.42%) | 1508906(2.91%) | 0.03 | 97.38 | 92.77 | 50.57 | 38128755(76.10%) | 37300623(74.44%) | 1088345(1.65%) | 18737645(37.40%) | 18562978(37.05%) | 18565772(37.05%) | 18734851(37.39%) | | |
| SF1 | 61116384 | 58842928(96.28%) | 8.83 | 678(0%) | 284516(0.47%) | 1988262(3.25%) | 0.03 | 97.28 | 92.55 | 50.76 | 44391561(75.44%) | 43403516(73.76%) | 1369380(1.68%) | 21817322(37.08%) | 21586194(36.68%) | 21592381(36.69%) | 21811135(37.07%) | | |
| SF2 | 57345946 | 55396330(96.6%) | 8.31 | 7005(0.01%) | 265992(0.46%) | 1676620(2.92%) | 0.03 | 97.41 | 92.78 | 51.31 | 41380637(74.70%) | 40457119(73.03%) | 1275003(1.67%) | 20319990(36.68%) | 20137129(36.35%) | 20124793(36.33%) | 20332326(36.70%) | | |
| SF3 | 56377786 | 54323066(96.36%) | 8.15 | 612(0%) | 246268(0.44%) | 1807840(3.21%) | 0.03 | 97.49 | 92.98 | 50.88 | 40979422(75.44%) | 40025579(73.68%) | 1267272(1.76%) | 20087257(36.98%) | 19938322(36.70%) | 19919862(36.67%) | 20105717(37.01%) | | |
| ST1 | 53548076 | 51441348(96.07%) | 7.72 | 598(0%) | 252346(0.47%) | 1853784(3.46%) | 0.03 | 97.5 | 93.05 | 51.21 | 38698041(75.23%) | 37917254(73.71%) | 1080129(1.52%) | 19031039(37.00%) | 18886215(36.71%) | 18851636(36.65%) | 19065618(37.06%) | | |
| ST2 | 56895126 | 54851824(96.41%) | 8.23 | 6860(0.01%) | 287088(0.5%) | 1749354(3.07%) | 0.03 | 97.53 | 93.06 | 52.04 | 40411626(73.67%) | 39562382(72.13%) | 1158295(1.55%) | 19864634(36.22%) | 19697748(35.91%) | 19632004(35.79%) | 19930378(36.33%) | | |
| ST3 | 50073444 | 48640872(97.14%) | 7.3 | 470(0%) | 200436(0.4%) | 1231666(2.46%) | 0.03 | 97.34 | 92.6 | 50.71 | 37021485(76.11%) | 36173328(74.37%) | 1108875(1.74%) | 18167919(37.35%) | 18005409(37.02%) | 18008256(37.02%) | 18165072(37.35%) | | |
| Note: a The number of clean reading frames of total raw reading frames. b The number of all mapped reads out of total clean reads. Q20 represents an error rate of less than 0.01, and Q30 represents an error rate of less than 0.001 ‘‘+’’ refers to sense strands, ‘‘-’’ refers to anti-sense strands. ‘‘Non-splice reads’’ refers to reads for the entire sequence is mapped to one exon; ‘‘Splice reads’’ also called junction reads, refers to reads mapped to the border of exon. | | | | | | | | | | | | | | | | | |  |  |
