# Supplementary material for: Lipidomics profiling of goose granulosa cell model of stearoyl-CoA desaturase function identifies a pattern of lipid droplets associated with follicle development
Source: Cell Biosci. 2021 May 22;11:95. doi: 10.1186/s13578-021-00604-6 (PMC8141238; doi:10.1186/s13578-021-00604-6)
Supplement: Supplementary file 9 — Additional file 9: Table S4. The detail of DEGs were assigned with lipid-related GO slim terms. [file 13578_2021_604_MOESM9_ESM.docx]

| **Supplementary Table 4. The detail of DGEs were assigned with lipid-related GO slim terms** | |
| --- | --- |
| **Comparison I: NC vs. OS comparison** | |
| Description | geneID |
| lipid transporter activity | gene-LOC106041217 |
| lipid binding | gene-BAIAP2L1/gene-F3/gene-PROM1 |
| phospholipid binding | gene-F3 |
| regulation of lipid kinase activity | gene-PIK3IP1 |
| regulation of phospholipid metabolic process | gene-PIK3IP1 |
| sphingolipid metabolic process | gene-ACER2 |
| regulation of lipid metabolic process | gene-PIK3IP1 |
| cellular lipid metabolic process | gene-ACER2/gene-PIK3IP1/gene-PLA2G15/gene-RDH10 |
| phospholipid metabolic process | gene-PIK3IP1/gene-PLA2G15 |
| membrane lipid metabolic process | gene-ACER2 |
| lipid biosynthetic process | gene-LOC106044184/gene-RDH10 |
| lipid modification | gene-PLA2G15 |
| glycerophospholipid metabolic process | gene-PLA2G15 |
| glycerolipid metabolic process | gene-PLA2G15 |
| steroid hydroxylase activity | gene-LOC106044184 |
| steroid binding | gene-PROM1 |
| steroid metabolic process | gene-LOC106044184/gene-PLA2G15 |
| steroid biosynthetic process | gene-LOC106044184 |
| cholesterol binding | gene-PROM1 |
| cholesterol metabolic process | gene-LOC106044184/gene-PLA2G15 |
| **Comparison II: OG vs. OS comparison** | |
| lipid binding | gene-CADPS2/gene-LOC106031807/gene-LOC106049214 |
| neutral lipid biosynthetic process | gene-MOGAT1 |
| regulation of lipid transport | gene-GNRH1 |
| regulation of lipid localization | gene-GNRH1 |
| neutral lipid metabolic process | gene-MOGAT1 |
| glycerolipid metabolic process | gene-MOGAT1/gene-SERINC4 |
| inositol lipid-mediated signaling | gene-FRMPD3 |
| glycerolipid biosynthetic process | gene-MOGAT1 |
| sphingolipid metabolic process | gene-SERINC4 |
| phospholipid biosynthetic process | gene-SERINC4 |
| lipid biosynthetic process | gene-MOGAT1/gene-SERINC4 |
| membrane lipid metabolic process | gene-SERINC4 |
| lipid transport | gene-GNRH1 |
| lipid localization | gene-GNRH1 |
| glycerophospholipid metabolic process | gene-SERINC4 |
| response to lipid | gene-GNRH1 |
| phospholipid metabolic process | gene-SERINC4 |
| cellular lipid metabolic process | gene-MOGAT1/gene-SERINC4 |
| steroid binding | gene-LOC106031807 |
| response to steroid hormone | gene-GNRH1 |
| fatty acid derivative binding | gene-LOC106049214 |
| **Comparison III: SC vs. ST comparison** | |
| lipid droplet | gene-LOC106043072 |
| lipid transporter activity | gene-ABCG1/gene-ATP10D/gene-PRELID1/gene-STAR |
| phospholipid transporter activity | gene-ATP10D/gene-PRELID1 |
| phospholipid-translocating ATPase activity | gene-ATP10D |
| lipid binding | gene-F3/gene-NSF/gene-PTEN/gene-SNX13/gene-STAR/gene-TEX2 |
| phospholipid binding | gene-F3/gene-SNX13 |
| lipid transport | gene-ABCD2/gene-ABCG1/gene-ATP10D/gene-LOC106033779/gene-NOS2/gene-PRELID1/gene-STAR/gene-TEX2 |
| lipid localization | gene-ABCD2/gene-ABCG1/gene-ATP10D/gene-LOC106033779/gene-NOS2/gene-PRELID1/gene-STAR/gene-TEX2 |
| lipid biosynthetic process | gene-FDPS/gene-INSIG1/gene-LOC106033779/gene-LOC106043072/gene-LOC106045741/gene-NSDHL/gene-SERINC5/gene-ST8SIA1/gene-ST8SIA4/gene-STAR |
| response to lipid | gene-HSPA2/gene-INSIG1/gene-LOC106033779/gene-NOS2/gene-OTUD3/gene-PTGFR/gene-TNIP3 |
| intracellular lipid transport | gene-ABCG1/gene-STAR |
| glycosphingolipid metabolic process | gene-ST8SIA1/gene-ST8SIA4 |
| regulation of lipid biosynthetic process | gene-INSIG1/gene-STAR |
| cellular response to lipid | gene-INSIG1/gene-LOC106033779/gene-PTGFR/gene-TNIP3 |
| cellular lipid metabolic process | gene-ABCD2/gene-CROT/gene-FDPS/gene-INSIG1/gene-LOC106043072/gene-PPAP2B/gene-PTEN/gene-SERINC5/gene-ST8SIA1/gene-ST8SIA4/gene-STAR |
| sphingolipid metabolic process | gene-SERINC5/gene-ST8SIA1/gene-ST8SIA4 |
| phospholipid transport | gene-ATP10D/gene-PRELID1 |
| protein transport within lipid bilayer | gene-LOC106038594/gene-RILPL1 |
| sphingolipid biosynthetic process | gene-ST8SIA1/gene-ST8SIA4 |
| phospholipid dephosphorylation | gene-PPAP2B/gene-PTEN |
| glycolipid biosynthetic process | gene-ST8SIA1/gene-ST8SIA4 |
| phospholipid biosynthetic process | gene-FDPS/gene-LOC106043072/gene-SERINC5 |
| membrane lipid metabolic process | gene-SERINC5/gene-ST8SIA1/gene-ST8SIA4 |
| glycolipid metabolic process | gene-ST8SIA1/gene-ST8SIA4 |
| phospholipid metabolic process | gene-FDPS/gene-LOC106043072/gene-PPAP2B/gene-PTEN/gene-SERINC5 |
| lipid modification | gene-ABCD2/gene-PPAP2B/gene-PTEN |
| membrane lipid biosynthetic process | gene-ST8SIA1/gene-ST8SIA4 |
| regulation of lipid metabolic process | gene-INSIG1/gene-STAR |
| positive regulation of lipid biosynthetic process | gene-STAR |
| regulation of lipid transport | gene-LOC106033779 |
| phospholipid translocation | gene-ATP10D |
| lipid translocation | gene-ATP10D |
| regulation of lipid localization | gene-LOC106033779 |
| regulation of membrane lipid distribution | gene-ATP10D |
| lipid oxidation | gene-ABCD2 |
| lipid homeostasis | gene-INSIG1 |
| neutral lipid metabolic process | gene-INSIG1 |
| positive regulation of lipid metabolic process | gene-STAR |
| glycerolipid metabolic process | gene-INSIG1/gene-PTEN/gene-SERINC5 |
| glycerophospholipid metabolic process | gene-PTEN/gene-SERINC5 |
| cellular lipid catabolic process | gene-ABCD2 |
| lipid catabolic process | gene-ABCD2 |
| steroid dehydrogenase activity, acting on the CH-OH group of donors, NAD or NADP as acceptor | gene-LOC106045741/gene-NSDHL |
| steroid dehydrogenase activity | gene-LOC106045741/gene-NSDHL |
| steroid hormone receptor binding | gene-HSPA2 |
| steroid binding | gene-STAR |
| steroid biosynthetic process | gene-FDPS/gene-INSIG1/gene-LOC106033779/gene-LOC106045741/gene-NSDHL/gene-STAR |
| steroid metabolic process | gene-FDPS/gene-INSIG1/gene-LOC106033779/gene-LOC106045741/gene-NSDHL/gene-STAR |
| regulation of steroid metabolic process | gene-INSIG1/gene-STAR |
| response to steroid hormone | gene-HSPA2 |
| response to fatty acid | gene-PTGFR |
| very long-chain fatty acid metabolic process | gene-ABCD2 |
| fatty acid derivative transport | gene-NOS2 |
| fatty acid metabolic process | gene-ABCD2/gene-CROT/gene-INSIG1 |
| fatty acid transport | gene-NOS2 |
| fatty acid beta-oxidation | gene-ABCD2 |
| fatty acid oxidation | gene-ABCD2 |
| fatty acid catabolic process | gene-ABCD2 |
| fatty acid biosynthetic process | gene-INSIG1 |
| cholesterol binding | gene-STAR |
| cholesterol transport | gene-ABCD2/gene-ABCG1/gene-STAR |
| cholesterol metabolic process | gene-FDPS/gene-INSIG1/gene-STAR |
| cholesterol homeostasis | gene-INSIG1 |
| **Comparison IV: SC vs. SF comparison** | |
| lipid binding | gene-AMER1/gene-ARAP1/gene-DLC1/gene-F3/gene-KCNQ1/gene-NSF/gene-PCTP/gene-PLA2G4A/gene-PPARD/gene-PTEN/gene-SNX6/gene-TEX2/gene-WIPI2 |
| phospholipid transporter activity | gene-ATP11B/gene-PRELID1 |
| phospholipid-translocating ATPase activity | gene-ATP11B |
| phospholipid binding | gene-AMER1/gene-F3/gene-KCNQ1/gene-PLA2G4A/gene-SNX6/gene-WIPI2 |
| lipid transporter activity | gene-ATP11B/gene-PRELID1 |
| calcium-dependent phospholipid binding | gene-PLA2G4A |
| glycerolipid catabolic process | gene-ABHD5/gene-PLA2G4A/gene-PNPLA8 |
| lipid biosynthetic process | gene-ABHD5/gene-FDX1/gene-LOC106033238/gene-LOC106045741/gene-PNPLA8/gene-PTGS2/gene-SCD/gene-SDR16C5/gene-SERINC5/gene-SGMS1 |
| cellular lipid metabolic process | gene-ABHD5/gene-ALG8/gene-CROT/gene-LOC106033238/gene-LOC106038668/gene-MTMR8/gene-PIK3R1/gene-PLA2G4A/gene-PNPLA8/gene-PPAP2B/gene-PTEN/gene-PTGS2/gene-SCD/gene-SDR16C5/gene-SERINC5/gene-SGMS1 |
| glycerophospholipid catabolic process | gene-PLA2G4A/gene-PNPLA8 |
| glycerophospholipid catabolic process | gene-PLA2G4A/gene-PNPLA8 |
| phospholipid dephosphorylation | gene-MTMR8/gene-PPAP2B/gene-PTEN |
| glycerophospholipid metabolic process | gene-ABHD5/gene-MTMR8/gene-PIK3R1/gene-PLA2G4A/gene-PNPLA8/gene-PTEN/gene-SERINC5 |
| phospholipid catabolic process | gene-PLA2G4A/gene-PNPLA8 |
| lipid modification | gene-LOC106038668/gene-MTMR8/gene-PIK3R1/gene-PPAP2B/gene-PTEN |
| glycerolipid metabolic process | gene-ABHD5/gene-MTMR8/gene-PIK3R1/gene-PLA2G4A/gene-PNPLA8/gene-PTEN/gene-SERINC5 |
| phospholipid transport | gene-ATP11B/gene-PRELID1 |
| lipid localization | gene-ABHD5/gene-ATP11B/gene-PNPLA8/gene-PRELID1/gene-TEX2 |
| lipid catabolic process | gene-ABHD5/gene-LOC106029986/gene-PLA2G4A/gene-PNPLA8/gene-RAB7A |
| positive regulation of lipid metabolic process | gene-ABHD5/gene-PTGS2 |
| cellular lipid catabolic process | gene-ABHD5/gene-PLA2G4A/gene-PNPLA8 |
| phospholipid biosynthetic process | gene-ABHD5/gene-SERINC5/gene-SGMS1 |
| lipid transport | gene-ATP11B/gene-PNPLA8/gene-PRELID1/gene-TEX2 |
| protein lipidation | gene-WIPI2/gene-ZDHHC15 |
| positive regulation of lipid biosynthetic process | gene-PTGS2 |
| phospholipid translocation | gene-ATP11B |
| lipid translocation | gene-ATP11B |
| sphingolipid metabolic process | gene-SERINC5/gene-SGMS1 |
| regulation of lipid biosynthetic process | gene-PTGS2 |
| regulation of lipid metabolic process | gene-ABHD5/gene-PTGS2 |
| regulation of lipid localization | gene-ABHD5 |
| response to lipid | gene-HSPA2/gene-PTGFR/gene-SLC2A1/gene-ZFP36L1 |
| regulation of membrane lipid distribution | gene-ATP11B |
| lipid phosphorylation | gene-PIK3R1 |
| sphingolipid biosynthetic process | gene-SGMS1 |
| neutral lipid metabolic process | gene-ABHD5 |
| membrane lipid metabolic process | gene-SERINC5/gene-SGMS1 |
| cellular response to lipid | gene-PTGFR/gene-ZFP36L1 |
| glycerophospholipid biosynthetic process | gene-ABHD5 |
| glycerolipid biosynthetic process | gene-ABHD5 |
| membrane lipid biosynthetic process | gene-SGMS1 |
| lipid droplet | gene-ABHD5/gene-SDR16C5 |
| steroid hormone receptor activity | gene-NR1D2/gene-NR2E1/gene-PPARD/gene-RORA |
| steroid hormone receptor binding | gene-DGKQ/gene-HSPA2 |
| steroid dehydrogenase activity, acting on the CH-OH group of donors, NAD or NADP as acceptor | gene-LOC106045741 |
| steroid dehydrogenase activity | gene-LOC106045741 |
| response to corticosteroid | gene-SLC2A1/gene-ZFP36L1 |
| response to steroid hormone | gene-HSPA2/gene-SLC2A1/gene-ZFP36L1 |
| steroid biosynthetic process | gene-FDX1/gene-LOC106045741 |
| cellular response to steroid hormone stimulus | gene-ZFP36L1 |
| steroid metabolic process | gene-FDX1/gene-LOC106045741 |
| unsaturated fatty acid metabolic process | gene-PNPLA8/gene-PTGS2/gene-SCD |
| fatty acid biosynthetic process | gene-LOC106033238/gene-PNPLA8/gene-PTGS2/gene-SCD |
| fatty acid metabolic process | gene-CROT/gene-LOC106033238/gene-LOC106038668/gene-PNPLA8/gene-PTGS2/gene-SCD |
| long-chain fatty acid metabolic process | gene-PNPLA8/gene-PTGS2 |
| fatty acid derivative biosynthetic process | gene-PNPLA8/gene-PTGS2 |
| fatty acid derivative metabolic process | gene-PNPLA8/gene-PTGS2 |
| response to fatty acid | gene-PTGFR |
| long-chain fatty acid transport | gene-PNPLA8 |
| fatty acid derivative transport | gene-PNPLA8 |
| fatty acid transport | gene-PNPLA8 |
| unsaturated fatty acid biosynthetic process | gene-PNPLA8/gene-PTGS2/gene-SCD |
| cholesterol metabolic process | gene-FDX1 |
