# Supplementary material for: Lipidomics profiling of goose granulosa cell model of stearoyl-CoA desaturase function identifies a pattern of lipid droplets associated with follicle development
Source: Cell Biosci. 2021 May 22;11:95. doi: 10.1186/s13578-021-00604-6 (PMC8141238; doi:10.1186/s13578-021-00604-6)
Supplement: Supplementary file 10 — Additional file 10: Table S5. The detail of lipids from glycerolipid/glycerophospholipid metabolism were subjected to a CCA together with transcript data of all those pathways as derived from SCD knockdown in GCs. [file 13578_2021_604_MOESM10_ESM.docx]

| **Supplementary Table 5. The detail of lipids from glycerolipid/glycerophospholipid metabolism were subjected to a CCA together with transcript data of all those pathways as derived from SCD knockdown in GCs** | |
| --- | --- |
| **glycerolipid metabolism** | |
| Index | Formula |
| LIPID-N-0072 | FFA(4:0) |
| LIPID-N-0075 | FFA(8:0) |
| LIPID-N-0078 | FFA(12:0) |
| LIPID-N-0091 | FFA(16:1) |
| LIPID-N-0092 | FFA(17:1) |
| LIPID-N-0096 | FFA(22:1) |
| LIPID-N-0100 | FFA(20:2) |
| LIPID-N-0101 | FFA(22:2) |
| LIPID-N-0105 | FFA(22:3) |
| LIPID-N-0108 | FFA(22:4) |
| LIPID-N-0109 | FFA(24:4) |
| LIPID-N-0112 | FFA(24:5) |
| LIPID-N-0114 | FFA(24:6) |
| LIPID-P-0126 | DG(16:0/18:0/0:0) |
| LIPID-P-0129 | DG(18:0/18:0/0:0) |
| LIPID-P-0144 | DG(16:0/20:1/0:0) |
| LIPID-P-0145 | DG(16:1/20:0/0:0) |
| LIPID-P-0152 | DG(14:0/18:2/0:0) |
| LIPID-P-0159 | DG(18:0/18:2/0:0) |
| LIPID-P-0163 | DG(20:0/18:2/0:0) |
| LIPID-P-0202 | DG(18:4/18:1/0:0) |
| LIPID-P-0205 | DG(18:2/20:3/0:0) |
| LIPID-P-0759 | TG(14:0/16:0/22:0) |
| LIPID-P-0767 | TG(14:0/20:0/22:0) |
| LIPID-P-0770 | TG(16:0/18:0/22:0) |
| LIPID-P-0772 | TG(16:0/20:0/22:0) |
| LIPID-P-0824 | TG(18:1/20:0/20:0) |
| LIPID-P-0881 | TG(14:0/22:0/22:2) |
| LIPID-P-0985 | TG(14:0/18:1/20:3) |
| LIPID-P-0994 | TG(18:1/18:1/18:2) |
| LIPID-P-0995 | TG(14:0/20:1/20:3) |
| LIPID-P-1006 | TG(18:2/18:2/20:0) |
| LIPID-P-1040 | TG(16:1/16:1/20:3) |
| LIPID-P-1158 | TG(14:0/18:2/22:6) |
| LIPID-P-1178 | TG(14:0/20:5/22:4) |
| LIPID-P-1182 | TG(14:0/20:3/22:6) |
| **glycerophospholipid metabolism** | |
| Index | Formula |
| LIPID-N-0119 | LPC(16:0/0:0) |
| LIPID-N-0129 | LPC(18:2/0:0) |
| LIPID-N-0135 | LPC(20:4/0:0) |
| LIPID-N-0142 | LPE(0:0/20:0) |
| LIPID-N-0211 | PC(14:0/18:2) |
| LIPID-N-0250 | PC(16:0/20:3) |
| LIPID-N-0258 | PC(18:0/20:3) |
| LIPID-N-0259 | PC(20:1/18:2) |
| LIPID-N-0286 | PC(20:3/18:1) |
| LIPID-N-0305 | PC(16:0/20:5) |
| LIPID-N-0307 | PC(18:1/18:4) |
| LIPID-N-0317 | PC(18:2/20:3) |
| LIPID-N-0322 | PC(20:1/20:4) |
| LIPID-N-0365 | PE(18:0/14:0) |
| LIPID-N-0366 | PE(16:0/18:0) |
| LIPID-N-0374 | PE(16:1/16:0) |
| LIPID-N-0405 | PE(18:1/16:1) |
| LIPID-N-0407 | PE(18:2/16:0) |
| LIPID-N-0443 | PE(16:0/20:3) |
| LIPID-N-0462 | PE(20:4/14:0) |
| LIPID-N-0476 | PE(16:0/22:4) |
| LIPID-N-0493 | PE(16:0/20:5) |
| LIPID-N-0499 | PE(18:0/20:5) |
| LIPID-N-0507 | PE(20:1/20:4) |
| LIPID-N-0535 | PE(20:4/22:2) |
| LIPID-N-0557 | PG(18:0/18:1) |
| LIPID-N-0564 | PG(18:1/16:1) |
| LIPID-N-0644 | PI(20:3/18:0) |
| LIPID-N-0649 | PI(20:4/16:0) |
| LIPID-N-0663 | PI(18:1/20:4) |
| LIPID-N-0665 | PI(18:0/20:5) |
| LIPID-N-0702 | PS(18:0/20:4) |
| LIPID-P-0126 | DG(16:0/18:0/0:0) |
| LIPID-P-0129 | DG(18:0/18:0/0:0) |
| LIPID-P-0144 | DG(16:0/20:1/0:0) |
| LIPID-P-0145 | DG(16:1/20:0/0:0) |
| LIPID-P-0152 | DG(14:0/18:2/0:0) |
| LIPID-P-0159 | DG(18:0/18:2/0:0) |
| LIPID-P-0163 | DG(20:0/18:2/0:0) |
| LIPID-P-0202 | DG(18:4/18:1/0:0) |
| LIPID-P-0205 | DG(18:2/20:3/0:0) |
| LIPID-P-0222 | LPC(12:0/0:0) |
| LIPID-P-0223 | LPC(14:0/0:0) |
| LIPID-P-0225 | LPC(18:0/0:0) |
| LIPID-P-0230 | LPC(16:1/0:0) |
| LIPID-P-0237 | LPC(18:3/0:0) |
| LIPID-P-0241 | LPC(22:4/0:0) |
| LIPID-P-0263 | LPE(0:0/16:0) |
| LIPID-P-0267 | LPE(0:0/24:0) |
| LIPID-P-0272 | LPE(0:0/22:1) |
| LIPID-P-0280 | LPE(0:0/22:4) |
| LIPID-P-0314 | PC(18:0/22:0) |
| LIPID-P-0429 | PC(20:5/12:0) |
| LIPID-P-0452 | PC(22:4/18:1) |
| LIPID-P-0471 | PC(18:1/20:5) |
| LIPID-P-0588 | PE(22:2/12:0) |
| LIPID-P-0676 | PG(18:0/16:0) |
| LIPID-P-0706 | PS(20:4/20:0) |
